# Reprogramming human A375 amelanotic melanoma cells by catalase overexpression: Upregulation of antioxidant genes correlates with regression of melanoma malignancy and with malignant progression when downregulated

**DOI:** 10.18632/oncotarget.9273

**Published:** 2016-05-10

**Authors:** Candelaria Bracalente, Irene L. Ibañez, Ariel Berenstein, Cintia Notcovich, María B. Cerda, Fabio Klamt, Ariel Chernomoretz, Hebe Durán

**Affiliations:** ^1^ Departamento de Micro y Nanotecnología, Comisión Nacional de Energía Atómica, San Martín, Buenos Aires, Argentina; ^2^ Consejo Nacional de Investigaciones Científicas y Tecnológicas, Buenos Aires, Argentina; ^3^ Fundación Instituto Leloir and Departamento de Física, Facultad Ciencias Exactas y Naturales, Universidad de Buenos Aires, Buenos Aires, Argentina; ^4^ Laboratório de Bioquímica Celular, Departamento de Bioquímica, Instituto de Ciências Básicas da Saúde, Universidade Federal do Rio Grande do Sul, Porto Alegre, Brasil; ^5^ Escuela de Ciencia y Tecnología, Universidad Nacional de San Martín, San Martín, Buenos Aires, Argentina

**Keywords:** melanoma, AOS network, melanogenesis, metastasis, microarrays

## Abstract

Reactive oxygen species (ROS) are implicated in tumor transformation. The antioxidant system (AOS) protects cells from ROS damage. However, it is also hijacked by cancers cells to proliferate within the tumor. Thus, identifying proteins altered by redox imbalance in cancer cells is an attractive prognostic and therapeutic tool. Gene expression microarrays in A375 melanoma cells with different ROS levels after overexpressing catalase were performed. Dissimilar phenotypes by differential compensation to hydrogen peroxide scavenging were generated. The melanotic A375-A7 (A7) upregulated TYRP1, CNTN1 and UCHL1 promoting melanogenesis. The metastatic A375-G10 (G10) downregulated MTSS1 and TIAM1, proteins absent in metastasis. Moreover, differential coexpression of AOS genes (EPHX2, GSTM3, MGST1, MSRA, TXNRD3, MGST3 and GSR) was found in A7 and G10. Their increase in A7 improved its AOS ability and therefore, oxidative stress response, resembling less aggressive tumor cells. Meanwhile, their decrease in G10 revealed a disruption in the AOS and therefore, enhanced its metastatic capacity.

These gene signatures, not only bring new insights into the physiopathology of melanoma, but also could be relevant in clinical prognostic to classify between non aggressive and metastatic melanomas.

## INTRODUCTION

Reactive oxygen species (ROS) has been widely implicated in tumor transformation. Indeed, ROS and antioxidants were proposed as potential therapeutic tools. However, changes in the redox balance may have different impact on tumor cells. Therefore, it is important to study how ROS status and the antioxidant system (AOS) of tumor cells affect the global response of gene expression.

It has been well established that most cancer cells, including melanoma, are characterized by high ROS levels that induce mandatory steps of cancer initiation and progression [1–9]. Disruption of the normal redox balance by deregulation in AOS proteins, such as superoxide dismutase (SOD), catalase and glutathione and thioredoxin system proteins, was associated with cancer development [10–13]. Dysfunction of ROS-producing systems coevolves with the AOS to a new redox balance leading to the progression from melanocytes to melanoma [14]. Besides, the prooxidant state in melanoma may also induce alterations in proteins involved either in melanogenesis or metastasis.

The oxidative stress exerts strong adaptive pressure on cancer cells, which in order to survive, promote the expression of ROS pathways reprogramming the transcriptome, proteome and metabolism [15]. The reversion of malignant phenotype by overexpression of antioxidant enzymes, such as catalase has been studied [16–19]. However, it is still not clear which groups of genes are overexpressed or down regulated in melanoma cells under this condition.

Besides, melanoma prognosis is still based mainly on histopathological criteria [20]. Thus, to enable earlier diagnosis and prognosis, it would be relevant to define new molecular markers. In this sense, coexpressed genes associated with the AOS response as predictors of melanoma development and its progression have not been proposed yet.

Thus, to identify genes involved in melanoma progression or regression after an AOS response, we developed a human melanoma model with different levels of ROS by stably overexpressing catalase in A375 cells (in press, 2016). Whole genome gene expression patterns were analyzed by microarrays.

Catalase overexpression triggered dissimilar gene expression. Differential compensation to hydrogen peroxide scavenging gave rise to either, melanogenic A375-A7 (A7) or metastatic A375-G10 (G10) phenotypes. In this sense, A7 upregulated genes involved in differentiation such as tyrosinase related protein 1 (TYRP1), contactin 1 (CNTN1) and ubiquitin COOH-terminal hydrolase L1 (UCHL1) [21–24], turning this clone less aggressive. Meanwhile, G10 downregulated genes, promoting an undifferentiated and invasive phenotype. Particularly, this clone decreased metastasis suppressor 1 (MTSS1) and T-Cell lymphoma invasion and metastasis 1 (TIAM1) genes, whose downregulation enhances malignant progression [25–27]. However, the behavior of these proteins in melanoma has received little attention. Moreover, A7 upregulated coexpressed genes associated with hydrogen peroxide metabolism. Downregulation of these genes was correlated with malignant progression, as observed in G10. Thus, changing the expression of one antioxidant gene in A375 melanoma cells triggered different phenotypes by compensatory reprogramming the AOS network.

Therefore, beyond these promising result need further validations in other melanoma models, the upregulation of TYRP1, CNTN1 and UCHL1, the downregulation of MTSS1 and TIAM1 and the coexpression of the AOS genes EPHX2, GSTM3, MGST1, MSRA, TXNRD3, MGST3 and GSR, could be used to classify between non-aggressive and metastatic melanomas.

## RESULTS

### Gene expression profiles

Catalase overexpression on human amelanotic melanoma A375 cells gave rise to a clone with increased polarity related to differentiated melanoma (A7), and another clone (G10) with disrupted polarity associated with malignant progression (in press, 2016). Therefore, differential gene expression was evaluated by microarrays analysis of whole genome in this model. A375 and PCDNA3 cells (A375 cells transfected with the empty vector) were also evaluated, considering their average results as control.

**Figure 1:**
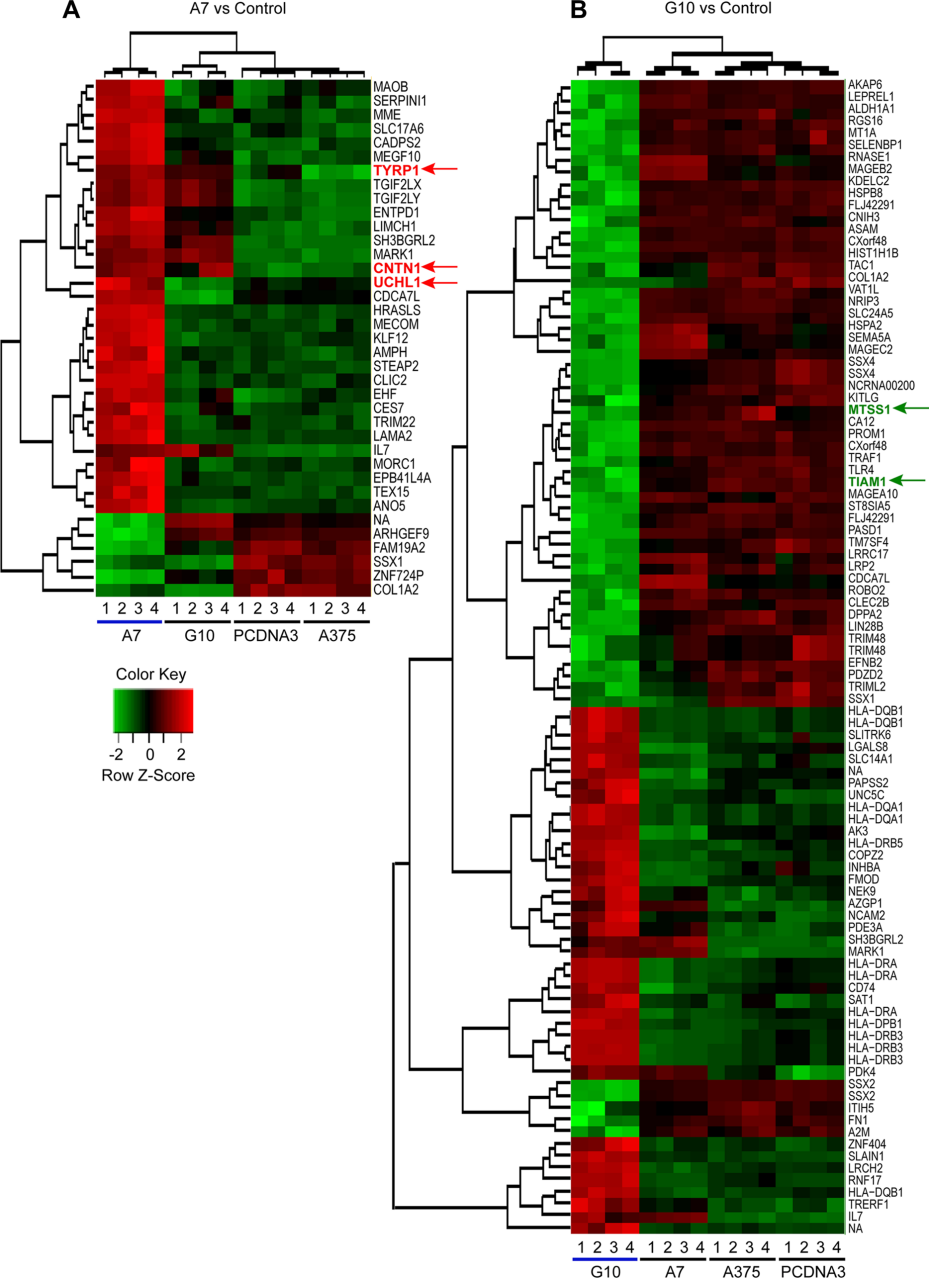
Differential gene expression and clustering analysis of upregulated and downregulated genes in A7 (A) and G10 (B) cells Average result from A375 and PCDNA3 cells were used as control. Genes selected for qPCR analysis are indicated with colored arrows (red for upregulated in A7 vs controls and green for downregulated in G10 vs controls). Analysis was conducted with lfc = 2 and *p* ≤ 0.0001. Key color: red for upregulated and green for downregulated genes.

The analysis showed 6 downregulated genes and 31 upregulated genes from 33297 genes in A7 vs control (Figure 1A) and 57 downregulated and 39 upregulated genes in G10 vs control (Figure 1B). Besides, G10 showed 86 downregulated and 50 upregulated genes vs A7 (Figure S1). No differentially expressed genes were found between controls.

### Functional classification analysis

Regarding DAVID functional annotation clustering (Table S1 and S2), the comparison between A7 and control (*p* < 0.01) showed upregulated genes involved in cell and biological adhesion and basement membrane terms, within which CNTN1 was found. By contrast, A7 did not downregulated any process vs control. Meanwhile, G10 vs A7 (*p* < 0.01), downregulated genes associated with cell projection, axon, cell morphogenesis involved in differentiation and neuron differentiation, neuron projection morphogenesis and cell adhesion terms. Moreover, G10 vs control (*p* < 0.01) downregulated not only most of these same terms but also, those related to regulation of actin cytoskeleton and cell motion. Within these terms, MTSS1, UCHL1 and TIAM1 appeared downregulated. The genes upregulated in G10 vs control and A7 (*p* < 0.01) were associated with a vast amount of processes involved in the immune response. Besides, processes involved in cell adhesion were upregulated in G10 vs control (*p* < 0.01). Regulation of transcription and RNA metabolic processes were upregulated in G10 vs A7 (*p* < 0.01).

### Validation of microarrays by quantitative real-time PCR (qPCR)

Considering the functional classification of differentially expressed genes in A7 and G10 vs control (Table S1), five genes were selected to validate microarrays data by qPCR. The selected genes TYRP1, CNTN1 and UCHL1 were upregulated in A7 vs control, as indicated by red arrows in Figure 1A. These genes are involved in melanocyte differentiation and axon guidance and inversely correlated with malignant progression [21, 24, 28]. On the contrary, MTSS1 and TIAM1, described as downregulated in metastatic cells [25–27], were almost not expressed in G10 vs control as pointed by green arrows in Figure 1B.

**Figure 2:**
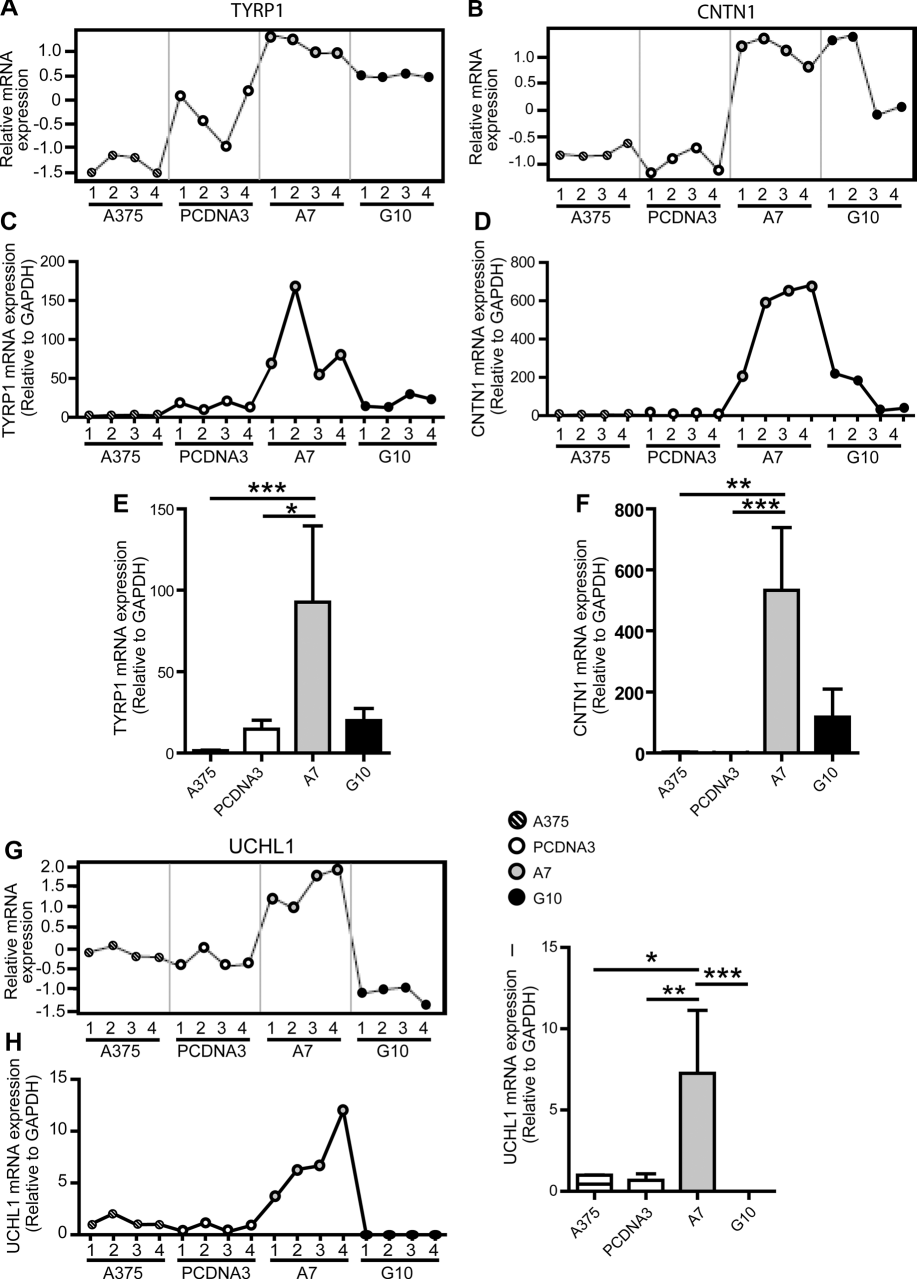
Validation of microarray data by qPCR analysis of selected A7 upregulated genes: TYRP1, CNTN1 and UCHL1 A375 and PCDNA3 cells were used as control cells. Four RNA samples (1–4) were evaluated per condition. The same RNA samples were used for both assays for comparison purposes. (**A–B** and **G**) Microarray gene expression profiles of TYRP1, CNTN1 and UCHL1. (**C–D** and **H**): mRNA expression profiles of selected genes determined by real time PCR relative to GAPDH. (**E–F** and **I**): Results of mean ± SD of qPCR analysis vs controls or G10, *p < 0.05, **p < 0.01, ***p < 0.001.

**Figure 3:**
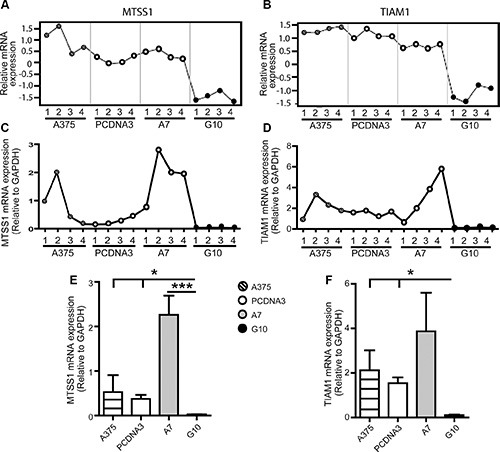
Validation of microarray data by qPCR analysis of selected G10 downregulated genes: MTSS1 and TIAM1 A375 and PCDNA3 cells were used as control cells. Four RNA samples (1–4) were evaluated per condition. The same RNA samples were used for both assays for comparison purposes. (**A–B**) Microarray gene expression profiles of MTSS1 and TIAM1. (**C–D**) mRNA expression profiles of selected genes determined by real time PCR relative to GAPDH. (**E–F**) Results of mean ± SD of qPCR analysis vs controls or A7, *p < 0.05, ***p < 0.001.

Gene expression profiles obtained by qPCR coincided with those obtained by microarrays analysis for all genes in most of the samples evaluated, validating microarrays data. TYRP1, CNTN1 and UCHL1 mRNA expression were upregulated in A7 vs controls (*p* < 0.05) (Figure 2). On the contrary, MTSS1 and TIAM1 mRNA expression were downregulated in G10 vs controls (*p* < 0.05) (Figure 3).

Therefore, results support that overexpression of TYRP1, CNTN1 and UCHL1 in A7 is consistent with its differentiated melanogenic phenotype. Meanwhile, MTSS1 and TIAM1 downregulation in G10 supports the migration and metastatic ability acquired by these cells (in press, 2016).

### Contrasting biological processes by differential coexpressed genes between A7 and G10

The analysis of coexpressed genes performed by GSEA (Gene Set Enrichment Analysis) [29, 30] (Table S3 and S4) showed upregulation of clustered genes related to cell adhesion molecules, peroxisome, apoptosis and melanogenesis in A7 vs control (*p* < 0.05), supporting the differentiated and melanotic phenotype of these cells. Besides, A7 downregulated cell-cell adhesion, regulation of angiogenesis and positive regulation of epithelial cell migration and proliferation involved in wound healing vs control (*p* < 0.05). Interestingly, processes associated with G10 metastatic phenotype as neural crest cell migration, blood vessel endothelial cell migration, intussusceptive angiogenesis and ameboidal cell migration were upregulated in G10 vs A7 (*p* < 0.05). Cell cycle, PPAR signaling pathway and apoptosis were downregulated in G10 vs A7 (*p* < 0.05). Endothelial cell migration, several types of cell-cell adhesion, drug metabolic process and regulation of anti-apoptosis were upregulated in G10 vs control (*p* < 0.05). Consistent with G10 less proliferation and its apolar morphology, cell cycle and regulation of actin cytoskeleton clustered genes were downregulated vs controls (*p* < 0.05).

### Prognostic gene signatures associate A7 with non-aggressive and G10 with high-risk metastatic melanomas

**Figure 4:**
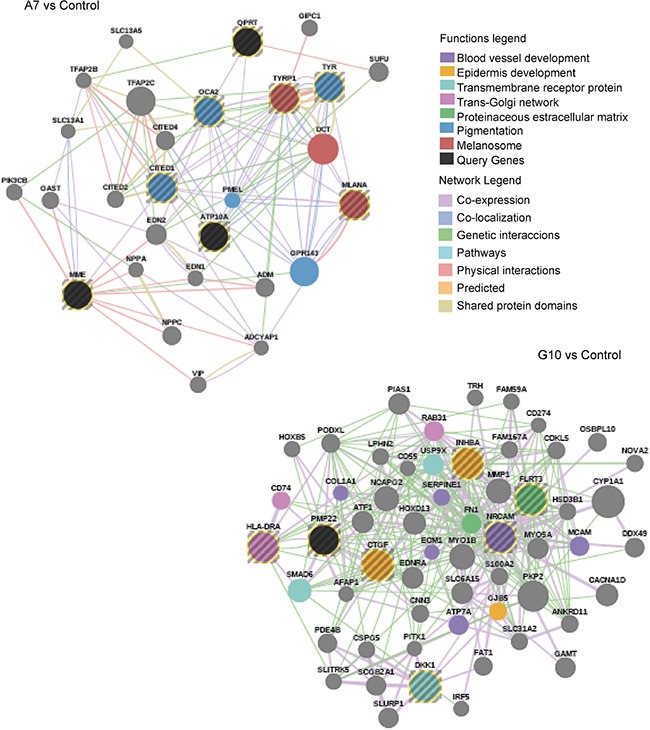
Melanoma prognostic signatures Co-overexpressed clustered genes defined *a priori* from bibliographic prognostic signatures by GSEA in A7 and G10 vs control (average result of A375 and PCDNA3). Query genes are represented by circles with gray stripes. Besides, connections among query genes linked to each other and to neighboring genes (smooth circles) are represented in the figure with different color lines according to their type of interaction. Gene colors represent the functions to which they are associated. Networks are visualized by GeneMania.

The differential phenotypes between A7 and G10 cells correlates with less or more aggressive melanomas. Their gene expression profiles were assessed against coexpressed genes of melanoma prognostic signatures associated with invasion, differentiation, aggressiveness and metastasis (Table 1). A7 cells, unlike G10, coexpressed genes associated with prognostic signatures of less aggressive, non-metastatic and more differentiated melanomas. Particularly A7, overexpressed TYRP1, CITED, TYR, MLANA, ATP10A and OCA2. These genes are involved in melanocytic differentiation and were described as downregulated in aggressive and metastatic melanomas compared to normal melanocytes. Meanwhile, HLA-DRA, INHBA, DKK1, CTGF, PMP22, FLRT3 and NRCAM genes, upregulated in G10, were described as overexpressed in invasive melanomas (Figure 4). These results suggest that differential responses of the AOS network induced by stable catalase overexpression would mediate not only melanocyte differentiation but also invasion and metastasis, supporting *in vitro* and *in vivo* results (in press, 2016).

**Table 1:** Melanoma Prognostic Signature

Prognostic Signature	Number of Genes/Gene Group Total Number	*P* Value	Reference
**Upregulated A7 vs Control**			
Up regulated genes in neural crest development of melanocytes, differentiation and pigmentation and down regulated genes in angiogenesis, neurogenesis, immunomodulation and interaction and remodeling extracellular environment	39/65	0	Jeffs et al., 2009
Genes downregulated in aggressive melanoma cells	24/60	0	Ryu et al., 2007
Genes with low expression in metastatic melanomas vs melanocytes	8/23	0	Riker et al., 2008
Genes with low expression in primary cutaneous melanomas vs melanocytes	12/21	0	Riker et al., 2008
Genes downregulated in metastatic vs primary melanomas	6/38	0,036	Jaeger et al., 2007
**Downregulated in A7 vs Control**			
Genes upregulated in positive metastatic melanoma	69/183	0	Winnepenninckx et al., 2006
Genes upregulated in vertical vs radial growth melanomas	26/48	0	Ryu et al., 2007
Genes downregulated in melanomas with low proliferative and high metastatic capacity	4/5	0,002	Hoek et al., 2006
Genes upregulated in melanomas with low proliferative and high metastatic capacity	4/5	0,004	Hoek et al., 2006
**Upregulated in G10 vs Control**			
Prolonged survival	9/50	0	Bogunovic et al., 2009
Genes upregulated in MGP melanoma cells vs melanocytes	10/29	0	Pfaff Smith et al., 2005
Down regulated genes in neural crest, melanocyte development, differentiation and pigmentation and up regulated genes in angiogenesis, neurogenesis, immunomodulation and interaction and remodeling of extracellular environment	12/31	0,008	Jeffs et al.,2009
Genes upregulated in melanomas with low proliferative and high metastatic capacity	3/5	0,008	Hoek et al., 2006
Genes downregulated in melanomas with low proliferative capacity and high metastatic capacity	2/5	0,008	Hoek et al., 2006
Genes upregulated in vertical vs radial growth melanomas	13/48	0,012	Ryu et al.,2007
**Downregulated in G10 vs Control**			
Genes downregulated in metastatic melanomas vs primary melanomas	4/17	0	Jaeger et al., 2007
Molecular signature of metastatic melanomas	3/11	0,016	Wang et al., 2012

### Catalase overexpression induced differential AOS responses between A7 and G10

**Figure 5:**
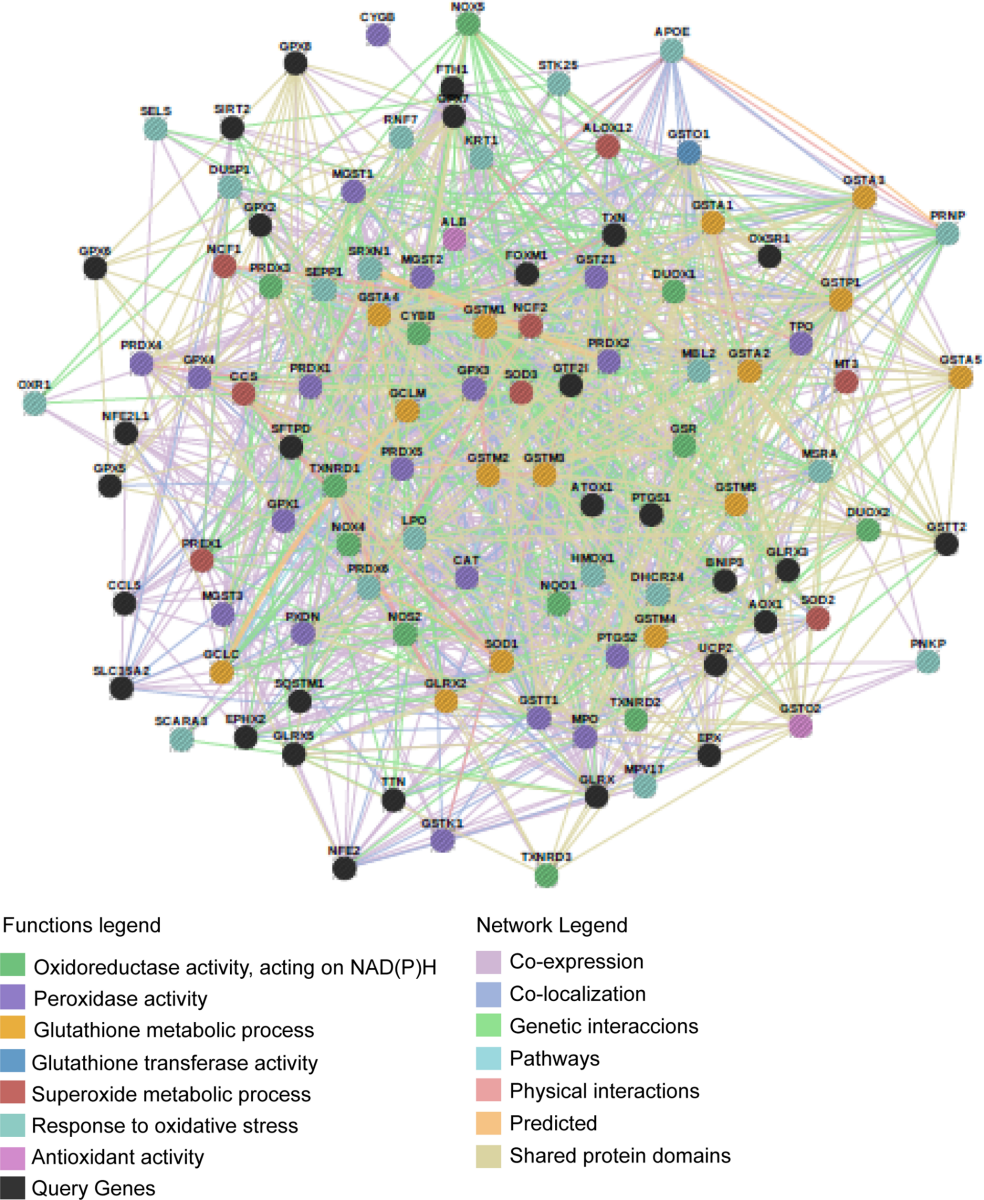
Network of 111 genes associated to the antioxidant system The AOS network was manually designed to be analyzed by GSEA. Connections between genes linked to each other and to neighboring genes are represented in the figure with different color lines according to their type of interaction. Gene colors represent the functions to which they are associated. Network visualized by Gene Mania.

**Figure 6:**
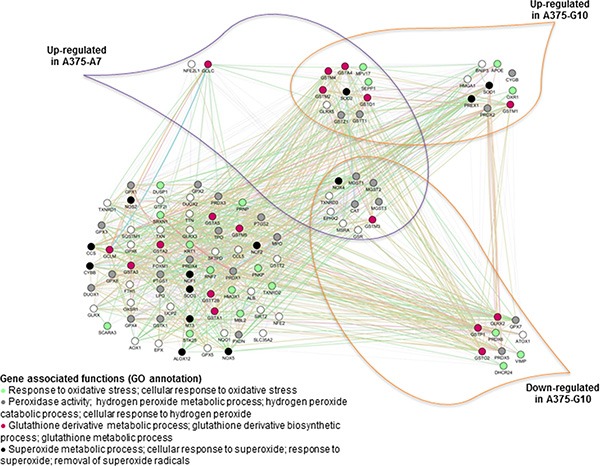
Coexpressed genes of the AOS network in A7 and G10 Coexpression of the AOS genes in A7 and G10 vs control (average result of A375 and PCDNA3). Query genes are represented by circles with gray stripes. Besides, connections among query genes linked to each other are represented in the figure with different color lines according to their type of interaction. Gene colors represent the functions to which they are associated. Network is visualized by GeneMania.

An AOS gene network was manually designed (Figure 5) in order to study possible compensation mechanisms induced by A7 and G10 cells in response to catalase overexpression. The AOS gene network was differentially expressed between A7 and G10 (Figure 6). Remarkably, the coexpressed genes upregulated in A7, GSTM3, NOX4, TXNRD3, EPHX2, MSRA, GSR, CAT, MGST3, MGST2 and MGST1 were downregulated in G10 vs controls (*p* < 0.05). Moreover, G10 also downregulated GPX7, PRDX5, PRDX6, DHCR24, VIMP, ATOX1, GLRX2, GSTP1 and GSTO2 vs controls (*p* < 0.05). Interestingly, A7 did not downregulate any gene of this AOS network. Besides, only A7 upregulated GCLC and NFE2L1. Meanwhile, G10 upregulated GSTM1, SOD1, PREX1, CYGB, PRDX2, OXR1, APOE, BNIP3 and HMGA1 vs controls (*p* < 0.05).

**Figure 7:**
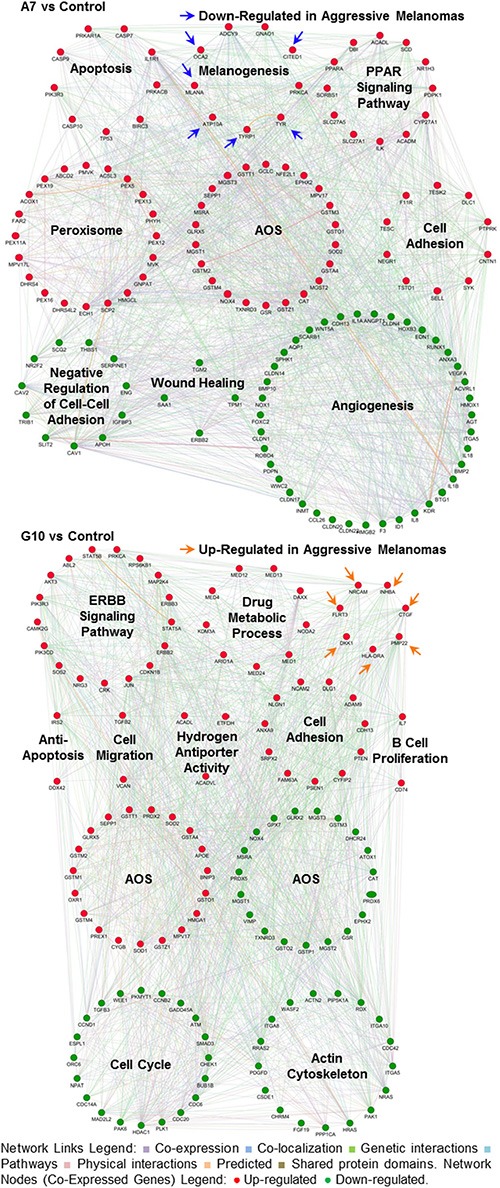
Gene networks associated with signaling pathways and prognostic signatures after reprogramming the AOS network in A7 and G10 Coexpression of genes supports the different phenotypes of A7 and G10 vs control (average result of A375 and PCDNA3). Up or downregulated genes are represented by red or green circles, respectively (Network Nodes). Connections among genes linked to each other are represented in the figure with different color lines according to their type of interaction (Network Links Legend). Arrows show genes from prognostic signatures associated with aggressive melanomas. The upregulation of these genes in A7 supports its less aggressive phenotype while in G10, its more aggressive one. Networks are visualized by GeneMania.

These results indicate that A7 upregulated peroxidase activity, hydrogen peroxide metabolic processes and cellular response to hydrogen peroxide. Meanwhile, these processes were downregulated in G10. Besides, G10 unlike A7, also downregulated certain genes associated with response to oxidative stress and glutathione metabolic and glutathione derivative biosynthetic processes. Finally, the complex gene networks associated with signaling pathways and bibliographic prognostic signatures that were induced by catalase overexpression after reprogramming the AOS network are shown in Figure 7.

Therefore A7, by increasing the metabolism of hydrogen peroxide, reduced more efficiently ROS levels, resembling to less aggressive tumor cells. Meanwhile G10, by having reduced ability to respond to oxidative stress, increased its ROS levels conducting to migration and malignant progression.

## DISCUSSION

This work demonstrated a differential response of the AOS network induced by catalase overexpression in A375 melanoma cells. Moreover, the AOS gene expression profiles found in A7 and G10 correspond with their biological changes, where more or less aggressive melanomas were induced (in press, 2016).

The prooxidant state in melanoma induces alterations in proteins involved in melanogenesis, such as tyrosinase and TYRP1 [31, 32]. A7 cells upregulated coexpressed genes involved in melanogenesis, peroxisome and cell adhesion and downregulated genes of epithelial cell migration. Particularly, A7 upregulated TYRP1, CNTN1 and UCHL1, which participate in melanocyte differentiation and axon guidance [21, 24, 28]. This indicates that A7 changed to a more differentiated and less aggressive melanoma.

TYRP1 is involved in melanin synthesis. Thus, its overexpression in A7, not only at mRNA but also at protein level (in press, 2016), supports the melanogenesis induction. Besides, A7 overexpressed CNTN1, a cell adhesion molecule involved in nervous system development, particularly in oligodendrocyte maturation [23] and neurite extension [33]. This could favor A7 progression into neuronal-like morphology; as evidenced by its polarity (in press, 2016). Reduced UCHL1 was associated with worse outcome in primary melanoma. Besides, primary lesions present higher UCHL1 expression than metastatic lesions [21]. Suppression of UCHL1 was postulated as an early step of melanoma development, given that melanocytes present high levels of UCHL1, while benign nevi lack this protein [34]. Moreover, high levels of MTSS1, an actin-binding protein, inhibited migration in fibroblasts [35] and in glioblastoma cells inhibited cell growth, colony formation, migration and invasion [36]. Introducing wild-type MTSS1 or a non-degradable MTSS1 into breast or prostate cancer cells with low levels of MTSS1 inhibited cell proliferation and migration [37]. Thus, increased MTSS1 in A7 reinforced its milder malignancy. However, there still controversial results about MTSS1 influence in cancer progression [38].

Conversely, G10 upregulated coexpressed genes involved in cell migration and angiogenesis, while downregulated those involved in cell cycle and apoptosis. These results correspond to the ability of G10 to migrate and metastasize (in press, 2016). Certainly, MTSS1 and TIAM1 were almost absent in G10 cells, as described in metastatic and poor prognosis cancers [25, 36, 37, 39–44]. Thus, loss of MTSS1 in G10 confirms its shift to a more aggressive melanoma. TIAM1 is a guanine nucleotide exchange factor that activates Rac (Rac-GEF). It regulates cell shape and invasiveness in epithelial cells and fibroblasts. Metastatic melanoma cells overexpressing TIAM1 turned the mesenchymal phenotype into an epithelial-like phenotype, whereas its downregulation enhanced malignant progression [26, 27]. The almost absence of TIAM1 in G10 supports its increased metastatic ability [26, 45].

Therefore, not only the absence of MTSS1 and TIAM1 expression in G10, but also high levels of CAP1 and cofilin-1 (in press, 2016), could be key regulators in promoting migration and malignant progression in G10. On the contrary, upregulation of TYRP1, CNTN1 and UCHL1 in melanoma could be reversing malignant conditions. Thus, the expression of these genes could be used to classify the progression of melanoma from a non-aggressive and differentiated to a dedifferentiated and metastatic one. These promising results regarding its prognostic significance require further in-depth investigations.

Cells mount a transcriptional AOS response to scavenge the ROS that arise from chemical, physical, and metabolic challenges. This protective program has been shown to reduce carcinogenesis [15]. However, it is also hijacked by established cancers to thrive and proliferate within the hostile tumor microenvironment and to gain resistance against chemo- and radiotherapies. Thus, targeting the AOS response proteins of cancer cells is an attractive therapeutic strategy. Therefore, to understand which proteins of the AOS are exploited by melanoma in order to decrease or increase its malignancy, the AOS network was studied. The AOS network showed 19 coexpressed genes downregulated in G10, while 10 of them were upregulated in A7. Interestingly, A7 did not downregulated any gene.

Regarding those 10 genes upregulated in A7 and downregulated in G10, EPHX2, a cytosolic epoxide hydrolase, was suggested to prevent progression and metastasis in breast cancer [46]. MGST1 is a microsomal glutathione transferase 1, which also displays glutathione peroxidase activity. Overexpression of this enzyme protected MCF7 cells from oxidative damage by decreasing intracellular ROS levels [47, 48]. MSRA (methionine sulfoxide reductase A), which acts as ROS scavenger protecting proteins from oxidation, was found downregulated in metastatic hepatocellular carcinoma [49] and breast cancer [50]. Moreover, MSRA silencing in breast cancer cells increased ROS levels, resulting in extracellular matrix degradation and upregulation of VEGF, which support tumor growth *in vivo* [50]. Furthermore, skin antioxidant network includes not only interceptive antioxidants that dissipate ROS, but also specific repair enzymes such as MSRA, which reverse macromolecular damage. These two mechanisms work together to maintain a delicate redox balance, crucial for homeostasis. In this sense, melanocytes possess high levels of MSRA, MSRB and thioredoxin reductase [51, 52]. Particularly, murine melanoma cells increased the expression of TXNRD3 (thioredoxin reductase 3) during melanogenesis [53]. Downregulation of GSTM3 (glutathione-S-transferase Mu 3) was associated with metastasis in clear-cell renal-cell carcinoma [54] and breast [55] and colon cancer cells [56]. A proteomic analysis of MCF-7 breast cancer cells expressing constitutively active MEK5/Erk5, showed GSTM3 downregulation related to epithelial mesenchymal transition [55]. Besides, GSTM3 and also MGST3 (Microsomal Glutathione S-Transferase 3) were induced in human colon adenoma cells by the chemoprotector butyrate, while not in highly transformed neoplastic colorectal cells. Moreover, butyrate induced catalase in the primary colon non transformed cells [56]. Inhibition of GSR (Glutathione Reductase) activity induced oxidative stress, evidenced by intracellular ROS increase and peroxidation of mitochondrial membrane in melanoma cells. Therefore, the coexpressed upregulated genes EPHX2, GSTM3, MGST1, MSRA, TXNRD3, MGST3 and GSR in A7 would be increasing the ability of A7 cells to respond to oxidative stress. This protection from oxidative damage would be stimulating melanogenesis. These changes in gene expression profile led A7 cells to the acquisition of differentiated features, reversing malignancy. On the other hand, downregulation of these genes in G10 may be related to its worse ability to respond to oxidative stress. Therefore, intracellular ROS increase would be triggering dedifferentiation and so malignant progression.

Concerning the other 9 genes downregulated in G10, ATOX1 (antioxidant-1) is a copper-dependent transcription factor that mediates copper-induced cell proliferation. ATOX1 inhibition reduced copper-stimulated cell proliferation in mouse embryonic fibroblasts and non-small cell lung cancer cells [57, 58]. Adrenocortical carcinoma was characterized by silencing of genes on chromosome 11q13, including PRDX5 (peroxiredoxin 5) [59]. PRDX6 presented reduced levels in papillary thyroid carcinomas compared to non-neoplastic tissues. A correlation between the presence of lymph node metastasis and low PRDX6 levels was also described [60]. The progression from low-grade to high-grade prostate carcinoma and metastases is mediated by down-regulation of the androgen receptor target genes, including DHCR24 (24-Dehydrocholesterol Reductase) [61]. Reduced mRNA and protein expression of GSTP1 (glutathione S-transferase-pi) was found in neuroblastoma cell lines and high risk NB tumor samples [62]. Likewise, inverse correlation between GSTP1 expression and Barrett's esophageal metaplasia-dysplasia-adenocarcinoma sequence was demonstrated [63]. GPX7 (glutathione peroxidase 7) may function as tumor suppressor. It is frequently silenced in esophageal adenocarcinoma (EAC). The dysfunction of GPX7 in esophageal cells increases ROS levels and oxidative DNA damage, which are common risk factors for Barrett's esophagus and EAC [64, 65]. Thus, downregulation of ATOX1, PRDX5, PRDX6, DHCR24, GSTP1 and GPX7 in G10 supports its worse outcome.

Regarding the two genes that were upregulated only in A7, GCLC (glutamate-cysteine ligase, catalytic subunit) and NFE2L1 (nuclear factor erythroid-derived 2-like 1), high expression of GCLC, the rate-limiting enzyme in glutathione synthesis, was associated with lower intracellular ROS and cell proliferation in 36 melanoma cell lines and also with better 5-year overall survival in patients with melanoma. Besides, invasiveness and switch from E-cadherin to N-cadherin expression were promoted in melanoma cells with lower GCLC expression [66]. In breast cancer, GCLC expression was inversely correlated with malignancy [67]. NFE2L1 functions as transcription factor that binds to antioxidant response element (ARE) of DNA. Its somatic inactivation is involved in hepatic cancer induction. Hepatocytes lacking NFE2L1 exhibited increased oxidative stress and impaired expression of antioxidant genes [68, 69]. In human skin tumors NFE2L1 was lower than in normal skin [70]. The involvement of NF2L1 has also been described in osteoblast differentiation [71, 72]. Thus, GCLC and NFE2L1 upregulation in A7 may contribute to both the increased ROS scavenging capacity compared with G10 and controls and its more differentiated and less aggressive melanoma.

Therefore, as far as we are concerned, this is the first time that a human melanoma model allowed to define a group of genes from the AOS downregulated by melanoma cells to take advantage to spread new areas and metastasize. Meanwhile, upregulation of this group of genes reversed malignant features.

## MATERIALS AND METHODS

### Cell culture and transfection of catalase

Low-passages human amelanotic melanoma cell line A375 was kindly given by Dr. E. Medrano (Huffington Center on Aging, Departments of Molecular & Cellular Biology and Dermatology, Baylor College of Medicine, Houston, Texas, USA). All the experiments with these cells were performed with less than 5 passages from thawing. Cells were cultured as previously described [73]. Stable transfected cells were maintained in identical conditions with 700 μg/ml geneticin (Sigma). Cells were regularly tested to be mycoplasma-free.

A375-A7 and A375-G10 cells overexpressing catalase (in press, 2016), referred in this work as A7 and G10 respectively, were used. Due to their intriguing differential responses to catalase overexpression already described in the mentioned paper, whole genome microarrays experiments were performed. A375-PCDNA3 (transfected with empty vector, referred here as PCDNA3) and A375 cells were used as controls in the microarrays experiments.

### RNA isolation and microarray experiment

Total RNA samples were isolated from cultured cells using RNAspin Mini RNA Isolation Kit (GE Healthcare), following manufacturer instructions. RNA quantity and quality were determined by NanoDrop2000 photometer (Thermo). RNA integrity was assessed evaluating the ~2:1 ratio of 28S:18S bands in 1% agarose gel electrophoresis (Figure S2). Four biological replicates were used per condition.

Biotin-labeled cRNA was generated and hybridised to Affymetrix Human Genome Chip (GeneChip^®^ Human Gene 1.0 ST) following the manufacturer's instructions (Affymetrix, Santa Clara, USA) by the Agricultural Plant Physiology and Ecology Research Institute (IFEVA), University of Buenos Aires, Argentina.

### Microarray analysis and data processing

Differential gene expression among human amelanotic melanoma cells, A375, and the two established catalase-overexpressing clones with different phenotypes (A7, melanotic and non-invasive and G10, amelanotic and invasive) was evaluated by the bioinformatic analysis of whole genome microarrays (GeneChip^®^ Human Gene 1.0 ST Array, Affymetrix). A375 and PCDNA3 cells were used as controls.

The analysis was performed by using the R programming language (2.12.0) [74] and different tools of Bioconductor [75]. The libraries “affy”, “limma”, “oligo”, “affxparser”, “Iranges”, “gplots”, “Biobase”, “Biostrings”, “cluster”, “hugene10stprobeset.db”, “Go.db”, “preprocess Core”, hugene10sttranscriptcluster.db”, “pd.hugene.1.0.st.v1”, “pd.hugene.1.1.st.v1”, “org.Hs.eg.db”, “annotate” and KEGG.db” were used. Background correction and normalization of data were performed by Robust Multi-array Average (rma) both for probe set and core. Differential gene expression was evaluated by Limma package (Linear Models for Microarray Data). Log fold change (lfc) and *p* value parameters were established. In order to determine those genes to be analyzed for functional classification or qPCR validation a 1 and 2 lfc were used respectively. Both analyses were performed with a *p* < 0.001.

The differential genes obtained were functionally classified by DAVID (Database for Annotation, Visualization and Integrated Discovery) [76, 77]. DAVID Functional Annotation Clustering tool was performed by using the annotation terms: Disease (OMIM_disease), Gene Ontology (GOTERM_BP_FAT, GOTERM_CC_FAT, GOTERM_MF_FAT), Pathways (BBID, BIOCARTA, KEGG_PATHWAY, REACTOME_PATHWAY) and Tissue Expression (UP_TISSUE). The classification stringency was selected as medium and the options were selected as default.

Significant and concordant differences between phenotypes were evaluated by GSEA (Gene Set Enrichment Analysis) [29] with *a priori* defined gene sets collected from Gene Ontology Database [78] (131 gene groups) and KEGG (Kyoto Encyclopedia of Genes and Genomes) [79–81] (19 gene groups). These selected gene sets are associated with cell proliferation, melanoma, cell cycle, melanogenesis, apoptosis, cell adhesion, vascularization, angiogenesis, peroxisome, cell migration, regulation of actin cytoskeleton, autophagy regulation, oxidative stress, invasion, cell motility, DNA damage response, drug exportation, drug metabolism, immune response and inflammation (Table S5). An additional set of 111 genes related to the antioxidant system [82–85] was manually defined, visualized by GeneMANIA database [86] and studied under the same criteria (Figure 5 and Table S6). This type of analysis was also performed with 39 bibliographic predictive gene signatures of melanomas, associated with invasion, differentiation, aggressiveness and metastasis [87–98] (Table S7). Coexpressed genes obtained by the analysis were visualized by GeneMANIA database [86] via GeneMANIA web or via Cytoscape plugin [99].

### Validation of microarrays data by quantitative real-time PCR (qPCR)

In order to select genes differentially expressed to validate the microarray results by qPCR, a 2 lfc and *p* < 0.001 were used. The selected genes were TYRP1, CNTN1, UCHL1, MTSS1 and TIAM1. Human GAPDH was used as normalization control. Total RNA samples isolated for microarray experiments were used to validate microarray experiments by qPCR. The cDNA was synthesized using polymerase reverse transcriptase (SuperScript^™^ II, Invitrogen) following manufacturer's indications. Four biological replicates were used per condition.

To perform qPCR 15 μl final reaction volume, 7 μl Master mix (Maxima SYBR Green qPCR Master Mix, Biotium 2X), 0.2 μl primers 10 μM, 3 μl of cDNA 1:50 and free nuclease water (Biodynamics) were used. Sequences of primers (all from Invitrogen) and cycling conditions are detailed in Table 2. Quantitative real-time PCR assays were carried out in a QIAGEN's q PCR cycler. The Pfaffl mathematical model for relative quantification was used to calculate the mRNA expression level (normalized to GAPDH) [8].

**Table 2:** Selected genes for qPCR microarray validation

Gene	5′	Forward Primer	3′	40 Cycles	
		Reverse Primer		Tm (°C/s)	Extension (°C/s)
**TYRP1**	gctccagacaacctggga cagtgaggagaggctggtt			58/25	72/20
**CNTN1**	caacaaaaccatatcctgctga agatcactgcctatgtccacct			55/20	72/30
**UCHL1**	gacttattcacgcagtggc gatggacgaatgcctttt			52/20	72/20
**MTSS1**	agaaagcccgccaagag ccgcagcatagagatgaaggta			55/20	72/15
**TIAM1**	gtggggtctggatactacc gcttcggttccctctc			49/20	72/15
**GAPDH**	cccactcctccacctttgac cataccaggaaatgagcttgacaa			60/20	72/20

## SUPPLEMENTARY FIGURES AND TABLES














