# Supplementary material for: Reprogramming human A375 amelanotic melanoma cells by catalase overexpression: Upregulation of antioxidant genes correlates with regression of melanoma malignancy and with malignant progression when downregulated
Source: Oncotarget. 2016 May 10;7(27):41154–71. doi: 10.18632/oncotarget.9273 (PMC5173049; doi:10.18632/oncotarget.9273)
Supplement: Supplementary file 1 [file oncotarget-07-41154-s001.pdf]

Reprogramming human A375 amelanotic melanoma cells by catalase overexpression: Upregulation of antioxidant genes correlates with regression of melanoma malignancy and with malignant progression when downregulated

Supplementary Materials

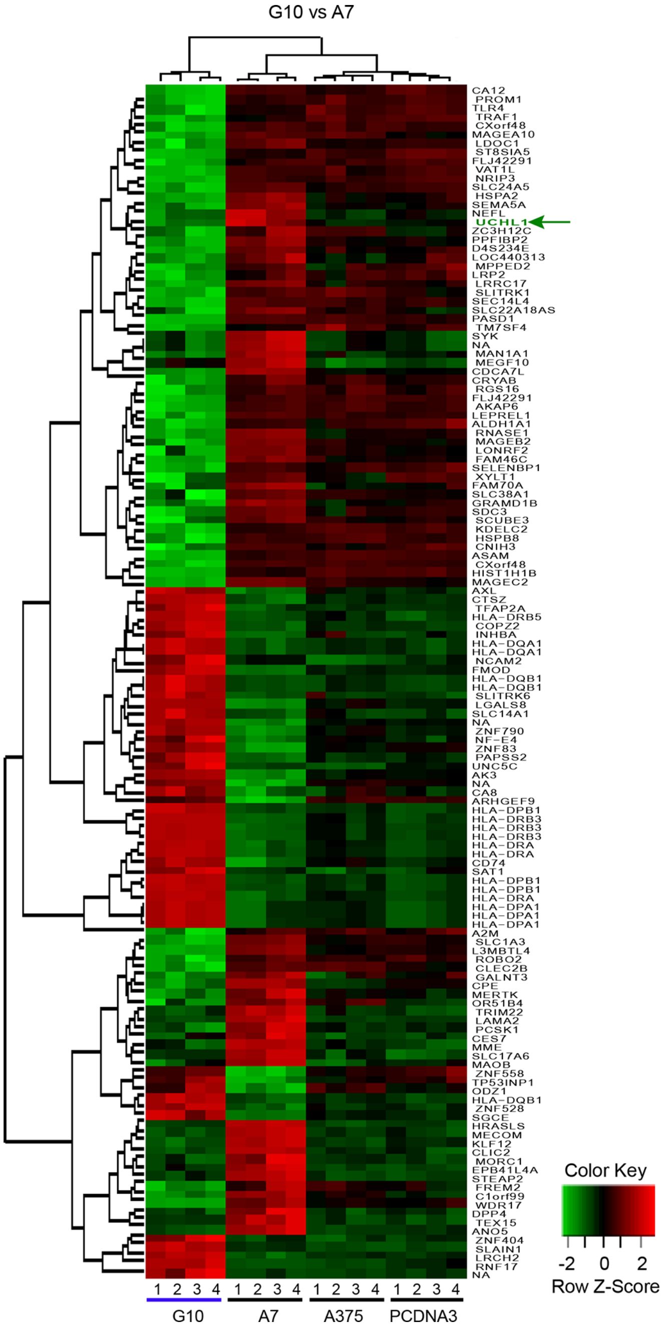

Supplementary Figure S1: Differential gene expression and clustering analysis of upregulated and downregulated genes in G10 vs A7 cells. Average result from A375 and PCDNA3 cells were used as control. Gene selected for qPCR analysis is indicated with green arrow. Analysis was conducted with lfc = 2 and $p \leq 0.0001$. Key color: red for upregulated and green for downregulated genes.

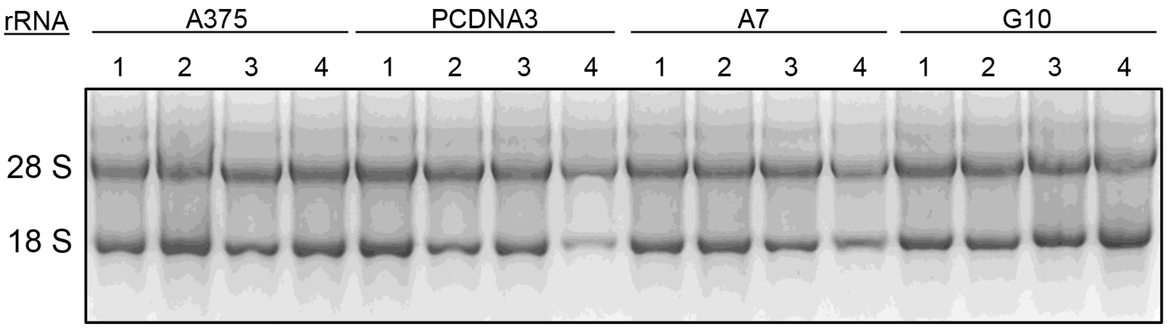

Supplementary Figure S2: RNA integrity. Agarose gel electrophoresis of total RNA samples. Image shows two bands corresponding to the ribosomal RNA (rRNA) subunits (28S and 18S) of total RNA extracted from A375, PCDNA3, A7 and G10 cells. Number indicates replicates samples.

Supplementary Table S1: DAVID Functional Enrichment Analysis. Up and Downregulation of coexpressed clustered genes defined a priori from KEGG and GO. Table shows clustered genes significantly enriched with a ***p*** Value < 0.01 and FDR (%) < 20. See Supplementary_Table_S1

Supplementary Table S2: Significant processes obtained after DAVID analysis with their corresponding gene symbols and gene titles. See Supplementary_Table_S2

Supplementary Table S3: Gene Set Enrichment Analysis Report. Up and Downregulation of coexpressed clustered genes defined a priori from KEGG and GO. Table shows clustered genes significantly enriched with a *p* Value < 0.05 and FDR (%) < 25. See Supplementary_Table_S3

Supplementary Table S4: Significant processes obtained after GSEA analysis with their corresponding gene symbols and gene titles. See Supplementary_Table_S4

Supplementary Table S5: List of selected biological processes to analyze by GSEA. See Supplementary_Table_S5

Supplementary Table S6: List of 111 selected genes of the antioxidant system with their corresponding gene titles to analyze by GSEA. See Supplementary_Table_S6

Supplementary Table S7: List of selected melanoma prognostic signatures to analyze by GSEA

Melanoma Signature	Reference
Up regulated genes in melanomas of patients with prolonged survival<br>Down regulated genes in melanomas of patients with prolonged survival	Bogunovic et al. 2009
Up regulated genes in melanomas with high proliferative capacity and low metastatic capacity<br>Up regulated genes in melanomas with low proliferative capacity and with high metastatic capacity<br>Down regulated genes in melanomas with high proliferative capacity and low metastatic capacity<br>Down regulated genes in melanomas with low proliferative capacity and with high metastatic capacity	Hoek et al. 2006
Up regulated genes in metastatic melanomas vs primary melanomas<br>Down regulated genes in metastatic melanomas vs primary melanomas<br>Down regulated genes in metastatic melanomas vs primary melanomas at a p value < 0.01<br>Down regulated genes in metastatic melanomas vs primary melanomas at a p value = 0.01<br>Down regulated genes in metastatic melanomas vs primary melanomas at a p value = 0.02<br>Down regulated genes in metastatic melanomas vs primary melanomas at a p value = 0.03<br>Down regulated genes in metastatic melanomas vs primary melanomas at a p value = 0.04<br>Down regulated genes in metastatic melanomas vs primary melanomas at a p value = 0.05<br>Oncogenes in melanoma	Jaeger et al. 2007
Up regulated genes associated with neural crest, melanocytic development, differentiation and pigmentation and Downregulated genes associated with angiogenesis, neurogenesis, immunomodulation, interaction and remodeling of extracellular environment<br>Down regulated genes associated with neural crest, melanocytic development, differentiation and pigmentation and Upregulated genes associated with angiogenesis, neurogenesis, immunomodulation, interaction and remodeling of extracellular environment	Jeffs et al. 2009
Up regulated genes in melanomas with good prognosis<br>Down regulated genes in melanomas with good prognosis	John et al. 2008
Genes with increased expression in melanomas of patients with prolonged survival<br>Genes with increased expression in melanomas of patients with short survival	Mandruzzato et al. 2006
Up regulated genes in melanoma MGP cells vs melanocytes<br>Up regulated genes in advanced melanomas<br>Down regulated genes in advanced melanomas	Pfaff Smith et al. 2005
Up regulated genes in metastatic melanomas<br>Genes with high expression in primary cutaneous melanomas vs melanocytes<br>Genes with high expression in metastatic melanomas vs melanocytes<br>Down regulated genes in metastatic melanomas<br>Genes with low expression in primary cutaneous melanomas vs melanocytes<br>Genes with low expression in metastatic melanomas vs melanocytes<br>Genes with decreased expression in metastatic melanomas vs primary cutaneous melanomas<br>Tumor supresor genes in melanoma	Riker et al. 2008
Up regulated genes in vertical growth vs radial growth<br>Down regulated genes in aggressive melanomas	Ryu et al. 2007
Gene markers of melanoma	Schramm et al. 2011
Overexpressed genes in melanoma	Talantov et al. 2005
Molecular gene signature of metastatic melanomas	Wang et al. 2012
Up regulated genes in positive metastasis<br>Down regulated in positive metastasis	Winnepenninckx et al. 2006
