# Supplementary material for: Reprogramming human A375 amelanotic melanoma cells by catalase overexpression: Upregulation of antioxidant genes correlates with regression of melanoma malignancy and with malignant progression when downregulated
Source: Oncotarget. 2016 May 10;7(27):41154–71. doi: 10.18632/oncotarget.9273 (PMC5173049; doi:10.18632/oncotarget.9273)
Supplement: Supplementary file 2 [file oncotarget-07-41154-s002.docx]

**Table S1. DAVID Functional Enrichment Analysis.** Up and Downregulation of coexpressed clustered genes defined *a priori* from KEGG and GO. Table shows clustered genes significantly enriched with a pValue <0.01 and FDR (%) <20.

**A. Cluster:**

**E. score Category**

**Term**

**PValue Genesa**

**Genes up or down reg/total**

**FDR (%)**

**Upregulated A7 vs Control**

GOTERM_BP_FAT GO:0007155 Cell Adhesion 0,00560274 LAMA2, CDH7, LAMB3, PCDHB8, PCDHB5, MAGI1, **CNTN1**, CNTN3, ENTPD1, MEGF10, SYK

11/77 8.340508

1:1.7308

GOTERM_BP_FAT GO:0022610 Biological Adhesion 0,00565829 LAMA2, CDH7, LAMB3, PCDHB8, PCDHB5, MAGI1, **CNTN1**, CNTN3, ENTPD1, MEGF10, SYK

11/77 8.4198411

6:0.9854 GOTERM_CC_FAT GO:0005604 Basement Membrane 0,01038873 LAMA2, LAMB3, CCDC80, ENTPD1 4/75 12.16233

7:0.9436 GOTERM_BP_FAT GO:0007267 Cell-Cell Signaling 0,00629714 PCSK1, SLC17A6, PCDHB5, IL7, FGF14, MAOA, MME, CHRNA6, AMPH, SYK

4/77 9.3276437

**Upregulated G10 vs Control**

KEGG_PATHWAY hsa05310 Asthma 9.1035E-17

Antigen Processing And

HLA-DQB1, HLA-DRB3, HLA-DRB5, HLA-DPA1, HLA-DPB1, HLA-DMB, HLA-DOA, HLA-DQA2, HLA-DOB, HLA-DMA, HLA-DQA1, HLA-DRA

HLA-DQB1, HLA-DRB3, HLA-DMB, HLA-DMA,

12/43 1.110E-13

GOTERM_BP_FAT GO:0002504

Presentation Of Peptide Or Polysaccharide Antigen Via MHC Class II

1.0897E-16

HLA-DQA2, HLA-DQA1, CD74, HLA-DRB5, HLA- DPA1, HLA-DPB1, HLA-DOA, HLA-DOB, HLA- DRA

13/131 1.776E-13

GOTERM_CC_FAT GO:0042613 MHC Class II Protein Complex 3.8288E-16

HLA-DQB1, HLA-DRB3, HLA-DRB5, HLA-DPA1, HLA-DPB1, HLA-DMB, HLA-DOA, HLA-DQA2, HLA-DOB, HLA-DMA, HLA-DQA1, HLA-DRA

12/112 4.107E-13

KEGG_PATHWAY hsa05330 Allograft Rejection 1.5142E-15

HLA-DQB1, HLA-DRB3, HLA-DRB5, HLA-DPA1, HLA-DPB1, HLA-DMB, HLA-DOA, HLA-DQA2, HLA-DOB, HLA-DMA, HLA-DQA1, HLA-DRA

12/43 1.565E-12

KEGG_PATHWAY hsa05332 Graft-Versus-Host Disease 4.2579E-15

HLA-DQB1, HLA-DRB3, HLA-DRB5, HLA-DPA1, HLA-DPB1, HLA-DMB, HLA-DOA, HLA-DQA2, HLA-DOB, HLA-DMA, HLA-DQA1, HLA-DRA

12/43 4.241E-12

KEGG_PATHWAY hsa04940 Type I Diabetes Mellitus 1.0618E-14

HLA-DQB1, HLA-DRB3, HLA-DRB5, HLA-DPA1, HLA-DPB1, HLA-DMB, HLA-DOA, HLA-DQA2, HLA-DOB, HLA-DMA, HLA-DQA1, HLA-DRA

12/43 1.071E-11

GOTERM_MF_FAT GO:0032395 MHC Class II Receptor Activity 3.0998E-14

GOTERM_CC_FAT GO:0042611 MHC Protein Complex 5.1051E-14

HLA-DQB1, HLA-DRB3, HLA-DPA1, HLA-DPB1, HLA-DOA, HLA-DQA2, HLA-DOB, HLA-DMA, HLA- DQA1, HLA-DRA

HLA-DQB1, HLA-DRB3, HLA-DMB, HLA-DMA,

HLA-DQA2, HLA-DQA1, AZGP1, HLA-DRB5, HLA- DPA1, HLA-DPB1, HLA-DOA, HLA-DOB, HLA- DRA

10/121 3.997E-11

13/112 6.313E-11

KEGG_PATHWAY hsa04672 Intestinal Immune Network For

6.9108E-14

IgA Production

HLA-DQB1, HLA-DRB3, HLA-DRB5, HLA-DPA1, HLA-DPB1, HLA-DMB, HLA-DOA, HLA-DQA2, HLA-DOB, HLA-DMA, HLA-DQA1, HLA-DRA

12/43 6.944E-11

1:11.9026

HLA-DQB1, HLA-DRB3, HLA-DRB5, HLA-DPA1,

KEGG_PATHWAY hsa05320 Autoimmune Thyroid Disease 1.11759E-13 HLA-DPB1, HLA-DMB, HLA-DOA, HLA-DQA2, HLA-DOB, HLA-DMA, HLA-DQA1, HLA-DRA

12/43 1.124E-10

KEGG_PATHWAY hsa04514 Cell Adhesion Molecules (CAMs) 8.9824E-13

HLA-DQB1, F11R, HLA-DRB3, CLDN10, HLA- DMB, HLA-DMA, HLA-DQA2, HLA-DQA1,

NCAM2, HLA-DRB5, HLA-DPA1, HLA-DPB1, HLA- DOA, HLA-DOB, HLA-DRA

HLA-DQB1, HLA-DRB3, HLA-DMB, HLA-DMA,

15/43 9.033E-10

GOTERM_BP_FAT GO:0019882 Antigen Processing And

Presentation

Antigen Processing And

KEGG_PATHWAY hsa04612

Presentation

9.6868E-13

1.1765E-12

HLA-DQA2, HLA-DQA1, CD74, AZGP1, HLA- DRB5, HLA-DPA1, HLA-DPB1, HLA-DOA, HLA- DOB, HLA-DRA

HLA-DQB1, HLA-DRB3, HLA-DMB, HLA-DMA, HLA-DQA2, HLA-DQA1, CD74, HLA-DRB5, HLA- DPA1, HLA-DPB1, HLA-DOA, HLA-DOB, HLA- DRA

14/131 1.5139E-9

13/43 1.1831E-9

KEGG_PATHWAY hsa05416 Viral Myocarditis 5.3644E-12

HLA-DQB1, HLA-DRB3, HLA-DRB5, HLA-DPA1, HLA-DPB1, HLA-DMB, HLA-DOA, HLA-DQA2, HLA-DOB, HLA-DMA, HLA-DQA1, HLA-DRA

12/43 5.3949E-9

KEGG_PATHWAY hsa05322 Systemic Lupus Erythematosus 2.2528E-10

HLA-DQB1, HLA-DRB3, HLA-DRB5, HLA-DPA1, HLA-DPB1, HLA-DMB, HLA-DOA, HLA-DQA2, HLA-DOB, HLA-DMA, HLA-DQA1, HLA-DRA

12/43 2.2656E-7

REACTOME_PATH

WAY REACT_6900 Signaling In Immune System 8.7564E-9

HLA-DQB1, F11R, HLA-DRB3, HLA-DMB, PTEN, HLA-DMA, HLA-DQA2, HLA-DQA1, HLA-DRB5, HLA-DPA1, HLA-DPB1, HLA-DOA, HLA-DOB, PTENP1, HLA-DRA

14/25 5.7973E-6

GOTERM_BP_FAT GO:0006955 Immune Response 3.2339E-4

UP_TISSUE Blood 0,005075

GOTERM_BP_FAT GO:0016339 Calcium-Dependent Cell-Cell

3.8524E-13

Adhesion

GOTERM_BP_FAT GO:0007156 Homophilic Cell Adhesion 2.6609E-11

GOTERM_BP_FAT GO:0016337 Cell-Cell Adhesion 1.4227E-10

HLA-DQB1, IFITM2, IL7, HLA-DRB3, HLA-DMB, HLA-DQA2, HLA-DMA, HLA-DQA1, CD74, IL31RA, AZGP1, HLA-DRB5, SEMA3C, HLA- DPA1, HLA-DPB1, HLA-DOA, HLA-DOB, HLA- DRA

HLA-DQB1, CTSZ, TYRP1, GYPE, HLA-DRB3, PDE3A, HLA-DMB, HLA-DQA2, HLA-DQA1, CYFIP2, HLA-DRB5, HLA-DPB1, SLC14A1, HLA- DRA

PCDHB9, CDH13, PCDHB7, PCDHB5, PCDHB6, PCDHB3, PCDHB16, PCDHB2, PCDHB14, PCDHB11

PCDHB9, PCDHB7, PCDHB8, PCDHB5, PCDHB6, PCDHB3, PCDHB15, PCDHB2, PCDHB14, PCDHB12, PCDHB11, CDH13, PCDHB18, PCDHB16, PCDHB17

PCDHB9, PCDHB7, PCDHB8, PCDHB5, PCDHB6, PCDHB3, PCDHB15, PCDHB2, PCDHB14, CLDN10, PCDHB12, PCDHB11, GPR98, CDH13, NCAM2, PCDHB18, PCDHB16, PCDHB17, CYFIP2

18/131 0.504254

14/158 5.673873

10/131 6.021E-10

15/131 4.1589E-8

19/131 2.2237E-7

GOTERM_BP_FAT GO:0007416 Synaptogenesis 5.0725E-10 PCDHB9, MYO6, PCDHB5, PCDHB6, PCDHB3, PCDHB16, PCDHB2, PCDHB14, PCDHB11

PCDHB9, PTPRK, F11R, PCDHB7, PCDHB8, PCDHB5, PCDHB6, PCDHB3, PCDHB15,

9/131 7.9281E-7

2:6.1167

GOTERM_BP_FAT GO:0007155 Cell Adhesion 4.5985E-8

GOTERM_BP_FAT GO:0022610 Biological Adhesion 4.7243E-8

PCDHB2, PCDHB14, CLDN10, PCDHB12, PCDHB11, GPR98, AZGP1, NCAM2, CDH13, PCDHB18, PCDHB16, PCDHB17, CYFIP2, SGCE, EMB, CYR61

PCDHB9, PTPRK, F11R, PCDHB7, PCDHB8, PCDHB5, PCDHB6, PCDHB3, PCDHB15, PCDHB2, PCDHB14, CLDN10, PCDHB12, PCDHB11, GPR98, AZGP1, NCAM2, CDH13, PCDHB18, PCDHB16, PCDHB17, CYFIP2, SGCE, EMB, CYR61

25/131 7.1874E-5

25/131 7.3839E-5

GOTERM_BP_FAT GO:0050808 Synapse Organization 1.1247E-7 PCDHB9, MYO6, PCDHB5, PCDHB6, PCDHB3, PCDHB16, PCDHB2, PCDHB14, PCDHB11

PCDHB9, MYO6, PCDHB5, PCDHB6, PCDHB3,

9/131 1.7579E-4

GOTERM_BP_FAT GO:0043062 Extracellular Structure

Organization

3.7672E-6

PCDHB16, PCDHB2, PCDHB14, PCDHB11, APBB2, CYR61

11/131 0.005887

GOTERM_MF_FAT GO:0005509 Calcium Ion Binding 0,001899

GOTERM_BP_FAT GO:0007268 Synaptic Transmission 0,00232676

GOTERM_BP_FAT GO:0019226 Transmission Of Nerve Impulse 0,00665079

PCDHB9, PCDHB7, PCDHB8, PCDHB5, PCDHB6, PCDHB3, PCDHB15, PCDHB2, PCDHB14, PCDHB12, PCDHB11, GPR98, CDH13, CLGN, PCDHB18, PCDHB16, FSTL5, PCDHB17, SGCE

PCDHB9, MYO6, PCDHB5, PCDHB6, PCDHB3, PCDHB16, PCDHB2, APBA2, PCDHB14, PCDHB11

PCDHB9, MYO6, PCDHB5, PCDHB6, PCDHB3, PCDHB16, PCDHB2, APBA2, PCDHB14, PCDHB11

19/121 2.423907

10/131 3.575377

10/131 9.904163

GOTERM_CC_FAT GO:0005886 Plasma Membrane 1.6477E-5

FHIT, ADCY1, GYPE, AP1G2, GPR160, HLA- DMB, HLA-DMA, IL31RA, AZGP1, PCDHB9, PTPRK, F11R, PCDHB7, PCDHB8, MYO6, PCDHB5, MPDZ, PCDHB6, PCDHB3, MFI2, PCDHB2, HLA-DQA2, GPR98, HLA-DQA1, NCAM2, HLA-DPA1, SGCE, TM4SF1, HLA-DRA, HLA-DQB1, COPZ2, HLA-DRB3, APH1B, PCDHB15, PCDHB14, CLDN10, PCDHB12, PCDHB11, GPRC5A, CD74, PCDHB18, PCDHB16, APBA2, HLA-DRB5, HLA-DPB1, HLA- DOA, PLA2R1, HLA-DOB, AXL, EPHA5, CDH13, CYFIP2, CXORF61, SLC14A1, ATP6V0A4

55/112 0.020368

3:4.4351

GOTERM_CC_FAT GO:0016021 Integral To Membrane 4.2283E-5

ADCY1, GYPE, GPR160, HLA-DMB, HLA-DMA, IL31RA, TMEM108, ELOVL6, NALCN, UNC5C, ZDHHC2, PCDHB9, F11R, PTPRK, KIAA1324L, PCDHB7, PCDHB8, PCDHB5, PCDHB6, PCDHB3, MFI2, PCDHB2, HLA-DQA2, HLA-

DQA1, GPR98, TPTE, NCAM2, PLEKHH2, SGCE, HLA-DPA1, TM4SF1, SLITRK6, HLA-DRA, HLA- DQB1, DCC, CYP2U1, TYRP1, IFITM2, HLA- DRB3, PTPLAD2, APH1B, PCDHB15, PCDHB14, CLDN10, PCDHB12, PCDHB11, GPRC5A, CD74, LINGO2, TSC22D3, PCDHB18, PCDHB16, PCDHB17, SLC4A8, HLA-DRB5, ARMCX1, EMB, HLA-DPB1, PLA2R1, HLA-DOA, HLA-DOB, AXL, EPHA5, CLGN, CXORF61, SLC14A1, SLC46A3, ATP6V0A4

68/112 0.052260

GOTERM_CC_FAT GO:0031224 Intrinsic To Membrane 7.1051E-5

ADCY1, GYPE, GPR160, HLA-DMB, HLA-DMA, IL31RA, TMEM108, ELOVL6, NALCN, UNC5C, ZDHHC2, PCDHB9, F11R, PTPRK, KIAA1324L, PCDHB7, PCDHB8, PCDHB5, PCDHB6, PCDHB3, MFI2, PCDHB2, HLA-DQA2, HLA-

DQA1, GPR98, TPTE, NCAM2, PLEKHH2, SGCE, HLA-DPA1, TM4SF1, SLITRK6, HLA-DRA, HLA- DQB1, DCC, CYP2U1, TYRP1, IFITM2, HLA- DRB3, PTPLAD2, APH1B, PCDHB15, PCDHB14, CLDN10, PCDHB12, PCDHB11, GPRC5A, CD74, LINGO2, TSC22D3, PCDHB18, PCDHB16, PCDHB17, SLC4A8, HLA-DRB5, ARMCX1, EMB, HLA-DPB1, PLA2R1, HLA-DOA, HLA-DOB, AXL, EPHA5, CDH13, CLGN, CXORF61, SLC14A1, SLC46A3, ATP6V0A4

69/112 0.0878028

4:3.1758

GOTERM_CC_FAT GO:0044459 Plasma Membrane Part 2.7342E-5

GOTERM_CC_FAT GO:0005887 Integral To Plasma Membrane 0,0028961

GOTERM_CC_FAT GO:0031226 Intrinsic To Plasma Membrane 0,003748

HLA-DQB1, COPZ2, GYPE, HLA-DRB3, PCDHB15, CLDN10, PCDHB12, HLA-DMB, PCDHB11, GPRC5A, HLA-DMA, CD74, AZGP1, HLA-DRB5, HLA-DPB1, PLA2R1, HLA-DOA, HLA- DOB, PTPRK, F11R, MYO6, MPDZ, PCDHB6, PCDHB3, MFI2, AXL, PCDHB2, HLA-DQA2, HLA- DQA1, EPHA5, CDH13, CYFIP2, HLA-DPA1, SGCE, TM4SF1, SLC14A1, ATP6V0A4, HLA-DRA

PTPRK, GYPE, PCDHB6, HLA-DRB3, PCDHB3, MFI2, AXL, PCDHB15, PCDHB2, PCDHB12, PCDHB11, GPRC5A, HLA-DQA2, HLA-DQA1, EPHA5, HLA-DPA1, SGCE, TM4SF1, SLC14A1, PLA2R1, HLA-DRA

PTPRK, GYPE, PCDHB6, HLA-DRB3, PCDHB3, MFI2, AXL, PCDHB15, PCDHB2, PCDHB12, PCDHB11, GPRC5A, HLA-DQA2, HLA-DQA1, EPHA5, HLA-DPA1, SGCE, TM4SF1, SLC14A1, PLA2R1, HLA-DRA

38/112 0.033797

21/112 3522098

21/112 45362816

5:2.255

GOTERM_CC_FAT GO:0042825 TAP Complex 2.1750E-5 HLA-DMB, HLA-DOB, HLA-DMA, HLA-DRA 4/112 0.0268865

MHC Class I Peptide Loading

GOTERM_CC_FAT GO:0042824

Complex 5.1543E-5 HLA-DMB, HLA-DOB, HLA-DMA, HLA-DRA 4/112 0.0637027

GOTERM_MF_FAT GO:0042287 MHC Protein Binding 7.5561E-5 HLA-DMB, HLA-DOB, HLA-DMA, CD74, HLA-DRA 5/121 0.0974871

GOTERM_MF_FAT GO:0042288 MHC Class I Protein Binding 3.9497E-4 HLA-DMB, HLA-DOB, HLA-DMA, HLA-DRA 8/121 0.508613

|  | GOTERM_CC_FAT | GO:0005773 | Vacuole | 0,006364 | CTSZ, GAA, HLA-DMB, ATP6V0A4, HLA-DOB,  HLA-DMA, CD74, HLA-DRA | 8/112 | 7.589919 |
| --- | --- | --- | --- | --- | --- | --- | --- |
|  | GOTERM_CC_FAT | GO:0000323 | Lytic Vacuole | 0,010168 | CTSZ, GAA, HLA-DMB, HLA-DOB, HLA-DMA, CD74, HLA-DRA | 7/112 | 11.869295 |
|  | GOTERM_CC_FAT | GO:0005764 | Lysosome | 0,010168 | CTSZ, GAA, HLA-DMB, HLA-DOB, HLA-DMA, CD74, HLA-DRA | 7/112 | 11.869295 |
|  |  |  |  |  | FHIT, HKR1, ADCY1, ZNF83, ANUBL1, ZNF781, |  |  |
|  |  |  |  |  | SOBP, CLYBL, PTEN, ZFP90, FSTL5, ZNF404, |  |  |
|  |  |  |  |  | NALCN, PCDHB9, ZDHHC2, PCDHB7, PCDHB8, |  |  |
|  |  |  |  |  | PCDHB5, PCDHB6, PCDHB3, MFI2, ZNF790, |  |  |
|  | GOTERM_MF_FAT | GO:0046872 | Metal Ion Binding | 0,003083 | PCDHB2, TRERF1, GPR98, MARK1, LIMCH1, ZSCAN16, NEK9, SGCE, CYP2U1, ZNF808, | 54/121 | 3.908143 |
|  |  |  |  |  | RNF17, TYRP1, NEK3, PCDHB15, PCDHB14, |  |  |
|  |  |  |  |  | PCDHB12, PCDHB11, PCDHB18, ZNF223, |  |  |
|  |  |  |  |  | PCDHB16, PCDHB17, ZNF329, SLC4A8, ZNF607, |  |  |
|  |  |  |  |  | ZNF420, CCNB1IP1, ZNF528, ADARB1, ACSM3, |  |  |
|  |  |  |  |  | CDH13, CLGN, ZNF571, PTENP1 |  |  |
|  |  |  |  |  | FHIT, HKR1, ADCY1, ZNF83, ANUBL1, ZNF781, |  |  |
|  |  |  |  |  | SOBP, CLYBL, PTEN, ZFP90, FSTL5, ZNF404, |  |  |
|  |  |  |  |  | NALCN, PCDHB9, ZDHHC2, PCDHB7, PCDHB8, |  |  |
|  |  |  |  |  | PCDHB5, PCDHB6, PCDHB3, MFI2, ZNF790, |  |  |
| 6:1.5476 | GOTERM_MF_FAT | GO:0043169 | Cation Binding | 0,003839 | PCDHB2, TRERF1, GPR98, MARK1, LIMCH1, ZSCAN16, NEK9, SGCE, CYP2U1, ZNF808, | 54/121 | 4.844576 |
|  |  |  |  |  | RNF17, TYRP1, NEK3, PCDHB15, PCDHB14, |  |  |
|  |  |  |  |  | PCDHB12, PCDHB11, PCDHB18, ZNF223, |  |  |
|  |  |  |  |  | PCDHB16, PCDHB17, ZNF329, SLC4A8, ZNF607, |  |  |
|  |  |  |  |  | ZNF420, CCNB1IP1, ZNF528, ADARB1, ACSM3, |  |  |
|  |  |  |  |  | CDH13, CLGN, ZNF571, PTENP1 |  |  |
|  |  |  |  |  | FHIT, HKR1, ADCY1, ZNF83, ANUBL1, ZNF781, |  |  |
|  |  |  |  |  | SOBP, CLYBL, PTEN, ZFP90, FSTL5, ZNF404, |  |  |
|  |  |  |  |  | NALCN, PCDHB9, ZDHHC2, PCDHB7, PCDHB8, |  |  |
|  |  |  |  |  | PCDHB5, PCDHB6, PCDHB3, MFI2, ZNF790, |  |  |
|  | GOTERM_MF_FAT | GO:0043167 | Ion Binding | 0,005378 | PCDHB2, TRERF1, GPR98, MARK1, LIMCH1, ZSCAN16, NEK9, SGCE, CYP2U1, ZNF808, | 54/121 | 6.724643 |
|  |  |  |  |  | RNF17, TYRP1, NEK3, PCDHB15, PCDHB14, |  |  |
|  |  |  |  |  | PCDHB12, PCDHB11, PCDHB18, ZNF223, |  |  |
|  |  |  |  |  | PCDHB16, PCDHB17, ZNF329, SLC4A8, ZNF607, |  |  |
|  |  |  |  |  | ZNF420, CCNB1IP1, ZNF528, ADARB1, ACSM3, |  |  |
|  |  |  |  |  | CDH13, CLGN, ZNF571, PTENP1 |  |  |

GOTERM_BP_FAT GO:0030030 Cell Projection Organization 0,002901

DCC, NCAM2, CDH13, MYO6, DLX5, KIF5C, UNC5C, APBB2, SLITRK6, PTEN, GPR98, PTENP1

11/131 4.44054

7:1.5031

GOTERM_BP_FAT GO:0031175 Neuron Projection Development 0,003292 DCC, NCAM2, MYO6, DLX5, KIF5C, UNC5C, APBB2, SLITRK6, PTEN, PTENP1

GOTERM_BP_FAT GO:0048666 Neuron Development 0,005428 DCC, NCAM2, MYO6, DLX5, KIF5C, UNC5C, APBB2, SLITRK6, PTEN, GPR98, PTENP1

GOTERM_BP_FAT GO:0007409 Axonogenesis 0,010817 DCC, NCAM2, DLX5, KIF5C, UNC5C, APBB2, SLITRK6

9/131 5.023613

10/131 8.156321

7/131 15.63332

GOTERM_BP_FAT GO:0002495

Antigen Processing And Presentation Of Peptide Antigen Via MHC Class II

4.6880E-5 HLA-DOA, HLA-DMA, CD74, HLA-DRA 4/131 0.0732471

GOTERM_BP_FAT GO:0019886

Antigen Processing And Presentation Of Exogenous Peptide Antigen Via MHC Class II

4.6880E-5 HLA-DOA, HLA-DMA, CD74, HLA-DRA 4/131 0.073247

GOTERM_MF_FAT GO:0042287 MHC Protein Binding 7.5561E-5 HLA-DMB, HLA-DOB, HLA-DMA, CD74, HLA-DRA 5/121 0.097487

Antigen Processing And

8:1.3184

GOTERM_BP_FAT GO:0002478

GOTERM_BP_FAT GO:0019884

Presentation Of Exogenous

Peptide Antigen

Antigen Processing And Presentation Of Exogenous Antigen

1.3523E-4 HLA-DOA, HLA-DMA, CD74, HLA-DRA 4/131 0.211164

2.9211E-4 HLA-DOA, HLA-DMA, CD74, HLA-DRA 4/131 0.455582

GOTERM_BP_FAT GO:0048002 Antigen Processing And

Presentation Of Peptide Antigen

GOTERM_BP_FAT GO:0045619 Regulation Of Lymphocyte

Differentiation

0,0023830 HLA-DOA, HLA-DMA, CD74, HLA-DRA 4/131 3.660411

0,00313023 INHBA, IL7, HLA-DOA, HLA-DMA, CD74 5/131 4.7819766

GOTERM_CC_FAT GO:0005773 Vacuole 0,00636453 CTSZ, GAA, HLA-DMB, ATP6V0A4, HLA-DOB, HLA-DMA, CD74, HLA-DRA

GOTERM_CC_FAT GO:0000323 Lytic Vacuole 0,01016816 CTSZ, GAA, HLA-DMB, HLA-DOB, HLA-DMA, CD74, HLA-DRA

GOTERM_CC_FAT GO:0005764 Lysosome 0,01016816 CTSZ, GAA, HLA-DMB, HLA-DOB, HLA-DMA, CD74, HLA-DRA

8/112 7.589919

7/112 11.86929

7/112 11.86929

**Upregulated G10 vs A7**

GOTERM_CC_FAT GO:0042613 MHC Class II Protein Complex 4.8697E-18

HLA-DQB1, HLA-DRB3, HLA-DRB5, HLA-DPA1, HLA-DPB1, HLA-DMB, HLA-DOA, HLA-DQA2, HLA-DOB, HLA-DMA, HLA-DQA1, HLA-DRA

12/77 5.886E-15

KEGG_PATHWAY hsa05310 Asthma 1.8511E-17

Antigen Processing And

HLA-DQB1, HLA-DRB3, HLA-DRB5, HLA-DPA1, HLA-DPB1, HLA-DMB, HLA-DOA, HLA-DQA2, HLA-DOB, HLA-DMA, HLA-DQA1, HLA-DRA

HLA-DQB1, HLA-DRB3, HLA-DMB, HLA-DMA,

12/38 1.758E-14

GOTERM_BP_FAT GO:0002504

Presentation Of Peptide Or Polysaccharide Antigen Via MHC Class II

2.3871E-17

HLA-DQA2, HLA-DQA1, CD74, HLA-DRB5, HLA- DPA1, HLA-DPB1, HLA-DOA, HLA-DOB, HLA- DRA

13/116 3.745E-14

KEGG_PATHWAY hsa05330 Allograft Rejection 3.1005E-16

HLA-DQB1, HLA-DRB3, HLA-DRB5, HLA-DPA1, HLA-DPB1, HLA-DMB, HLA-DOA, HLA-DQA2, HLA-DOB, HLA-DMA, HLA-DQA1, HLA-DRA

12/38 3.108E-13

KEGG_PATHWAY hsa05332 Graft-Versus-Host Disease 8.5166E-16

HLA-DQB1, HLA-DRB3, HLA-DRB5, HLA-DPA1, HLA-DPB1, HLA-DMB, HLA-DOA, HLA-DQA2, HLA-DOB, HLA-DMA, HLA-DQA1, HLA-DRA

12/38 8.437E-13

KEGG_PATHWAY hsa04940 Type I Diabetes Mellitus 2.1416E-15

HLA-DQB1, HLA-DRB3, HLA-DRB5, HLA-DPA1, HLA-DPB1, HLA-DMB, HLA-DOA, HLA-DQA2, HLA-DOB, HLA-DMA, HLA-DQA1, HLA-DRA

12/38 1.998E-12

GOTERM_MF_FAT GO:0032395 MHC Class II Receptor Activity 1.1731E-14

HLA-DQB1, HLA-DRB3, HLA-DPA1, HLA-DPB1, HLA-DOA, HLA-DQA2, HLA-DOB, HLA-DMA, HLA- DQA1, HLA-DRA

10/109 1.507E-11

KEGG_PATHWAY hsa04672 Intestinal Immune Network For

1.4280E-14

IgA Production

HLA-DQB1, HLA-DRB3, HLA-DRB5, HLA-DPA1, HLA-DPB1, HLA-DMB, HLA-DOA, HLA-DQA2, HLA-DOB, HLA-DMA, HLA-DQA1, HLA-DRA

12/38 1.360E-11

1:12.2765

GOTERM_CC_FAT GO:0042611 MHC Protein Complex 2.2948E-14

KEGG_PATHWAY hsa05320 Autoimmune Thyroid Disease 2.3188E-14

HLA-DQB1, HLA-DRB3, HLA-DRB5, HLA-DPA1, HLA-DPB1, HLA-DMB, HLA-DOA, HLA-DQA2, HLA-DOB, HLA-DMA, HLA-DQA1, HLA-DRA

HLA-DQB1, HLA-DRB3, HLA-DRB5, HLA-DPA1, HLA-DPB1, HLA-DMB, HLA-DOA, HLA-DQA2, HLA-DOB, HLA-DMA, HLA-DQA1, HLA-DRA

12/77 2.777E-11

12/38 2.203E-11

KEGG_PATHWAY hsa04612 Antigen Processing And

2.1039E-13

Presentation

HLA-DQB1, HLA-DRB3, HLA-DMB, HLA-DMA, HLA-DQA2, HLA-DQA1, CD74, HLA-DRB5, HLA- DPA1, HLA-DPB1, HLA-DOA, HLA-DOB, HLA- DRA

13/38 1.998E-10

KEGG_PATHWAY hsa05416 Viral Myocarditis 1.1317E-12

HLA-DQB1, HLA-DRB3, HLA-DRB5, HLA-DPA1, HLA-DPB1, HLA-DMB, HLA-DOA, HLA-DQA2, HLA-DOB, HLA-DMA, HLA-DQA1, HLA-DRA

12/38 1.0749E-9

KEGG_PATHWAY hsa04514 Cell Adhesion Molecules (CAMs) 2.8130E-12

HLA-DQB1, HLA-DRB3, HLA-DMB, HLA-DMA, HLA-DQA2, HLA-DQA1, NRCAM, NCAM2, HLA- DRB5, HLA-DPA1, HLA-DPB1, HLA-DOA, HLA- DOB, HLA-DRA

HLA-DQB1, HLA-DRB3, HLA-DMB, HLA-DMA,

14/38 2.6719E-9

GOTERM_BP_FAT GO:0019882 Antigen Processing And

Presentation

4.6336E-12

HLA-DQA2, HLA-DQA1, CD74, HLA-DRB5, HLA- DPA1, HLA-DPB1, HLA-DOA, HLA-DOB, HLA- DRA

13/116 7.2703E-9

KEGG_PATHWAY hsa05322 Systemic Lupus Erythematosus 4.8758E-11

HLA-DQB1, HLA-DRB3, HLA-DRB5, HLA-DPA1, HLA-DPB1, HLA-DMB, HLA-DOA, HLA-DQA2, HLA-DOB, HLA-DMA, HLA-DQA1, HLA-DRA

12/38 4.6312E-8

REACTOME_PATH

WAY REACT_6900 Signaling In Immune System 9.6269E-8

GOTERM_BP_FAT GO:0006955 Immune Response 7.3291E-4

UP_TISSUE Blood 0,00910

GOTERM_CC_FAT GO:0044459 Plasma Membrane Part 5.0391E-9

HLA-DQB1, HLA-DRB3, HLA-DMB, PTEN, HLA- DMA, HLA-DQA2, HLA-DQA1, HLA-DRB5, RIPK2, HLA-DPA1, HLA-DPB1, HLA-DOA, HLA-DOB, PTENP1, HLA-DRA

HLA-DQB1, HLA-DRB3, HLA-DMB, HLA-DMA, HLA-DQA2, HLA-DQA1, CD74, IL31RA, HLA- DRB5, SEMA3C, HLA-DPA1, HLA-DPB1, ODZ1, HLA-DOA, HLA-DOB, HLA-DRA

HLA-DQB1, CTSZ, GYPE, HLA-DRB3, HLA- DRB5, HLA-DPB1, HLA-DMB, SLC14A1, HLA- DQA2, EMP1, HLA-DQA1, HLA-DRA

HLA-DQB1, SLC5A3, COPZ2, GYPE, HLA-DRB3, LPAR4, HLA-DMB, GPRC5A, HLA-DMA, CD74, STARD13, NRCAM, FAT1, HLA-DRB5, HLA- DPB1, PLA2R1, ODZ1, HLA-DOA, HLA-DOB, FLRT3, MYO6, MPDZ, PCDHB3, MFI2, AXL, HLA-

DQA2, HLA-DQA1, P2RX5, CDH13, CHRM3, HLA- DPA1, SGCE, TM4SF1, SLC14A1, DST, HLA-

DRA

14/29 6.9703E-5

16/116 1.143776

12/133 9.701379

36/77 6.0911E-6

|  | | | | HLA-DQB1, SLC5A3, COPZ2, GYPE, AP1G2, |  | |
| --- | --- | --- | --- | --- | --- | --- |
|  |  |  |  | HLA-DRB3, APH1B, LPAR4, HLA-DMB, GPRC5A, |  |  |
|  |  |  |  | HLA-DMA, CD74, STARD13, IL31RA, NRCAM, |  |  |
| GOTERM_CC_FAT | GO:0005886 | Plasma Membrane | 2.4417E-6 | FAT1, HLA-DRB5, HLA-DPB1, PLA2R1, HLA- DOA, ODZ1, HLA-DOB, FLRT3, MYO6, MPDZ, | 43/77 | 0.002951 |
|  | | | | PCDHB3, MFI2, AXL, HLA-DQA2, FLNA, HLA- |  |  |
|  |  |  |  | DQA1, P2RX5, NCAM2, CDH13, CHRM3, |  |  |
|  |  |  |  | CXORF61, HLA-DPA1, SGCE, TM4SF1, |  |  |
|  |  |  |  | SLC14A1, DST, EMP1, HLA-DRA |  |  |

2:5.062

GOTERM_CC_FAT GO:0005887 Integral To Plasma Membrane 3.5784E-6

GOTERM_CC_FAT GO:0031226 Intrinsic To Plasma Membrane 5.1066E-6

FLRT3, SLC5A3, GYPE, HLA-DRB3, PCDHB3, MFI2, LPAR4, AXL, GPRC5A, HLA-DQA2, HLA- DQA1, NRCAM, P2RX5, CHRM3, FAT1, HLA- DPA1, SGCE, TM4SF1, SLC14A1, PLA2R1, ODZ1, HLA-DRA

FLRT3, SLC5A3, GYPE, HLA-DRB3, PCDHB3, MFI2, LPAR4, AXL, GPRC5A, HLA-DQA2, HLA- DQA1, NRCAM, P2RX5, CHRM3, FAT1, HLA- DPA1, SGCE, TM4SF1, SLC14A1, PLA2R1, ODZ1, HLA-DRA

22/77 0.004325

22/77 0.0061726

GOTERM_CC_FAT GO:0016021 Integral To Membrane 0,00130329

HLA-DQB1, SLC5A3, DCC, GYPE, HLA-DRB3, PTPLAD2, APH1B, LPAR4, HLA-DMB, GPRC5A, HLA-DMA, CD74, IL31RA, NRCAM, TMEM108, FAT1, SLC4A8, HLA-DRB5, NALCN, UNC5C, HLA- DPB1, PLA2R1, HLA-DOA, SLCO5A1, ODZ1,

HLA-DOB, FLRT3, PCDHB3, MFI2, MXRA7, AXL, EDA2R, HLA-DQA2, HLA-DQA1, P2RX5, NCAM2, CHRM3, PLEKHH2, CXORF61, HLA-DPA1,

SGCE, TM4SF1, SLC14A1, SLITRK6, EMP1, HLA- DRA

46/77 1.564049

GOTERM_CC_FAT GO:0031224 Intrinsic To Membrane 0,00144887

HLA-DQB1, SLC5A3, DCC, GYPE, HLA-DRB3, PTPLAD2, APH1B, LPAR4, HLA-DMB, GPRC5A, HLA-DMA, CD74, IL31RA, NRCAM, TMEM108, FAT1, SLC4A8, HLA-DRB5, NALCN, UNC5C, HLA DPB1, PLA2R1, HLA-DOA, SLCO5A1, ODZ1,

HLA-DOB, FLRT3, PCDHB3, MFI2, MXRA7, AXL, EDA2R, HLA-DQA2, HLA-DQA1, P2RX5, NCAM2, CDH13, CHRM3, PLEKHH2, CXORF61, HLA- DPA1, SGCE, TM4SF1, SLC14A1, SLITRK6, EMP1, HLA-DRA

47/77 1.737360

GOTERM_BP_FAT GO:0006355 Regulation Of Transcription, DNA- Dependent

2.6116E-6

ZNF808, ZNF85, ZNF582, ZNF83, ZNF583, ZNF558, HDX, HOXD10, IL31RA, ZNF709, ZFP90, ZNF737, ZNF404, ZNF286A, MEIS3P1, RUNX1, ZNF420, ZNF607, ZNF724P, ALX1, ZNF528, MYO6, ZNF542, ZNF284, ZFP30, ZNF790, ZNF221, ZFP28, INHBA, ZNF197, TGIF1, TFAP2A, ZSCAN16, ZNF571, ZNF573

35/116 0.004097

GOTERM_BP_FAT GO:0045449 Regulation Of Transcription 3.9151E-6

ZNF808, ZNF85, ZNF582, ZNF83, ZNF583, ZNF558, ZNF781, HDX, HOXD10, IL31RA, ZNF709, ZFP90, ZNF737, ZNF404, ZNF329, ZNF286A, MEIS3P1, ZNF607, RUNX1, ZNF420, ZNF724P, ALX1, ZNF528, KHDRBS3, MYO6, ZNF542, ZNF284, SNAPC1, ZFP30, ZNF790, EDA2R, ZNF521, ZNF221, ZFP28, FLNA, INHBA, ZNF197, BTG1, TGIF1, RIPK2, TFAP2A, ZSCAN16, ZNF571, ZNF573

44/116 0.0061427

3:3.4348

GOTERM_BP_FAT GO:0051252 Regulation Of RNA Metabolic

4.3136E-6

Process

ZNF808, ZNF85, ZNF582, ZNF83, ZNF583, ZNF558, HDX, HOXD10, IL31RA, ZNF709, ZFP90, ZNF737, ZNF404, ZNF286A, MEIS3P1, RUNX1, ZNF420, ZNF607, ZNF724P, ALX1, ZNF528, MYO6, ZNF542, ZNF284, ZFP30, ZNF790, ZNF221, ZFP28, INHBA, ZNF197, TGIF1, TFAP2A, ZSCAN16, ZNF571, ZNF573

35/116 0.006767

GOTERM_MF_FAT GO:0003677 DNA Binding 1.9807E-4

GOTERM_BP_FAT GO:0006350 Transcription 5.2216E-4

GOTERM_MF_FAT GO:0008270 Zinc Ion Binding 8.2350E-4

ZNF808, ZNF85, ZNF582, ZNF83, ZNF583, ZNF558, ZNF781, HDX, HOXD10, ZNF709, ZFP90, ZNF737, ZNF404, ZNF329, ZNF286A, MEIS3P1, RUNX1, ZNF420, ZNF607, ZNF724P, ALX1, ZNF528, ZNF542, ZNF284, SNAPC1, ZFP30, ZNF790, ZNF521, ZNF221, ZFP28, ZNF197, TGIF1, TFAP2A, ZSCAN16, ZNF571, ZNF573

ZNF808, ZNF85, ZNF582, ZNF83, ZNF583, ZNF558, ZNF781, HOXD10, ZNF709, ZFP90, ZNF404, ZNF329, ZNF286A, RUNX1, ZNF420, ZNF607, ALX1, ZNF528, KHDRBS3, ZNF542, ZNF284, SNAPC1, ZFP30, ZNF790, ZNF521, ZNF221, ZFP28, ZNF197, TGIF1, TFAP2A, ZSCAN16, ZNF571, ZNF573

ZNF808, ZNF85, ZNF582, ZNF83, RNF17, ZNF583, ZNF558, ZNF781, SOBP, ZNF709, ZFP90, ZNF737, ZNF404, ZNF329, ZNF286A, ZNF420, ZNF607, ZNF724P, DCTD, ZNF528, ADARB1, ZNF542, ZNF284, ZFP30, MFI2, ZNF790, ZNF521, ZNF221, ZFP28, ZNF197, CA8, ZSCAN16, ZNF571, ZNF573

36/109 0.253473

33/116 0.816149

34/109 1.049918

GOTERM_CC_FAT GO:0042825 TAP complex 6.9506E-6 HLA-DMB, HLA-DOB, HLA-DMA, HLA-DRA 4/77 0.008401

MHC class I peptide loading

GOTERM_CC_FAT GO:0042824

complex 1.6539E-5 HLA-DMB, HLA-DOB, HLA-DMA, HLA-DRA 4/77 0.019990

|  | GOTERM_BP_FAT | GO:0002495 | Antigen Processing And  Presentation of peptide antigen | 3.2489E-5 | HLA-DOA, HLA-DMA, CD74, HLA-DRA | 4/116 | 0.050964 |
| --- | --- | --- | --- | --- | --- | --- | --- |
|  |  |  | via MHC class II |  |  |  |  |
|  |  |  | Antigen Processing And |  |  |  |  |
|  | GOTERM_BP_FAT | GO:0019886 | Presentation Of Exogenous | 3.2489E-5 | HLA-DOA, HLA-DMA, CD74, HLA-DRA | 4/116 | 0.050964 |
|  |  |  | Peptide Antigen Via MHC Class II |  |  |  |  |
|  | GOTERM_MF_FAT | GO:0042287 | MHC Protein Binding | 5.0068E-5 | HLA-DMB, HLA-DOB, HLA-DMA, CD74, HLA-DRA | 5/109 | 0.064126 |
| 4:2.0666 | GOTERM_BP_FAT | GO:0002478 | Antigen Processing And  Presentation Of Exogenous | 9.3959E-5 | HLA-DOA, HLA-DMA, CD74, HLA-DRA | 4/116 | 0.147321 |
|  |  |  | Peptide Antigen |  |  |  |  |
|  |  |  | Antigen Processing And |  |  |  |  |
|  | GOTERM_BP_FAT | GO:0019884 | Presentation Of Exogenous | 2.0345E-4 | HLA-DOA, HLA-DMA, CD74, HLA-DRA | 4/116 | 0.318745 |
|  |  |  | Peptide Antigen |  |  |  |  |
|  | GOTERM_MF_FAT | GO:0042288 | MHC Class I Protein Binding | 2.8972E-4 | HLA-DMB, HLA-DOB, HLA-DMA, HLA-DRA | 4/109 | 0.370550 |
|  | GOTERM_BP_FAT | GO:0048002 | Antigen Processing And  Presentation of peptide antigen | 0,00167908 | HLA-DOA, HLA-DMA, CD74, HLA-DRA | 4/116 | 2.602252 |
|  | GOTERM_CC_FAT | GO:0000323 | Lytic Vacuole | 0,00836888 | CTSZ, HLA-DMB, HLA-DOB, HLA-DMA, CD74, HLA-DRA | 6/77 | 9.659692 |
|  | GOTERM_CC_FAT | GO:0005764 | Lysosome | 0,00836888 | CTSZ, HLA-DMB, HLA-DOB, HLA-DMA, CD74, HLA-DRA | 6/77 | 9.659692 |
|  | GOTERM_BP_FAT | GO:0030030 | Cell Projection Organization | 2.9488E-4 | DCC, DNM3, MYO6, KIF5C, PTEN, NRCAM, CDH13, NCAM2, CAPG, UNC5C, SLITRK6, DST, PTENP1 | 12/116 | 0.461677 |
|  | GOTERM_CC_FAT | GO:0043005 | Neuron Projection | 9.3660E-4 | NRCAM, DCC, DNM3, NCAM2, CDH13, MYO6, CHRM3, MPDZ, KIF5C | 9/77 | 1.126277 |
|  | GOTERM_BP_FAT | GO:0031175 | Neuron Projection Development | 0,00152647 | NRCAM, DCC, NCAM2, MYO6, KIF5C, UNC5C, SLITRK6, DST, PTEN, PTENP1 | 9/116 | 2.368365 |
| 5:1.8441 | GOTERM_BP_FAT | GO:0048666 | Neuron Development | 0,00240002 | NRCAM, DCC, NCAM2, MYO6, KIF5C, UNC5C, SLITRK6, DST, PTEN, HOXD10, PTENP1 | 10/116 | 3.699980 |
|  | GOTERM_BP_FAT | GO:0007409 | Axonogenesis | 0,00607524 | NRCAM, DCC, NCAM2, KIF5C, UNC5C, SLITRK6, DST | 7/116 | 9.118297 |
|  | GOTERM_BP_FAT | GO:0048667 | Cell Morphogenesis Involved In  Neuron Differentiation | 0,00884172 | NRCAM, DCC, NCAM2, KIF5C, UNC5C, SLITRK6, DST | 7/116 | 13.007111 |
|  | GOTERM_BP_FAT | GO:0048812 | Neuron Projection Morphogenesis | 0,00965496 | NRCAM, DCC, NCAM2, KIF5C, UNC5C, SLITRK6, DST | 7/116 | 14.120289 |
| 6:1.4812 | GOTERM_BP_FAT | GO:0007416 | Synaptogenesis | 0,00248024 | NRCAM, DNM3, MYO6, PCDHB3 | 4/116 | 3.8214112 |

| GOTERM_BP_FAT | GO:0002495 | Antigen Processing And  Presentation Of Peptide Antigen | 3.2489E-5 | HLA-DOA, HLA-DMA, CD74, HLA-DRA | 4/116 | 0.0509643 |
| --- | --- | --- | --- | --- | --- | --- |
|  |  | Via MHC Class II |  |  |  |  |
|  |  | Antigen Processing And |  |  |  |  |
| GOTERM_BP_FAT | GO:0019886 | Presentation Of Exogenous | 3.2489E-5 | HLA-DOA, HLA-DMA, CD74, HLA-DRA | 4/116 | 0.0509643 |
|  |  | Peptide Antigen Via MHC Class II |  |  |  |  |
|  |  | Antigen Processing And |  |  |  |  |
| GOTERM_BP_FAT | GO:0002478 | Presentation Of Exogenous | 9.3959E-5 | HLA-DOA, HLA-DMA, CD74, HLA-DRA | 4/116 | 0.1473216 |
| 7:1.4547 |  | Peptide Antigen |  |  |  |  |
|  |  | Antigen Processing And |  |  |  |  |
| GOTERM_BP_FAT | GO:0019884 | Presentation Of Exogenous | 2.0345E-4 | HLA-DOA, HLA-DMA, CD74, HLA-DRA | 4/116 | 0.3187454 |
|  |  | Antigen |  |  |  |  |
| GOTERM_BP_FAT | GO:0048002 | Antigen Processing And  Presentation Of Peptide Antigen | 0,00167909 | HLA-DOA, HLA-DMA, CD74, HLA-DRA | 4/116 | 2.602252 |
| **Downregulated G10 vs Control** |  |  |  |  |  |  |
|  |  |  |  | **MTSS1**, PDGFA, FSCN1, CSPG4, TAC1, RDX,  SDC3, NCAM1, PROM1, CTTNBP2, SEMA6A,  SLC1A3, TIAM2, BACE1, NEFH, ROBO2, LRP2,  CACNA1C |  |  |
| GOTERM_CC_FAT | GO:0042995 | Cell Projection | 3,5968E-4 |  | 18/124 | 0.452416 |
| 2:2.6823 |  |  |  |  |  |  |
| GOTERM_CC_FAT | GO:0030424 | Axon | 0,00434 | NCAM1, SEMA6A, BACE1, NEFH, TAC1,  ROBO2, SDC3 | 7/124 | 5.329076 |
| GOTERM_CC_FAT | GO:0043005 | Neuron Projection | 0,00575 | NCAM1, SEMA6A, SLC1A3, TIAM2, BACE1, NEFH, TAC1, ROBO2, CACNA1C, SDC3 | 10/124 | 7.0147288 |
| GOTERM_BP_FAT | GO:0000904 | Cell Morphogenesis Involved In  Differentiation | 0,00387 | SLITRK2, SEMA5A, SLITRK1, SEMA6A, SLC1A3, EFNA5, ROBO2, EPHB2, FN1 | 9/141 | 6.075256 |
| GOTERM_BP_FAT  4:1.7671 | GO:0048667 | Cell Morphogenesis Involved In  Neuron Differentiation | 0,00605 | SLITRK2, SEMA5A, SLITRK1, SEMA6A, SLC1A3, EFNA5, ROBO2, EPHB2 | 8/141 | 9.345023 |
| KEGG_PATHWAY | hsa04360 | Axon Guidance | 0,00668 | SEMA5A, SEMA6A, PLXNA2, EFNB2, EFNA5,  ROBO2, EPHB2 | 7/68 | 7.042549 |
| GOTERM_BP_FAT | GO:0048666 | Neuron Development | 0,00870 | SLITRK2, LIF, SEMA5A, SLITRK1, SEMA6A, SLC1A3, EFNA5, ROBO2, LHX8, EPHB2 | 10/14 | 13.174477 |
| 5:1.5411 KEGG_PATHWAY | hsa04810 | Regulation Of Actin Cytoskeleton | 0,00184 | TIAM2, **TIAM1**, PDGFA, DIAPH3, ITGA11, IQGAP2, ITGA10, TMSB4X, RDX, FN1 | 10/68 | 1.9905308 |
| 6:1.399 GOTERM_BP_FAT | GO:0006928 | Cell Motion | 0,00382 | **MTSS1**, PLXNA2, ITGA11, KITLG, EPHB2, SEMA5A, SEMA6A, CTTNBP2, ROBO2, EFNA5, PPAP2B, TWIST1, FN1 | 13/141 | 6.006306 |
| 19:0.8053 GOTERM_MF_FAT | GO:0003779 | Actin Binding | 0,00667 | CORO2B, DIXDC1, **MTSS1**, MYO10, DIAPH3, ENC1, FSCN1, IQGAP2, TMSB4X, RDX | 10/135 | 8.724749 |

**Downregulated G10 vs A7**

GOTERM_CC_FAT GO:0042995 Cell Projection 2.7223E-4

1:2.5497

**MTSS1**, TRPM6, SWAP70, FSCN1, **UCHL1**, CSPG4, RDX, APBB1IP, SDC3, NCAM1, PROM1, PCSK1, SEMA6A, CTTN, SLC1A3, ROBO2, SLC38A1, LRP2, MERTK, THEM4, APBB1, NEFL, LCP1, NMU

24/191 0.360143

GOTERM_CC_FAT GO:0030424 Axon 0,00258579 NCAM1, SEMA6A, PCSK1, **UCHL1**, ROBO2, SLC38A1, NEFL, NMU, SDC3

9/191 3.3727559

6:1.5123

GOTERM_BP_FAT GO:0000904 Cell Morphogenesis Involved In

0,0030477

Differentiation

GOTERM_BP_FAT GO:0048667 Cell Morphogenesis Involved In

0,00355102

Neuron Differentiation

SLITRK2, SEMA5A, SLITRK1, SEMA6A, SLC1A3,

**UCHL1**, EFNA5, ROBO2, NEFL, APBB1, FN1 11/198 4.922540

SLITRK2, SEMA5A, SLITRK1, SEMA6A, SLC1A3,

**UCHL1**, EFNA5, ROBO2, NEFL, APBB1 10/198 5.713249

GOTERM_BP_FAT GO:0007409 Axonogenesis 0,00736447 SLITRK2, SEMA5A, SLITRK1, SEMA6A, **UCHL1**, EFNA5, ROBO2, NEFL, APBB1

9/198 11.50646

a Complete gene titles within each process are detailed in Table S2
