# Supplementary material for: Reprogramming human A375 amelanotic melanoma cells by catalase overexpression: Upregulation of antioxidant genes correlates with regression of melanoma malignancy and with malignant progression when downregulated
Source: Oncotarget. 2016 May 10;7(27):41154–71. doi: 10.18632/oncotarget.9273 (PMC5173049; doi:10.18632/oncotarget.9273)
Supplement: Supplementary file 3 [file oncotarget-07-41154-s003.docx]

**Table S2.** Significant processes obtained after DAVID analysis with their corresponding gene symbols and gene titles.

**Gene Symbol Gene Title**

**Upregulated A7 vs Control**

GO:0007155 **Cell Adhesion**

LAMA2 Laminin, Alpha 2

CDH7 Cadherin 7, Type 2

LAMB3 Laminin, Beta 3

PCDHB8 Protocadherin Beta 8

PCDHB5 Protocadherin Beta 5

MAGI1 Membrane Associated Guanylate Kinase, WW And PDZ Domain Containing 1

CNTN1 Contactin 1

CNTN3 Contactin 3 (Plasmacytoma Associated)

ENTPD1 Ectonucleoside Triphosphate Diphosphohydrolase 1

MEGF10 Multiple EGF-Like-Domains 10

SYK Spleen Tyrosine Kinase

GO:0022610 **Biological Adhesion**

LAMA2 Laminin, Alpha 2

CDH7 Cadherin 7, Type 2

LAMB3 Laminin, Beta 3

PCDHB8 Protocadherin Beta 8

PCDHB5 Protocadherin Beta 5

MAGI1 Membrane Associated Guanylate Kinase, WW And PDZ Domain Containing 1

CNTN1 Contactin 1

CNTN3 Contactin 3 (Plasmacytoma Associated)

ENTPD1

Ectonucleoside Triphosphate Diphosphohydrolase 1

MEGF10 Multiple EGF-Like-Domains 10

SYK Spleen Tyrosine Kinase

GO:0005604 **Basement Membrane**

LAMA2 Laminin, Alpha 2

LAMB3 Laminin, Beta 3

CCDC80 Coiled-Coil Domain Containing 80

ENTPD1 Ectonucleoside Triphosphate Diphosphohydrolase 1

GO:0007267 **Cell-Cell Signaling**

PCSK1 Proprotein Convertase Subtilisin/Kexin Type 1

SLC17A6

Solute Carrier Family 17 (Vesicular Glutamate Transporter), Member 6

PCDHB5 Protocadherin Beta 5

IL7 Interleukin 7

FGF14 Fibroblast Growth Factor 14

MAOA Monoamine Oxidase A

MME Membrane Metallo-Endopeptidase

CHRNA6 Cholinergic Receptor, Nicotinic, Alpha 6 (Neuronal) AMPH Amphiphysin

SYK Spleen Tyrosine Kinase

**Upregulated G10 vs Control**

| hsa05310 | **Asthma** |  |
| --- | --- | --- |
|  | HLA-DQB1  HLA-DRB3  HLA-DRB5 | Major Histocompatibility Complex, Class II, DQ Beta 1  Major Histocompatibility Complex, Class II, DR Beta 3  Major Histocompatibility Complex, Class II, DR Beta 5 |

HLA-DPA1 Major Histocompatibility Complex, Class II, DP Alpha 1

HLA-DPB1 Major Histocompatibility Complex, Class II, DP Beta 1

HLA-DMB Major Histocompatibility Complex, Class II, DM Beta HLA-DOA Major Histocompatibility Complex, Class II, DO Alpha HLA-DQA2 Major Histocompatibility Complex, Class II, DQ Alpha 2

HLA-DOB Major Histocompatibility Complex, Class II, DO Beta

HLA-DMA Major Histocompatibility Complex, Class II, DM Alpha

HLA-DQA1 Major Histocompatibility Complex, Class II, DQ Alpha 1

GO:0002504

**Antigen Processing And Presentation Of Peptide Or Polysaccharide Antigen Via MHC Class II**

HLA-DQB1 Major Histocompatibility Complex, Class II, DQ Beta 1

HLA-DRB3 Major Histocompatibility Complex, Class II, DR Beta 3

HLA-DMB Major Histocompatibility Complex, Class II, DM Beta HLA-DMA Major Histocompatibility Complex, Class II, DM Alpha HLA-DQA2 Major Histocompatibility Complex, Class II, DQ Alpha 2

HLA-DQA1 Major Histocompatibility Complex, Class II, DQ Alpha 1

CD74 CD74 Molecule, Major Histocompatibility Complex, Class II Invariant Chain

HLA-DRB5 Major Histocompatibility Complex, Class II, DR Beta 5

HLA-DPA1 Major Histocompatibility Complex, Class II, DP Alpha 1

HLA-DPB1 Major Histocompatibility Complex, Class II, DP Beta 1

HLA-DOA Major Histocompatibility Complex, Class II, DO Alpha HLA-DOB Major Histocompatibility Complex, Class II, DO Beta HLA-DRA Major Histocompatibility Complex, Class II, DR Alpha

GO:0042613 **MHC Class II Protein Complex**

HLA-DQB1 Major Histocompatibility Complex, Class II, DQ Beta 1

HLA-DRB3 Major Histocompatibility Complex, Class II, DR Beta 3

HLA-DRB5 Major Histocompatibility Complex, Class II, DR Beta 5

HLA-DPA1 Major Histocompatibility Complex, Class II, DP Alpha 1

HLA-DPB1 Major Histocompatibility Complex, Class II, DP Beta 1

HLA-DMB Major Histocompatibility Complex, Class II, DM Beta HLA-DOA Major Histocompatibility Complex, Class II, DO Alpha HLA-DQA2 Major Histocompatibility Complex, Class II, DQ Alpha 2

HLA-DOB Major Histocompatibility Complex, Class II, DO Beta HLA-DMA Major Histocompatibility Complex, Class II, DM Alpha HLA-DQA1 Major Histocompatibility Complex, Class II, DQ Alpha 1

HLA-DRA Major Histocompatibility Complex, Class II, DR Alpha hsa05330 **Allograft Rejection**

HLA-DQB1 Major Histocompatibility Complex, Class II, DQ Beta 1

HLA-DRB3 Major Histocompatibility Complex, Class II, DR Beta 3

HLA-DRB5 Major Histocompatibility Complex, Class II, DR Beta 5

HLA-DPA1 Major Histocompatibility Complex, Class II, DP Alpha 1

HLA-DPB1 Major Histocompatibility Complex, Class II, DP Beta 1

HLA-DMB Major Histocompatibility Complex, Class II, DM Beta HLA-DOA Major Histocompatibility Complex, Class II, DO Alpha HLA-DQA2 Major Histocompatibility Complex, Class II, DQ Alpha 2

HLA-DOB Major Histocompatibility Complex, Class II, DO Beta HLA-DMA Major Histocompatibility Complex, Class II, DM Alpha HLA-DQA1 Major Histocompatibility Complex, Class II, DQ Alpha 1

HLA-DRA Major Histocompatibility Complex, Class II, DR Alpha hsa05332 **Graft-Versus-Host Disease**

HLA-DQB1 Major Histocompatibility Complex, Class II, DQ Beta 1

HLA-DRB3 Major Histocompatibility Complex, Class II, DR Beta 3

HLA-DRB5 Major Histocompatibility Complex, Class II, DR Beta 5

HLA-DPA1 Major Histocompatibility Complex, Class II, DP Alpha 1

HLA-DPB1 Major Histocompatibility Complex, Class II, DP Beta 1

HLA-DMB Major Histocompatibility Complex, Class II, DM Beta HLA-DOA Major Histocompatibility Complex, Class II, DO Alpha HLA-DQA2 Major Histocompatibility Complex, Class II, DQ Alpha 2

HLA-DOB Major Histocompatibility Complex, Class II, DO Beta HLA-DMA Major Histocompatibility Complex, Class II, DM Alpha HLA-DQA1 Major Histocompatibility Complex, Class II, DQ Alpha 1

HLA-DRA Major Histocompatibility Complex, Class II, DR Alpha hsa04940 **Type I Diabetes Mellitus**

HLA-DQB1 Major Histocompatibility Complex, Class II, DQ Beta 1

HLA-DRB3 Major Histocompatibility Complex, Class II, DR Beta 3

HLA-DRB5 Major Histocompatibility Complex, Class II, DR Beta 5

HLA-DPA1 Major Histocompatibility Complex, Class II, DP Alpha 1

HLA-DPB1 Major Histocompatibility Complex, Class II, DP Beta 1

HLA-DMB Major Histocompatibility Complex, Class II, DM Beta HLA-DOA Major Histocompatibility Complex, Class II, DO Alpha HLA-DQA2 Major Histocompatibility Complex, Class II, DQ Alpha 2

HLA-DOB Major Histocompatibility Complex, Class II, DO Beta HLA-DMA Major Histocompatibility Complex, Class II, DM Alpha HLA-DQA1 Major Histocompatibility Complex, Class II, DQ Alpha 1

HLA-DRA Major Histocompatibility Complex, Class II, DR Alpha

GO:0032395 **MHC Class II Receptor Activity**

HLA-DQB1 Major Histocompatibility Complex, Class II, DQ Beta 1

HLA-DRB3 Major Histocompatibility Complex, Class II, DR Beta 3

HLA-DPA1 Major Histocompatibility Complex, Class II, DP Alpha 1

HLA-DPB1 Major Histocompatibility Complex, Class II, DP Beta 1

HLA-DOA Major Histocompatibility Complex, Class II, DO Alpha

HLA-DQA2 Major Histocompatibility Complex, Class II, DQ Alpha 2

HLA-DOB Major Histocompatibility Complex, Class II, DO Beta HLA-DMA Major Histocompatibility Complex, Class II, DM Alpha HLA-DQA1 Major Histocompatibility Complex, Class II, DQ Alpha 1

HLA-DRA Major Histocompatibility Complex, Class II, DR Alpha

GO:0042611 **MHC Protein Complex**

HLA-DQB1 Major Histocompatibility Complex, Class II, DQ Beta 1

HLA-DRB3 Major Histocompatibility Complex, Class II, DR Beta 3

HLA-DMB Major Histocompatibility Complex, Class II, DM Beta HLA-DMA Major Histocompatibility Complex, Class II, DM Alpha HLA-DQA2 Major Histocompatibility Complex, Class II, DQ Alpha 2

HLA-DQA1 Major Histocompatibility Complex, Class II, DQ Alpha 1

AZGP1 Alpha-2-Glycoprotein 1, Zinc-Binding

HLA-DRB5 Major Histocompatibility Complex, Class II, DR Beta 5

HLA-DPA1 Major Histocompatibility Complex, Class II, DP Alpha 1

HLA-DPB1 Major Histocompatibility Complex, Class II, DP Beta 1

HLA-DOA Major Histocompatibility Complex, Class II, DO Alpha HLA-DOB Major Histocompatibility Complex, Class II, DO Beta HLA-DRA Major Histocompatibility Complex, Class II, DR Alpha

hsa04672 **Intestinal Immune Network For IgA Productionn**

HLA-DQB1 Major Histocompatibility Complex, Class II, DQ Beta 1

HLA-DRB3 Major Histocompatibility Complex, Class II, DR Beta 3

HLA-DRB5 Major Histocompatibility Complex, Class II, DR Beta 5

HLA-DPA1 Major Histocompatibility Complex, Class II, DP Alpha 1

HLA-DPB1 Major Histocompatibility Complex, Class II, DP Beta 1

HLA-DMB Major Histocompatibility Complex, Class II, DM Beta HLA-DOA Major Histocompatibility Complex, Class II, DO Alpha HLA-DQA2 Major Histocompatibility Complex, Class II, DQ Alpha 2

HLA-DOB Major Histocompatibility Complex, Class II, DO Beta HLA-DMA Major Histocompatibility Complex, Class II, DM Alpha HLA-DQA1 Major Histocompatibility Complex, Class II, DQ Alpha 1

HLA-DRA Major Histocompatibility Complex, Class II, DR Alpha

hsa05320 **Autoimmune Thyroid Disease**

|  | HLA-DQB1  HLA-DRB3  HLA-DRB5  HLA-DPA1  HLA-DPB1  HLA-DMB HLA-DOA HLA-DQA2  HLA-DOB HLA-DMA HLA-DQA1  HLA-DRA | Major Histocompatibility Complex, Class II, DQ Beta 1  Major Histocompatibility Complex, Class II, DR Beta 3  Major Histocompatibility Complex, Class II, DR Beta 5  Major Histocompatibility Complex, Class II, DP Alpha 1  Major Histocompatibility Complex, Class II, DP Beta 1  Major Histocompatibility Complex, Class II, DM Beta Major Histocompatibility Complex, Class II, DO Alpha Major Histocompatibility Complex, Class II, DQ Alpha 2  Major Histocompatibility Complex, Class II, DO Beta Major Histocompatibility Complex, Class II, DM Alpha Major Histocompatibility Complex, Class II, DQ Alpha 1  Major Histocompatibility Complex, Class II, DR Alpha |
| --- | --- | --- |
| hsa04514 | **Cell Adhesio** | **n Molecules (CAMs)** |
|  | HLA-DQB1 | Major Histocompatibility Complex, Class II, DQ Beta 1 |
|  | F11R | F11 Receptor |
|  | HLA-DRB3 | Major Histocompatibility Complex, Class II, DR Beta 3 |
|  | CLDN10 | Claudin 10 |
|  | HLA-DMB | Major Histocompatibility Complex, Class II, DM Beta |
|  | HLA-DMA | Major Histocompatibility Complex, Class II, DM Alpha |
|  | HLA-DQA2 | Major Histocompatibility Complex, Class II, DQ Alpha 2 |
|  | HLA-DQA1 | Major Histocompatibility Complex, Class II, DQ Alpha 1 |
|  | NCAM2 | Neural Cell Adhesion Molecule 2 |
|  | HLA-DRB5 | Major Histocompatibility Complex, Class II, DR Beta 5 |
|  | HLA-DPA1 | Major Histocompatibility Complex, Class II, DP Alpha 1 |
|  | HLA-DPB1 | Major Histocompatibility Complex, Class II, DP Beta 1 |
|  | HLA-DOA | Major Histocompatibility Complex, Class II, DO Alpha |
|  | HLA-DOB | Major Histocompatibility Complex, Class II, DO Beta |
|  | HLA-DRA | Major Histocompatibility Complex, Class II, DR Alpha |

GO:0019882 **Antigen Processing And Presentation**

HLA-DQB1 Major Histocompatibility Complex, Class II, DQ Beta 1

HLA-DRB3 Major Histocompatibility Complex, Class II, DR Beta 3

HLA-DMB Major Histocompatibility Complex, Class II, DM Beta HLA-DMA Major Histocompatibility Complex, Class II, DM Alpha HLA-DQA2 Major Histocompatibility Complex, Class II, DQ Alpha 2

HLA-DQA1 Major Histocompatibility Complex, Class II, DQ Alpha 1

CD74 CD74 Molecule, Major Histocompatibility Complex, Class II Invariant Chain

AZGP1 Alpha-2-Glycoprotein 1, Zinc-Binding

HLA-DRB5 Major Histocompatibility Complex, Class II, DR Beta 5

HLA-DPA1 Major Histocompatibility Complex, Class II, DP Alpha 1

HLA-DPB1 Major Histocompatibility Complex, Class II, DP Beta 1

HLA-DOA Major Histocompatibility Complex, Class II, DO Alpha HLA-DOB Major Histocompatibility Complex, Class II, DO Beta HLA-DRA Major Histocompatibility Complex, Class II, DR Alpha

hsa04612 **Antigen Processing And Presentation**

HLA-DQB1 Major Histocompatibility Complex, Class II, DQ Beta 1

HLA-DRB3 Major Histocompatibility Complex, Class II, DR Beta 3

HLA-DMB Major Histocompatibility Complex, Class II, DM Beta HLA-DMA Major Histocompatibility Complex, Class II, DM Alpha HLA-DQA2 Major Histocompatibility Complex, Class II, DQ Alpha 2

HLA-DQA1 Major Histocompatibility Complex, Class II, DQ Alpha 1

CD74 CD74 Molecule, Major Histocompatibility Complex, Class II Invariant Chain

HLA-DRB5 Major Histocompatibility Complex, Class II, DR Beta 5

HLA-DPA1 Major Histocompatibility Complex, Class II, DP Alpha 1

HLA-DPB1 Major Histocompatibility Complex, Class II, DP Beta 1

HLA-DOA Major Histocompatibility Complex, Class II, DO Alpha hsa05416 **Viral Myocarditis**

HLA-DQB1 Major Histocompatibility Complex, Class II, DQ Beta 1

HLA-DRB3 Major Histocompatibility Complex, Class II, DR Beta 3

HLA-DRB5 Major Histocompatibility Complex, Class II, DR Beta 5

HLA-DPA1 Major Histocompatibility Complex, Class II, DP Alpha 1

HLA-DPB1 Major Histocompatibility Complex, Class II, DP Beta 1

HLA-DMB Major Histocompatibility Complex, Class II, DM Beta HLA-DOA Major Histocompatibility Complex, Class II, DO Alpha HLA-DQA2 Major Histocompatibility Complex, Class II, DQ Alpha 2

HLA-DOB Major Histocompatibility Complex, Class II, DO Beta HLA-DMA Major Histocompatibility Complex, Class II, DM Alpha HLA-DQA1 Major Histocompatibility Complex, Class II, DQ Alpha 1

HLA-DRA Major Histocompatibility Complex, Class II, DR Alpha hsa05322 **Systemic Lupus Erythematosus**

HLA-DQB1 Major Histocompatibility Complex, Class II, DQ Beta 1

HLA-DRB3 Major Histocompatibility Complex, Class II, DR Beta 3

HLA-DRB5 Major Histocompatibility Complex, Class II, DR Beta 5

HLA-DPA1 Major Histocompatibility Complex, Class II, DP Alpha 1

HLA-DPB1 Major Histocompatibility Complex, Class II, DP Beta 1

HLA-DMB Major Histocompatibility Complex, Class II, DM Beta HLA-DOA Major Histocompatibility Complex, Class II, DO Alpha HLA-DQA2 Major Histocompatibility Complex, Class II, DQ Alpha 2

HLA-DOB Major Histocompatibility Complex, Class II, DO Beta HLA-DMA Major Histocompatibility Complex, Class II, DM Alpha HLA-DQA1 Major Histocompatibility Complex, Class II, DQ Alpha 1

HLA-DRA Major Histocompatibility Complex, Class II, DR Alpha

REACT_6900 **Signaling In Immune System**

HLA-DQB1 Major Histocompatibility Complex, Class II, DQ Beta 1

F11R F11 Receptor

HLA-DRB3 Major Histocompatibility Complex, Class II, DR Beta 3

HLA-DMB Major Histocompatibility Complex, Class II, DM Beta

PTEN Phosphatase And Tensin Homolog

HLA-DMA Major Histocompatibility Complex, Class II, DM Alpha

HLA-DQA2 Major Histocompatibility Complex, Class II, DQ Alpha 2

HLA-DQA1 Major Histocompatibility Complex, Class II, DQ Alpha 1

HLA-DRB5 Major Histocompatibility Complex, Class II, DR Beta 5

HLA-DPA1 Major Histocompatibility Complex, Class II, DP Alpha 1

HLA-DPB1 Major Histocompatibility Complex, Class II, DP Beta 1

HLA-DOA Major Histocompatibility Complex, Class II, DO Alpha

HLA-DOB Major Histocompatibility Complex, Class II, DO Beta

PTENP1 Phosphatase And Tensin Homolog Pseudogene 1 (Functional) HLA-DRA Major Histocompatibility Complex, Class II, DR Alpha

GO:0006955 **Immune Response**

HLA-DQB1 Major Histocompatibility Complex, Class II, DQ Beta 1

IFITM2 Interferon Induced Transmembrane Protein 2

IL7 Interleukin 7

HLA-DRB3 Major Histocompatibility Complex, Class II, DR Beta 3

HLA-DMB Major Histocompatibility Complex, Class II, DM Beta

HLA-DQA2 Major Histocompatibility Complex, Class II, DQ Alpha 2

HLA-DMA Major Histocompatibility Complex, Class II, DM Alpha

HLA-DQA1 Major Histocompatibility Complex, Class II, DQ Alpha 2

CD74 CD74 Molecule, Major Histocompatibility Complex, Class II Invariant Chain

IL31RA Interleukin 31 Receptor A

AZGP1 Alpha-2-Glycoprotein 1, Zinc-Binding

HLA-DRB5 Major Histocompatibility Complex, Class II, DR Beta 5

SEMA3C Semaphorin 3C

HLA-DPA1 Major Histocompatibility Complex, Class II, DP Alpha 1

HLA-DPB1 Major Histocompatibility Complex, Class II, DP Beta 1

HLA-DOA Major Histocompatibility Complex, Class II, DO Alpha HLA-DOB Major Histocompatibility Complex, Class II, DO Beta HLA-DRA Major Histocompatibility Complex, Class II, DR Alpha **Blood**

HLA-DQB1 Major Histocompatibility Complex, Class II, DQ Beta 1

CTSZ Cathepsin Z

TYRP1 Tyrosinase-Related Protein 1

GYPE Glycophorin E (MNS Blood Group)

HLA-DRB3 Major Histocompatibility Complex, Class II, DR Beta 3

PDE3A Phosphodiesterase 3A, CGMP-Inhibited

HLA-DMB Major Histocompatibility Complex, Class II, DM Beta

HLA-DQA2 Major Histocompatibility Complex, Class II, DQ Alpha 2

HLA-DQA1 Major Histocompatibility Complex, Class II, DQ Alpha 2

CYFIP2 Cytoplasmic FMR1 Interacting Protein 2

HLA-DRB5 Major Histocompatibility Complex, Class II, DR Beta 5

HLA-DPB1 Major Histocompatibility Complex, Class II, DP Beta 1

SLC14A1

Solute Carrier Family 14 (Urea Transporter), Member 1 (Kidd Blood Group)

HLA-DRA Major Histocompatibility Complex, Class II, DR Alpha

GO:0016339 **Calcium-Dependent Cell-Cell Adhesion**

PCDHB9 Protocadherin Beta 9

CDH13 Cadherin 13

PCDHB7 Protocadherin Beta 7

PCDHB5 Protocadherin Beta 5

PCDHB6 Protocadherin Beta 6

PCDHB3 Protocadherin Beta 3

PCDHB16 Protocadherin Beta 16

PCDHB2 Protocadherin Beta 2

PCDHB14 Protocadherin Beta 1

PCDHB11 Protocadherin Beta 11

GO:0007156 **Homophilic Cell Adhesion**

PCDHB9 Protocadherin Beta 9

PCDHB7 Protocadherin Beta 7

PCDHB8 Protocadherin Beta 8

PCDHB5 Protocadherin Beta 3

PCDHB6 Protocadherin Beta 6

PCDHB3 Protocadherin Beta 7

PCDHB15 Protocadherin Beta 15

PCDHB2 Protocadherin Beta 2

PCDHB14 Protocadherin Beta 14

PCDHB12 Protocadherin Beta 12

PCDHB11 Protocadherin Beta 11

CDH13 Cadherin 13

PCDHB18 Protocadherin Beta 18

PCDHB16 Protocadherin Beta 16

PCDHB17 Protocadherin Beta 17

GO:0016337 **Cell-Cell Adhesion**

PCDHB9 Protocadherin Beta 9

PCDHB7 Protocadherin Beta 7

PCDHB8 Protocadherin Beta 8

PCDHB5 Protocadherin Beta 5

PCDHB6 Protocadherin Beta 6

PCDHB3 Protocadherin Beta 3

PCDHB15 Protocadherin Beta 15

PCDHB2 Protocadherin Beta 2

PCDHB14 Protocadherin Beta 14

CLDN10 Claudin 10

PCDHB12 Protocadherin Beta 12

PCDHB11 Protocadherin Beta 11

GPR98 Adhesion G Protein-Coupled Receptor V1

CDH13 Cadherin 13

NCAM2 Neural Cell Adhesion Molecule 2

PCDHB18 Protocadherin Beta 18

PCDHB16 Protocadherin Beta 16

PCDHB17 Protocadherin Beta 17

CYFIP2 Cytoplasmic FMR1 Interacting Protein 2

GO:0007416 **Synaptogenesis**

PCDHB9 Protocadherin Beta 9

MYO6 Myosin VI

PCDHB5 Protocadherin Beta 5

PCDHB6 Protocadherin Beta 6

PCDHB3 Protocadherin Beta 3

PCDHB16 Protocadherin Beta 16

PCDHB2 Protocadherin Beta 2

PCDHB14 Protocadherin Beta 14

PCDHB11 Protocadherin Beta 11

GO:0007155 **Cell Adhesion**

PCDHB9 Protocadherin Beta 9

PTPRK Protein Tyrosine Phosphatase, Receptor Type, K F11R F11 Receptor

PCDHB7 Protocadherin Beta 7

PCDHB8 Protocadherin Beta 8

PCDHB5 Protocadherin Beta 5

PCDHB6 Protocadherin Beta 6

PCDHB3 Protocadherin Beta 3

PCDHB15 Protocadherin Beta 15

PCDHB2 Protocadherin Beta 2

PCDHB14 Protocadherin Beta 14

CLDN10 Claudin 10

PCDHB12 Protocadherin Beta 12

PCDHB11 Protocadherin Beta 11

GPR98 Adhesion G Protein-Coupled Receptor V1

AZGP1 Alpha-2-Glycoprotein 1, Zinc-Binding

NCAM2 Neural Cell Adhesion Molecule 2

CDH13 Cadherin 13

PCDHB18 Protocadherin Beta 18

PCDHB16 Protocadherin Beta 16

PCDHB17 Protocadherin Beta 17

CYFIP2 Cytoplasmic FMR1 Interacting Protein 2

SGCE Sarcoglycan, Epsilon

EMB Embigin

CYR61 Cysteine-Rich, Angiogenic Inducer, 61

GO:0022610 **Biological Adhesion**

PCDHB9 Protocadherin Beta 9

PTPRK Protein Tyrosine Phosphatase, Receptor Type, K F11R F11 Receptor

PCDHB7 Protocadherin Beta 7

PCDHB8 Protocadherin Beta 8

PCDHB5 Protocadherin Beta 5

PCDHB6 Protocadherin Beta 6

PCDHB3 Protocadherin Beta

PCDHB15 Protocadherin Beta 15

PCDHB2 Protocadherin Beta 2

PCDHB14 Protocadherin Beta 14

CLDN10 Claudin 10

PCDHB12 Protocadherin Beta 12

PCDHB11 Protocadherin Beta 11

GPR98 Adhesion G Protein-Coupled Receptor V1

AZGP1 Alpha-2-Glycoprotein 1, Zinc-Binding

NCAM2 Neural Cell Adhesion Molecule 2

CDH13 Cadherin 13

PCDHB18 Protocadherin Beta 18

PCDHB16 Protocadherin Beta 16

PCDHB17 Protocadherin Beta 17

CYFIP2 Cytoplasmic FMR1 Interacting Protein 2

SGCE Sarcoglycan, Epsilon

EMB Embigin

CYR61 Cysteine-Rich, Angiogenic Inducer, 61

GO:0050808 **Synapse Organization**

|  | PCDHB9  MYO6  PCDHB5  PCDHB6  PCDHB3  PCDHB16  PCDHB2  PCDHB14  PCDHB11 | Protocadherin Beta 9  Myosin VI Protocadherin Beta 5  Protocadherin Beta 6  Protocadherin Beta 3  Protocadherin Beta 16  Protocadherin Beta 2  Protocadherin Beta 14  Protocadherin Beta 11 |
| --- | --- | --- |
| GO:0043062 | **Extracellula** | **r Structure Organization** |
|  | PCDHB9 | Protocadherin Beta 9 |
|  | MYO6 | Myosin VI |
|  | PCDHB5 | Protocadherin Beta 5 |
|  | PCDHB6 | Protocadherin Beta 6 |
|  | PCDHB3 | Protocadherin Beta 3 |
|  | PCDHB16 | Protocadherin Beta 16 |
|  | PCDHB2 | Protocadherin Beta 2 |
|  | PCDHB14 | Protocadherin Beta 14 |
|  | PCDHB11 | Protocadherin Beta 11 |
|  | APBB2 | Amyloid Beta (A4) Precursor Protein-Binding, Family B, Member 2 |
|  | CYR61 | Cysteine-Rich, Angiogenic Inducer, 61 |

GO:0005509 **Calcium Ion Binding**

PCDHB9 Protocadherin Beta 9

PCDHB7 Protocadherin Beta 7

PCDHB8 Protocadherin Beta 8

PCDHB5 Protocadherin Beta 5

PCDHB6 Protocadherin Beta 6

PCDHB3 Protocadherin Beta 3

PCDHB15 Protocadherin Beta 15

PCDHB2 Protocadherin Beta 2

PCDHB14 Protocadherin Beta 14

PCDHB12 Protocadherin Beta 12

PCDHB11 Protocadherin Beta 11

GPR98 Adhesion G Protein-Coupled Receptor V1

CDH13 Cadherin 13

CLGN Calmegin

PCDHB18 Protocadherin Beta 18

PCDHB16 Protocadherin Beta 16

FSTL5 Follistatin-Like 5

PCDHB17 Protocadherin Beta 17

SGCE Sarcoglycan, Epsilon

GO:0007268 **Synaptic Transmission**

PCDHB9 Protocadherin Beta 9

MYO6 Myosin VI

|  | PCDHB5  PCDHB6  PCDHB3  PCDHB16  PCDHB2  APBA2  PCDHB14  PCDHB11 | Protocadherin Beta 5  Protocadherin Beta 6  Protocadherin Beta 3  Protocadherin Beta 16  Protocadherin Beta 2  Amyloid Beta (A4) Precursor Protein-Binding, Family A, Member 2  Protocadherin Beta 14  Protocadherin Beta 11 |
| --- | --- | --- |
| GO:0019226 | **Transmission** | **Of Nerve Impulse** |
|  | PCDHB9 | Protocadherin Beta 9 |
|  | MYO6 | Myosin VI |
|  | PCDHB5 | Protocadherin Beta 5 |
|  | PCDHB6 | Protocadherin Beta 6 |
|  | PCDHB3 | Protocadherin Beta 3 |
|  | PCDHB16 | Protocadherin Beta 16 |
|  | PCDHB2 | Protocadherin Beta 2 |
|  | APBA2 | Amyloid Beta (A4) Precursor Protein-Binding, Family A, Member 2 |
|  | PCDHB14 | Protocadherin Beta 14 |
|  | PCDHB11 | Protocadherin Beta 11 |

GO:0005886 **Plasma Membrane**

FHIT Fragile Histidine Triad

ADCY1 Adenylate Cyclase 1 (Brain)

GYPE Glycophorin E (MNS Blood Group)

AP1G2 Adaptor-Related Protein Complex 1, Gamma 2 Subunit

GPR160 G Protein-Coupled Receptor 160

HLA-DMB Major Histocompatibility Complex, Class II, DM Beta HLA-DMA Major Histocompatibility Complex, Class II, DM Alpha IL31RA Interleukin 31 Receptor A

AZGP1 Alpha-2-Glycoprotein 1, Zinc-Binding

PCDHB9 Protocadherin Beta 9

PTPRK Protein Tyrosine Phosphatase, Receptor Type, K F11R F11 Receptor

PCDHB7 Protocadherin Beta 7

PCDHB8 Protocadherin Beta 8

MYO6 Myosin VI

PCDHB5 Protocadherin Beta 5

MPDZ Multiple PDZ Domain Protein

PCDHB6 Protocadherin Beta 6

PCDHB3 Protocadherin Beta 3

MFI2

Antigen P97 (Melanoma Associated)

PCDHB2 Protocadherin Beta 2

HLA-DQA2 Major Histocompatibility Complex, Class II, DQ Alpha 2

GPR98 Adhesion G Protein-Coupled Receptor V1

HLA-DQA1 Major Histocompatibility Complex, Class II, DQ Alpha 2

NCAM2 Neural Cell Adhesion Molecule 2

HLA-DPA1 Major Histocompatibility Complex, Class II, DP Alpha 1

SGCE Sarcoglycan, Epsilon

TM4SF1 Transmembrane 4 L Six Family Member 1

HLA-DRA Major Histocompatibility Complex, Class II, DR Alpha

HLA-DQB1 Major Histocompatibility Complex, Class II, DQ Beta 1

COPZ2 Coatomer Protein Complex, Subunit Zeta 2

HLA-DRB3 Major Histocompatibility Complex, Class II, DR Beta 3

APH1B APH1B Gamma Secretase Subunit

PCDHB15 Protocadherin Beta 15

PCDHB14 Protocadherin Beta 14

CLDN10 Claudin 10

PCDHB12 Protocadherin Beta 12

PCDHB11 Protocadherin Beta 11

GPRC5A G Protein-Coupled Receptor, Class C, Group 5, Member A

CD74 CD74 Molecule, Major Histocompatibility Complex, Class II Invariant Chain

PCDHB18 Protocadherin Beta 18

PCDHB16 Protocadherin Beta 16

APBA2 Amyloid Beta (A4) Precursor Protein-Binding, Family A, Member 2

HLA-DRB5 Major Histocompatibility Complex, Class II, DR Beta 5

HLA-DPB1 Major Histocompatibility Complex, Class II, DP Beta 1

HLA-DOA Major Histocompatibility Complex, Class II, DO Alpha

PLA2R1 Phospholipase A2 Receptor 1

HLA-DOB Major Histocompatibility Complex, Class II, DO Beta

AXL AXL Receptor Tyrosine Kinase

EPHA5 EPH Receptor A5

CDH13 Cadherin 13

CYFIP2 Cytoplasmic FMR1 Interacting Protein 2

CXORF61 Cancer/Testis Antigen 83

SLC14A1

Solute Carrier Family 14 (Urea Transporter), Member 1 (Kidd Blood Group)

ATP6V0A4 ATPase, H+ Transporting, Lysosomal V0 Subunit A4

GO:0016021 **Integral To Membrane**

ADCY1 Adenylate Cyclase 1 (Brain)

GYPE Glycophorin E (MNS Blood Group) GPR160 G Protein-Coupled Receptor 160

HLA-DMB Major Histocompatibility Complex, Class II, DM Beta HLA-DMA Major Histocompatibility Complex, Class II, DM Alpha IL31RA Interleukin 31 Receptor A

TMEM108 Transmembrane Protein 108

ELOVL6 ELOVL Fatty Acid Elongase 6

NALCN Sodium Leak Channel, Non Selective UNC5C Unc-5 Homolog C (C. Elegans) ZDHHC2 Zinc Finger, DHHC-Type Containing 2

PCDHB9 Protocadherin Beta 9

F11R F11 Receptor

PTPRK Protein Tyrosine Phosphatase, Receptor Type, K KIAA1324L KIAA1324-Like

PCDHB7 Protocadherin Beta 7

PCDHB8 Protocadherin Beta 8

PCDHB5 Protocadherin Beta 5

PCDHB6 Protocadherin Beta 6

PCDHB3 Protocadherin Beta 3

MFI2 Antigen P97 (Melanoma Associated) PCDHB2 Protocadherin Beta 2

HLA-DQA2 Major Histocompatibility Complex, Class II, DQ Alpha 2

HLA-DQA1 Major Histocompatibility Complex, Class II, DQ Alpha 2

GPR98 Adhesion G Protein-Coupled Receptor V1

TPTE Transmembrane Phosphatase With Tensin Homology

NCAM2 Neural Cell Adhesion Molecule 2

PLEKHH2

Pleckstrin Homology Domain Containing, Family H (With MyTH4 Domain) Member 2

SGCE Sarcoglycan, Epsilon

HLA-DPA1 Major Histocompatibility Complex, Class II, DP Alpha 1

TM4SF1 Transmembrane 4 L Six Family Member 1

SLITRK6 SLIT And NTRK-Like Family, Member 6

HLA-DRA Major Histocompatibility Complex, Class II, DR Alpha

HLA-DQB1 Major Histocompatibility Complex, Class II, DQ Beta 1

DCC DCC Netrin 1 Receptor

CYP2U1 Cytochrome P450, Family 2, Subfamily U, Polypeptide 1

TYRP1 Tyrosinase-Related Protein 1

IFITM2 Interferon Induced Transmembrane Protein 2

HLA-DRB3 Major Histocompatibility Complex, Class II, DR Beta 3

PTPLAD2 protein tyrosine phosphatase-like protein PTPLAD2

APH1B APH1B Gamma Secretase Subunit

PCDHB15 Protocadherin Beta 15

PCDHB14 Protocadherin Beta 14

CLDN10 Claudin 10

PCDHB12 Protocadherin Beta 12

PCDHB11 Protocadherin Beta 11

GPRC5A G Protein-Coupled Receptor, Class C, Group 5, Member A

CD74 CD74 Molecule, Major Histocompatibility Complex, Class II Invariant Chain

LINGO2 Leucine Rich Repeat And Ig Domain Containing 2

TSC22D3 TSC22 Domain Family, Member 3

PCDHB18 Protocadherin Beta 18

PCDHB16 Protocadherin Beta 16

PCDHB17 Protocadherin Beta 17

SLC4A8 Solute Carrier Family 4, Sodium Bicarbonate Cotransporter, Member 8

HLA-DRB5 Major Histocompatibility Complex, Class II, DR Beta 5

ARMCX1 Armadillo Repeat Containing, X-Linked 1

EMB Embigin

HLA-DPB1 Major Histocompatibility Complex, Class II, DP Beta 1

PLA2R1 Phospholipase A2 Receptor 1

HLA-DOA Major Histocompatibility Complex, Class II, DO Alpha HLA-DOB Major Histocompatibility Complex, Class II, DO Beta AXL AXL Receptor Tyrosine Kinase

EPHA5 EPH Receptor A5

CLGN Calmegin

CXORF61 Cancer/Testis Antigen 83

SLC14A1

Solute Carrier Family 14 (Urea Transporter), Member 1 (Kidd Blood Group)

SLC46A3 Solute Carrier Family 46, Member 3

ATP6V0A4 ATPase, H+ Transporting, Lysosomal V0 Subunit A4

GO:0044459 **Plasma Membrane Part**

HLA-DQB1 Major Histocompatibility Complex, Class II, DQ Beta 1

COPZ2 Coatomer Protein Complex, Subunit Zeta 2

GYPE Glycophorin E (MNS Blood Group)

HLA-DRB3 Major Histocompatibility Complex, Class II, DR Beta 3

PCDHB15 Protocadherin Beta 15

CLDN10 Claudin 10

PCDHB12 Protocadherin Beta 12

HLA-DMB Major Histocompatibility Complex, Class II, DM Beta

PCDHB11 Protocadherin Beta 11

GPRC5A G Protein-Coupled Receptor, Class C, Group 5, Member A HLA-DMA Major Histocompatibility Complex, Class II, DM Alpha

CD74 CD74 Molecule, Major Histocompatibility Complex, Class II Invariant Chain

AZGP1 Alpha-2-Glycoprotein 1, Zinc-Binding

HLA-DRB5 Major Histocompatibility Complex, Class II, DR Beta 5

HLA-DPB1 Major Histocompatibility Complex, Class II, DP Beta 1

PLA2R1 Phospholipase A2 Receptor 1

HLA-DOA Major Histocompatibility Complex, Class II, DO Alpha HLA-DOB Major Histocompatibility Complex, Class II, DO Beta PTPRK Protein Tyrosine Phosphatase, Receptor Type, K F11R F11 Receptor

MYO6 Myosin VI

MPDZ Multiple PDZ Domain Protein

PCDHB6 Protocadherin Beta 6

PCDHB3 Protocadherin Beta 3

MFI2 Antigen P97 (Melanoma Associated) AXL AXL Receptor Tyrosine Kinase PCDHB2 Protocadherin Beta 2

HLA-DQA2 Major Histocompatibility Complex, Class II, DQ Alpha 2

HLA-DQA1 Major Histocompatibility Complex, Class II, DQ Alpha 1

EPHA5 EPH Receptor A5

CDH13 Cadherin 13

CYFIP2 Cytoplasmic FMR1 Interacting Protein 2

HLA-DPA1 Major Histocompatibility Complex, Class II, DP Alpha 1

SGCE Sarcoglycan, Epsilon

TM4SF1 Transmembrane 4 L Six Family Member 1

SLC14A1

Solute Carrier Family 14 (Urea Transporter), Member 1 (Kidd Blood Group)

ATP6V0A4 ATPase, H+ Transporting, Lysosomal V0 Subunit A4

HLA-DRA Major Histocompatibility Complex, Class II, DR Alpha

GO:0005887 **Integral To Plasma Membrane**

PTPRK Protein Tyrosine Phosphatase, Receptor Type, K GYPE Glycophorin E (MNS Blood Group)

PCDHB6 Protocadherin Beta 6

HLA-DRB3 Major Histocompatibility Complex, Class II, DR Beta 3

PCDHB3 Protocadherin Beta 3

MFI2 Antigen P97 (Melanoma Associated) AXL AXL Receptor Tyrosine Kinase PCDHB15 Protocadherin Beta 15

PCDHB2 Protocadherin Beta 2

PCDHB12 Protocadherin Beta 12

PCDHB11 Protocadherin Beta 11

GPRC5A G Protein-Coupled Receptor, Class C, Group 5, Member A HLA-DQA2 Major Histocompatibility Complex, Class II, DQ Alpha 2

HLA-DQA1 Major Histocompatibility Complex, Class II, DQ Alpha 1

EPHA5 EPH Receptor A5

HLA-DPA1 Major Histocompatibility Complex, Class II, DP Alpha 1

SGCE Sarcoglycan, Epsilon

TM4SF1 Transmembrane 4 L Six Family Member 1

SLC14A1

Solute Carrier Family 14 (Urea Transporter), Member 1 (Kidd Blood Group)

PLA2R1 Phospholipase A2 Receptor 1

HLA-DRA Major Histocompatibility Complex, Class II, DR Alpha

GO:0031226 **Intrinsic To Plasma Membrane**

PTPRK Protein Tyrosine Phosphatase, Receptor Type, K GYPE Glycophorin E (MNS Blood Group)

PCDHB6 Protocadherin Beta 6

HLA-DRB3 Major Histocompatibility Complex, Class II, DR Beta 3

PCDHB3 Protocadherin Beta 3

MFI2 Antigen P97 (Melanoma Associated) AXL AXL Receptor Tyrosine Kinase PCDHB15 Protocadherin Beta 15

PCDHB2 Protocadherin Beta 2

PCDHB12 Protocadherin Beta 12

PCDHB11 Protocadherin Beta 11

GPRC5A G Protein-Coupled Receptor, Class C, Group 5, Member A HLA-DQA2 Major Histocompatibility Complex, Class II, DQ Alpha 2

HLA-DQA1 Major Histocompatibility Complex, Class II, DQ Alpha 1

EPHA5 EPH Receptor A5

HLA-DPA1 Major Histocompatibility Complex, Class II, DP Alpha 1

SGCE Sarcoglycan, Epsilon

TM4SF1 Transmembrane 4 L Six Family Member 1

SLC14A1

Solute Carrier Family 14 (Urea Transporter), Member 1 (Kidd Blood Group)

PLA2R1 Phospholipase A2 Receptor 1

HLA-DRA Major Histocompatibility Complex, Class II, DR Alpha

GO:0042825 **TAP Complex**

HLA-DMB Major Histocompatibility Complex, Class II, DM Beta HLA-DOB Major Histocompatibility Complex, Class II, DO Beta HLA-DMA Major Histocompatibility Complex, Class II, DM Alpha HLA-DRA Major Histocompatibility Complex, Class II, DR Alpha

GO:0042824 **MHC class I Peptide Loading Complex**

HLA-DMB Major Histocompatibility Complex, Class II, DM Beta HLA-DOB Major Histocompatibility Complex, Class II, DO Beta HLA-DMA Major Histocompatibility Complex, Class II, DM Alpha HLA-DRA Major Histocompatibility Complex, Class II, DR Alpha

GO:0042287 **MHC Protein Binding**

HLA-DMB Major Histocompatibility Complex, Class II, DM Beta HLA-DOB Major Histocompatibility Complex, Class II, DO Beta HLA-DMA Major Histocompatibility Complex, Class II, DM Alpha

CD74

CD74 Molecule, Major Histocompatibility Complex, Class II Invariant Chain

HLA-DRA Major Histocompatibility Complex, Class II, DR Alpha

GO:0042288 **MHC Class I Protein Binding**

|  | HLA-DMB | Major Histocompatibility Complex, Class II, DM Beta |
| --- | --- | --- |
|  | HLA-DOB | Major Histocompatibility Complex, Class II, DO Beta |
|  | HLA-DMA | Major Histocompatibility Complex, Class II, DM Alpha |
|  | HLA-DRA | Major Histocompatibility Complex, Class II, DR Alpha |
| GO:0005773 | **Vacuole** |  |
|  | CTSZ | Cathepsin Z |

GAA Glucosidase, Alpha; Acid

HLA-DMB Major Histocompatibility Complex, Class II, DM Beta

ATP6V0A4 ATPase, H+ Transporting, Lysosomal V0 Subunit A4

HLA-DOB Major Histocompatibility Complex, Class II, DO Beta

HLA-DMA CD74

Major Histocompatibility Complex, Class II, DM Alpha

CD74 Molecule, Major Histocompatibility Complex, Class II Invariant Chain

HLA-DRA Major Histocompatibility Complex, Class II, DR Alpha

GO:0000323 **Lytic Vacuole**

CTSZ Cathepsin Z

GAA Glucosidase, Alpha; Acid

HLA-DMB Major Histocompatibility Complex, Class II, DM Beta HLA-DOB Major Histocompatibility Complex, Class II, DO Beta HLA-DMA Major Histocompatibility Complex, Class II, DM Alpha

CD74

CD74 Molecule, Major Histocompatibility Complex, Class II Invariant Chain

HLA-DRA Major Histocompatibility Complex, Class II, DR Alpha

GO:0005764 **Lysosome**

CTSZ Cathepsin Z

GAA Glucosidase, Alpha; Acid

HLA-DMB Major Histocompatibility Complex, Class II, DM Beta HLA-DOB Major Histocompatibility Complex, Class II, DO Beta HLA-DMA Major Histocompatibility Complex, Class II, DM Alpha

CD74

CD74 Molecule, Major Histocompatibility Complex, Class II Invariant Chain

HLA-DRA Major Histocompatibility Complex, Class II, DR Alpha

**Upregulated G10 vs A7**

GO:0042613 **MHC class II Protein Complex**

|  | HLA-DQB1  HLA-DRB3  HLA-DRB5  HLA-DPA1  HLA-DPB1  HLA-DMB HLA-DOA HLA-DQA2  HLA-DOB HLA-DMA HLA-DQA1  HLA-DRA | Major Histocompatibility Complex, Class II, DQ Beta 1  Major Histocompatibility Complex, Class II, DR Beta 3  Major Histocompatibility Complex, Class II, DR Beta 5  Major Histocompatibility Complex, Class II, DP Alpha 1  Major Histocompatibility Complex, Class II, DP Beta 1  Major Histocompatibility Complex, Class II, DM Beta Major Histocompatibility Complex, Class II, DO Alpha Major Histocompatibility Complex, Class II, DQ Alpha 2  Major Histocompatibility Complex, Class II, DO Beta Major Histocompatibility Complex, Class II, DM Alpha Major Histocompatibility Complex, Class II, DQ Alpha 1  Major Histocompatibility Complex, Class II, DR Alpha |
| --- | --- | --- |
| hsa05310 | **Asthma** |  |
|  | HLA-DQB1  HLA-DRB3  HLA-DRB5  HLA-DPA1  HLA-DPB1  HLA-DMB HLA-DOA HLA-DQA2  HLA-DOB HLA-DMA HLA-DQA1  HLA-DRA | Major Histocompatibility Complex, Class II, DQ Beta 1  Major Histocompatibility Complex, Class II, DR Beta 3  Major Histocompatibility Complex, Class II, DR Beta 5  Major Histocompatibility Complex, Class II, DP Alpha 1  Major Histocompatibility Complex, Class II, DP Beta 1  Major Histocompatibility Complex, Class II, DM Beta Major Histocompatibility Complex, Class II, DO Alpha Major Histocompatibility Complex, Class II, DQ Alpha 2  Major Histocompatibility Complex, Class II, DO Beta Major Histocompatibility Complex, Class II, DM Alpha Major Histocompatibility Complex, Class II, DQ Alpha 1  Major Histocompatibility Complex, Class II, DR Alpha |

GO:0002504

**Antigen Processing And Presentation Of Peptide Or Polysaccharide Antigen Via MHC Class II**

HLA-DQB1 Major Histocompatibility Complex, Class II, DQ Beta 1

HLA-DRB3 Major Histocompatibility Complex, Class II, DR Beta 3

HLA-DMB Major Histocompatibility Complex, Class II, DM Beta HLA-DMA Major Histocompatibility Complex, Class II, DM Alpha HLA-DQA2 Major Histocompatibility Complex, Class II, DQ Alpha 2

HLA-DQA1 Major Histocompatibility Complex, Class II, DQ Alpha 1

CD74

CD74 Molecule, Major Histocompatibility Complex, Class II Invariant Chain

HLA-DRB5 Major Histocompatibility Complex, Class II, DR Beta 5

HLA-DPA1 Major Histocompatibility Complex, Class II, DP Alpha 1

HLA-DPB1 Major Histocompatibility Complex, Class II, DP Beta 1

HLA-DOA Major Histocompatibility Complex, Class II, DO Alpha HLA-DOB Major Histocompatibility Complex, Class II, DO Beta HLA-DRA Major Histocompatibility Complex, Class II, DR Alpha

hsa05330 **Allograft Rejection**

HLA-DQB1 Major Histocompatibility Complex, Class II, DQ Beta 1

HLA-DRB3 Major Histocompatibility Complex, Class II, DR Beta 3

HLA-DRB5 Major Histocompatibility Complex, Class II, DR Beta 5

HLA-DPA1 Major Histocompatibility Complex, Class II, DP Alpha 1

HLA-DPB1 Major Histocompatibility Complex, Class II, DP Beta 1

HLA-DMB Major Histocompatibility Complex, Class II, DM Beta HLA-DOA Major Histocompatibility Complex, Class II, DO Alpha HLA-DQA2 Major Histocompatibility Complex, Class II, DQ Alpha 2

HLA-DOB Major Histocompatibility Complex, Class II, DO Beta HLA-DMA Major Histocompatibility Complex, Class II, DM Alpha HLA-DQA1 Major Histocompatibility Complex, Class II, DQ Alpha 1

HLA-DRA Major Histocompatibility Complex, Class II, DR Alpha hsa05332 **Graft-Versus-Host Disease**

HLA-DQB1 Major Histocompatibility Complex, Class II, DQ Beta 1

HLA-DRB3 Major Histocompatibility Complex, Class II, DR Beta 3

HLA-DRB5 Major Histocompatibility Complex, Class II, DR Beta 5

HLA-DPA1 Major Histocompatibility Complex, Class II, DP Alpha 1

HLA-DPB1 Major Histocompatibility Complex, Class II, DP Beta 1

HLA-DMB Major Histocompatibility Complex, Class II, DM Beta HLA-DOA Major Histocompatibility Complex, Class II, DO Alpha HLA-DQA2 Major Histocompatibility Complex, Class II, DQ Alpha 2

HLA-DOB Major Histocompatibility Complex, Class II, DO Beta HLA-DMA Major Histocompatibility Complex, Class II, DM Alpha HLA-DQA1 Major Histocompatibility Complex, Class II, DQ Alpha 1

HLA-DRA Major Histocompatibility Complex, Class II, DR Alpha hsa04940 **Type I Diabetes Mellitus**

HLA-DQB1 Major Histocompatibility Complex, Class II, DQ Beta 1

HLA-DRB3 Major Histocompatibility Complex, Class II, DR Beta 3

HLA-DRB5 Major Histocompatibility Complex, Class II, DR Beta 5

HLA-DPA1 Major Histocompatibility Complex, Class II, DP Alpha 1

HLA-DPB1 Major Histocompatibility Complex, Class II, DP Beta 1

HLA-DMB Major Histocompatibility Complex, Class II, DM Beta HLA-DOA Major Histocompatibility Complex, Class II, DO Alpha HLA-DQA2 Major Histocompatibility Complex, Class II, DQ Alpha 2

HLA-DOB Major Histocompatibility Complex, Class II, DO Beta

HLA-DMA Major Histocompatibility Complex, Class II, DM Alpha

HLA-DQA1 Major Histocompatibility Complex, Class II, DQ Alpha 1

HLA-DRA Major Histocompatibility Complex, Class II, DR Alpha

GO:0032395 **MHC Class II Receptor Activity**

HLA-DQB1 Major Histocompatibility Complex, Class II, DQ Beta 1

HLA-DRB3 Major Histocompatibility Complex, Class II, DR Beta 3

HLA-DPA1 Major Histocompatibility Complex, Class II, DP Alpha 1

HLA-DPB1 Major Histocompatibility Complex, Class II, DP Beta 1

HLA-DOA Major Histocompatibility Complex, Class II, DO Alpha

HLA-DQA2 Major Histocompatibility Complex, Class II, DQ Alpha 2

HLA-DOB Major Histocompatibility Complex, Class II, DO Beta HLA-DMA Major Histocompatibility Complex, Class II, DM Alpha HLA-DQA1 Major Histocompatibility Complex, Class II, DQ Alpha 1

HLA-DRA Major Histocompatibility Complex, Class II, DR Alpha hsa04672 **Intestinal Immune Network For IgA Production**

HLA-DQB1 Major Histocompatibility Complex, Class II, DQ Beta 1

HLA-DRB3 Major Histocompatibility Complex, Class II, DR Beta 3

HLA-DRB5 Major Histocompatibility Complex, Class II, DR Beta 5

HLA-DPA1 Major Histocompatibility Complex, Class II, DP Alpha 1

HLA-DPB1 Major Histocompatibility Complex, Class II, DP Beta 1

HLA-DMB Major Histocompatibility Complex, Class II, DM Beta HLA-DOA Major Histocompatibility Complex, Class II, DO Alpha HLA-DQA2 Major Histocompatibility Complex, Class II, DQ Alpha 2

HLA-DOB Major Histocompatibility Complex, Class II, DO Beta HLA-DMA Major Histocompatibility Complex, Class II, DM Alpha HLA-DQA1 Major Histocompatibility Complex, Class II, DQ Alpha 1

HLA-DRA Major Histocompatibility Complex, Class II, DR Alpha

GO:0042611 **MHC Protein Complex**

HLA-DQB1 Major Histocompatibility Complex, Class II, DQ Beta 1

HLA-DRB3 Major Histocompatibility Complex, Class II, DR Beta 3

HLA-DRB5 Major Histocompatibility Complex, Class II, DR Beta 5

HLA-DPA1 Major Histocompatibility Complex, Class II, DP Alpha 1

HLA-DPB1 Major Histocompatibility Complex, Class II, DP Beta 1

HLA-DMB Major Histocompatibility Complex, Class II, DM Beta HLA-DOA Major Histocompatibility Complex, Class II, DO Alpha HLA-DQA2 Major Histocompatibility Complex, Class II, DQ Alpha 2

HLA-DOB Major Histocompatibility Complex, Class II, DO Beta HLA-DMA Major Histocompatibility Complex, Class II, DM Alpha HLA-DQA1 Major Histocompatibility Complex, Class II, DQ Alpha 1

HLA-DRA Major Histocompatibility Complex, Class II, DR Alpha hsa05320 **Autoimmune Thyroid Disease**

HLA-DQB1 Major Histocompatibility Complex, Class II, DQ Beta 1

HLA-DRB3 Major Histocompatibility Complex, Class II, DR Beta 3

HLA-DRB5 Major Histocompatibility Complex, Class II, DR Beta 5

HLA-DPA1 Major Histocompatibility Complex, Class II, DP Alpha 1

HLA-DPB1 Major Histocompatibility Complex, Class II, DP Beta 1

HLA-DMB Major Histocompatibility Complex, Class II, DM Beta HLA-DOA Major Histocompatibility Complex, Class II, DO Alpha HLA-DQA2 Major Histocompatibility Complex, Class II, DQ Alpha 2

HLA-DOB Major Histocompatibility Complex, Class II, DO Beta

HLA-DMA Major Histocompatibility Complex, Class II, DM Alpha

HLA-DQA1 Major Histocompatibility Complex, Class II, DQ Alpha 1

HLA-DRA Major Histocompatibility Complex, Class II, DR Alpha hsa04612 **Antigen Processing And Presentation**

HLA-DQB1 Major Histocompatibility Complex, Class II, DQ Beta 1

HLA-DRB3 Major Histocompatibility Complex, Class II, DR Beta 3

HLA-DMB Major Histocompatibility Complex, Class II, DM Beta HLA-DMA Major Histocompatibility Complex, Class II, DM Alpha HLA-DQA2 Major Histocompatibility Complex, Class II, DQ Alpha 2

HLA-DQA1 Major Histocompatibility Complex, Class II, DQ Alpha 1

CD74

CD74 Molecule, Major Histocompatibility Complex, Class II Invariant Chain

HLA-DRB5 Major Histocompatibility Complex, Class II, DR Beta 5

HLA-DPA1 Major Histocompatibility Complex, Class II, DP Alpha 1

HLA-DPB1 Major Histocompatibility Complex, Class II, DP Beta 1

HLA-DOA Major Histocompatibility Complex, Class II, DO Alpha HLA-DOB Major Histocompatibility Complex, Class II, DO Beta HLA-DRA Major Histocompatibility Complex, Class II, DR Alpha

hsa05416 **Viral Myocarditis**

|  | HLA-DQB1  HLA-DRB3  HLA-DRB5  HLA-DPA1  HLA-DPB1  HLA-DMB HLA-DOA HLA-DQA2  HLA-DOB HLA-DMA HLA-DQA1  HLA-DRA | Major Histocompatibility Complex, Class II, DQ Beta 1  Major Histocompatibility Complex, Class II, DR Beta 3  Major Histocompatibility Complex, Class II, DR Beta 5  Major Histocompatibility Complex, Class II, DP Alpha 1  Major Histocompatibility Complex, Class II, DP Beta 1  Major Histocompatibility Complex, Class II, DM Beta Major Histocompatibility Complex, Class II, DO Alpha Major Histocompatibility Complex, Class II, DQ Alpha 2  Major Histocompatibility Complex, Class II, DO Beta Major Histocompatibility Complex, Class II, DM Alpha Major Histocompatibility Complex, Class II, DQ Alpha 1  Major Histocompatibility Complex, Class II, DR Alpha |
| --- | --- | --- |
| hsa04514 | **Cell Adhesio** | **n Molecules (CAMs)** |
|  | HLA-DQB1 | Major Histocompatibility Complex, Class II, DQ Beta 1 |
|  | HLA-DRB3 | Major Histocompatibility Complex, Class II, DR Beta 3 |
|  | HLA-DMB | Major Histocompatibility Complex, Class II, DM Beta |
|  | HLA-DMA | Major Histocompatibility Complex, Class II, DM Alpha |
|  | HLA-DQA2 | Major Histocompatibility Complex, Class II, DQ Alpha 2 |
|  | HLA-DQA1 | Major Histocompatibility Complex, Class II, DQ Alpha 1 |
|  | NRCAM | Neuronal Cell Adhesion Molecule |
|  | NCAM2 | Neural Cell Adhesion Molecule 2 |
|  | HLA-DRB5 | Major Histocompatibility Complex, Class II, DR Beta 5 |
|  | HLA-DPA1 | Major Histocompatibility Complex, Class II, DP Alpha 1 |
|  | HLA-DPB1 | Major Histocompatibility Complex, Class II, DP Beta 1 |
|  | HLA-DOA | Major Histocompatibility Complex, Class II, DO Alpha |
|  | HLA-DOB | Major Histocompatibility Complex, Class II, DO Beta |
|  | HLA-DRA | Major Histocompatibility Complex, Class II, DR Alpha |

GO:0019882 **Antigen Processing And Presentation**

HLA-DQB1 Major Histocompatibility Complex, Class II, DQ Beta 1

HLA-DRB3 Major Histocompatibility Complex, Class II, DR Beta 3

HLA-DMB Major Histocompatibility Complex, Class II, DM Beta

HLA-DMA Major Histocompatibility Complex, Class II, DM Alpha

HLA-DQA2 Major Histocompatibility Complex, Class II, DQ Alpha 2

HLA-DQA1 Major Histocompatibility Complex, Class II, DQ Alpha 1

CD74

CD74 Molecule, Major Histocompatibility Complex, Class II Invariant Chain

HLA-DRB5 Major Histocompatibility Complex, Class II, DR Beta 5

HLA-DPA1 Major Histocompatibility Complex, Class II, DP Alpha 1

HLA-DPB1 Major Histocompatibility Complex, Class II, DP Beta 1

HLA-DOA Major Histocompatibility Complex, Class II, DO Alpha HLA-DOB Major Histocompatibility Complex, Class II, DO Beta HLA-DRA Major Histocompatibility Complex, Class II, DR Alpha

hsa05322 **Systemic Lupus Erythematosus**

HLA-DQB1 Major Histocompatibility Complex, Class II, DQ Beta 1

HLA-DRB3 Major Histocompatibility Complex, Class II, DR Beta 3

HLA-DRB5 Major Histocompatibility Complex, Class II, DR Beta 5

HLA-DPA1 Major Histocompatibility Complex, Class II, DP Alpha 1

HLA-DPB1 Major Histocompatibility Complex, Class II, DP Beta 1

HLA-DMB Major Histocompatibility Complex, Class II, DM Beta HLA-DOA Major Histocompatibility Complex, Class II, DO Alpha HLA-DQA2 Major Histocompatibility Complex, Class II, DQ Alpha 2

HLA-DOB Major Histocompatibility Complex, Class II, DO Beta HLA-DMA Major Histocompatibility Complex, Class II, DM Alpha HLA-DQA1 Major Histocompatibility Complex, Class II, DQ Alpha 1

HLA-DRA Major Histocompatibility Complex, Class II, DR Alpha

REACT_6900 **Signaling In Immune System**

HLA-DQB1 Major Histocompatibility Complex, Class II, DQ Beta 1

HLA-DRB3 Major Histocompatibility Complex, Class II, DR Beta 3

HLA-DMB Major Histocompatibility Complex, Class II, DM Beta

PTEN Phosphatase And Tensin Homolog

HLA-DMA Major Histocompatibility Complex, Class II, DM Alpha

HLA-DQA2 Major Histocompatibility Complex, Class II, DQ Alpha 2

HLA-DQA1 Major Histocompatibility Complex, Class II, DQ Alpha 1

HLA-DRB5 Major Histocompatibility Complex, Class II, DR Beta 5

RIPK2 Receptor-Interacting Serine-Threonine Kinase 2

HLA-DPA1 Major Histocompatibility Complex, Class II, DP Alpha 1

HLA-DPB1 Major Histocompatibility Complex, Class II, DP Beta 1

HLA-DOA Major Histocompatibility Complex, Class II, DO Alpha

HLA-DOB Major Histocompatibility Complex, Class II, DO Beta

PTENP1 Phosphatase And Tensin Homolog Pseudogene 1 (Functional) HLA-DRA Major Histocompatibility Complex, Class II, DR Alpha

GO:0006955 **Immune Response**

HLA-DQB1 Major Histocompatibility Complex, Class II, DQ Beta 1

HLA-DRB3 Major Histocompatibility Complex, Class II, DR Beta 3

HLA-DMB Major Histocompatibility Complex, Class II, DM Beta HLA-DMA Major Histocompatibility Complex, Class II, DM Alpha HLA-DQA2 Major Histocompatibility Complex, Class II, DQ Alpha 2

HLA-DQA1 Major Histocompatibility Complex, Class II, DQ Alpha 1

CD74

CD74 Molecule, Major Histocompatibility Complex, Class II Invariant Chain

IL31RA Interleukin 31 Receptor A

HLA-DRB5 Major Histocompatibility Complex, Class II, DR Beta 5

SEMA3C

Semaphorin 3C

HLA-DPA1 Major Histocompatibility Complex, Class II, DP Alpha 1

HLA-DPB1 Major Histocompatibility Complex, Class II, DP Beta 1

ODZ1 Teneurin Transmembrane Protein 1

HLA-DOA Major Histocompatibility Complex, Class II, DO Alpha HLA-DOB Major Histocompatibility Complex, Class II, DO Beta HLA-DRA Major Histocompatibility Complex, Class II, DR Alpha **Blood**

HLA-DQB1 Major Histocompatibility Complex, Class II, DQ Beta 1

CTSZ Cathepsin Z

GYPE Glycophorin E (MNS Blood Group)

HLA-DRB3 Major Histocompatibility Complex, Class II, DR Beta 3

HLA-DRB5 Major Histocompatibility Complex, Class II, DR Beta 5

HLA-DPB1 Major Histocompatibility Complex, Class II, DP Beta 1

HLA-DMB Major Histocompatibility Complex, Class II, DM Beta

SLC14A1

Solute Carrier Family 14 (Urea Transporter), Member 1 (Kidd Blood Group)

HLA-DQA2 Major Histocompatibility Complex, Class II, DQ Alpha 2

EMP1 Epithelial Membrane Protein 1

HLA-DQA1 Major Histocompatibility Complex, Class II, DQ Alpha 1

HLA-DRA Major Histocompatibility Complex, Class II, DR Alpha

GO:0044459 **Plasma Membrane Part**

HLA-DQB1 Major Histocompatibility Complex, Class II, DQ Beta 1

SLC5A3 Solute Carrier Family 5 (Sodium/Myo-Inositol Cotransporter), Member 3

COPZ2 Coatomer Protein Complex, Subunit Zeta 2

GYPE Glycophorin E (MNS Blood Group)

HLA-DRB3 Major Histocompatibility Complex, Class II, DR Beta 3

LPAR4 Lysophosphatidic Acid Receptor 4

HLA-DMB Major Histocompatibility Complex, Class II, DM Beta GPRC5A G Protein-Coupled Receptor, Class C, Group 5, Member A HLA-DMA Major Histocompatibility Complex, Class II, DM Alpha

CD74 CD74 Molecule, Major Histocompatibility Complex, Class II Invariant Chain

STARD13 StAR-Related Lipid Transfer (START) Domain Containing 13

NRCAM Neuronal Cell Adhesion Molecule

FAT1 FAT Atypical Cadherin 1

HLA-DRB5 Major Histocompatibility Complex, Class II, DR Beta 5

HLA-DPB1 Major Histocompatibility Complex, Class II, DP Beta 1

PLA2R1 Phospholipase A2 Receptor 1

ODZ1 Teneurin Transmembrane Protein 1

HLA-DOA Major Histocompatibility Complex, Class II, DO Alpha HLA-DOB Major Histocompatibility Complex, Class II, DO Beta FLRT3 Fibronectin Leucine Rich Transmembrane Protein 3

MYO6 Myosin VI

MPDZ Multiple PDZ Domain Protein

PCDHB3 Protocadherin Beta 3

MFI2 Antigen P97 (Melanoma Associated) AXL AXL Receptor Tyrosine Kinase

HLA-DQA2 Major Histocompatibility Complex, Class II, DQ Alpha 2

HLA-DQA1 Major Histocompatibility Complex, Class II, DQ Alpha 1

P2RX5 Purinergic Receptor P2X, Ligand Gated Ion Channel, 5

CDH13 Cadherin 13

CHRM3 Cholinergic Receptor, Muscarinic 3

HLA-DPA1 Major Histocompatibility Complex, Class II, DP Alpha 1

SGCE Sarcoglycan, Epsilon

TM4SF1 Transmembrane 4 L Six Family Member 1

SLC14A1

Solute Carrier Family 14 (Urea Transporter), Member 1 (Kidd Blood Group)

DST Dystonin

HLA-DRA Major Histocompatibility Complex, Class II, DR Alpha

GO:0005886 **Plasma Membrane**

HLA-DQB1 Major Histocompatibility Complex, Class II, DQ Beta 1

SLC5A3 Solute Carrier Family 5 (Sodium/Myo-Inositol Cotransporter), Member 3

COPZ2 Coatomer Protein Complex, Subunit Zeta 2

GYPE Glycophorin E (MNS Blood Group)

AP1G2 Adaptor-Related Protein Complex 1, Gamma 2 Subunit

HLA-DRB3 Major Histocompatibility Complex, Class II, DR Beta 3

APH1B APH1B Gamma Secretase Subunit

LPAR4 Lysophosphatidic Acid Receptor 4

HLA-DMB Major Histocompatibility Complex, Class II, DM Beta GPRC5A G Protein-Coupled Receptor, Class C, Group 5, Member A HLA-DMA Major Histocompatibility Complex, Class II, DM Alpha

CD74 CD74 Molecule, Major Histocompatibility Complex, Class II Invariant Chain

STARD13 StAR-Related Lipid Transfer (START) Domain Containing 13

IL31RA Interleukin 31 Receptor A

NRCAM Neuronal Cell Adhesion Molecule

FAT1 FAT Atypical Cadherin 1

HLA-DRB5 Major Histocompatibility Complex, Class II, DR Beta 5

HLA-DPB1 Major Histocompatibility Complex, Class II, DP Beta 1

PLA2R1 Phospholipase A2 Receptor 1

HLA-DOA Major Histocompatibility Complex, Class II, DO Alpha

ODZ1 Teneurin Transmembrane Protein 1

HLA-DOB Major Histocompatibility Complex, Class II, DO Beta

FLRT3 Fibronectin Leucine Rich Transmembrane Protein 3

MYO6 Myosin VI

MPDZ Multiple PDZ Domain Protein

PCDHB3 Protocadherin Beta 3

MFI2 Antigen P97 (Melanoma Associated) AXL AXL Receptor Tyrosine Kinase

HLA-DQA2 Major Histocompatibility Complex, Class II, DQ Alpha 2

FLNA Filamin A, Alpha

HLA-DQA1 Major Histocompatibility Complex, Class II, DQ Alpha 1

P2RX5 Purinergic Receptor P2X, Ligand Gated Ion Channel, 5

NCAM2 Neural Cell Adhesion Molecule 2

CDH13 Cadherin 13

CHRM3 Cholinergic Receptor, Muscarinic 3

CXORF61 Cancer/Testis Antigen 83

HLA-DPA1 Major Histocompatibility Complex, Class II, DP Alpha 1

SGCE Sarcoglycan, Epsilon

TM4SF1 Transmembrane 4 L Six Family Member 1

SLC14A1

Solute Carrier Family 14 (Urea Transporter), Member 1 (Kidd Blood Group)

DST Dystonin

EMP1 Epithelial Membrane Protein 1

HLA-DRA

GO:0005887 **Integral To Plasma Membrane**

FLRT3 Fibronectin Leucine Rich Transmembrane Protein 3

SLC5A3 Solute Carrier Family 5 (Sodium/Myo-Inositol Cotransporter), Member 3

GYPE Glycophorin E (MNS Blood Group)

HLA-DRB3 Major Histocompatibility Complex, Class II, DR Beta 3

PCDHB3 Protocadherin Beta 3

MFI2 Antigen P97 (Melanoma Associated) LPAR4 Lysophosphatidic Acid Receptor 4

AXL AXL Receptor Tyrosine Kinase

GPRC5A G Protein-Coupled Receptor, Class C, Group 5, Member A HLA-DQA2 Major Histocompatibility Complex, Class II, DQ Alpha 2

HLA-DQA1 Major Histocompatibility Complex, Class II, DQ Alpha 1

NRCAM Neuronal Cell Adhesion Molecule

P2RX5 Purinergic Receptor P2X, Ligand Gated Ion Channel, 5

CHRM3 Cholinergic Receptor, Muscarinic 3

FAT1 FAT Atypical Cadherin 1

HLA-DPA1 Major Histocompatibility Complex, Class II, DP Alpha 1

SGCE Sarcoglycan, Epsilon

TM4SF1 Transmembrane 4 L Six Family Member 1

SLC14A1

Solute Carrier Family 14 (Urea Transporter), Member 1 (Kidd Blood Group)

PLA2R1 Phospholipase A2 Receptor 1

ODZ1 Teneurin Transmembrane Protein 1

HLA-DRA Major Histocompatibility Complex, Class II, DR Alpha

GO:0031226 **Intrinsic To Plasma Membrane**

FLRT3 Fibronectin Leucine Rich Transmembrane Protein 3

SLC5A3 Solute Carrier Family 5 (Sodium/Myo-Inositol Cotransporter), Member 3

GYPE Glycophorin E (MNS Blood Group)

HLA-DRB3 Major Histocompatibility Complex, Class II, DR Beta 3

PCDHB3 Protocadherin Beta 3

MFI2 Antigen P97 (Melanoma Associated) LPAR4 Lysophosphatidic Acid Receptor 4

AXL AXL Receptor Tyrosine Kinase

GPRC5A G Protein-Coupled Receptor, Class C, Group 5, Member A HLA-DQA2 Major Histocompatibility Complex, Class II, DQ Alpha 2

HLA-DQA1 Major Histocompatibility Complex, Class II, DQ Alpha 1

NRCAM Neuronal Cell Adhesion Molecule

P2RX5 Purinergic Receptor P2X, Ligand Gated Ion Channel, 5

CHRM3 Cholinergic Receptor, Muscarinic 3

FAT1 FAT Atypical Cadherin 1

HLA-DPA1 Major Histocompatibility Complex, Class II, DP Alpha 1

SGCE Sarcoglycan, Epsilon

TM4SF1 Transmembrane 4 L Six Family Member 1

SLC14A1

Solute Carrier Family 14 (Urea Transporter), Member 1 (Kidd Blood Group)

PLA2R1 Phospholipase A2 Receptor 1

ODZ1 Teneurin Transmembrane Protein 1

HLA-DRA Major Histocompatibility Complex, Class II, DR Alpha

GO:0016021 **Integral To Membrane**

HLA-DQB1 Major Histocompatibility Complex, Class II, DQ Beta 1

SLC5A3 Solute Carrier Family 5 (Sodium/Myo-Inositol Cotransporter), Member 3

DCC DCC Netrin 1 Receptor

GYPE Glycophorin E (MNS Blood Group)

HLA-DRB3 Major Histocompatibility Complex, Class II, DR Beta 3

PTPLAD2 protein tyrosine phosphatase-like protein PTPLAD2

APH1B APH1B Gamma Secretase Subunit

LPAR4 Lysophosphatidic Acid Receptor 4

HLA-DMB Major Histocompatibility Complex, Class II, DM Beta GPRC5A G Protein-Coupled Receptor, Class C, Group 5, Member A HLA-DMA Major Histocompatibility Complex, Class II, DM Alpha

CD74 CD74 Molecule, Major Histocompatibility Complex, Class II Invariant Chain

IL31RA Interleukin 31 Receptor A

NRCAM Neuronal Cell Adhesion Molecule

TMEM108 Transmembrane Protein 108

FAT1 FAT Atypical Cadherin 1

SLC4A8 Solute Carrier Family 4, Sodium Bicarbonate Cotransporter, Member 8

HLA-DRB5 Major Histocompatibility Complex, Class II, DR Beta 5

NALCN Sodium Leak Channel, Non Selective

UNC5C Unc-5 Homolog C (C. Elegans)

HLA-DPB1 Major Histocompatibility Complex, Class II, DP Beta 1

PLA2R1 Phospholipase A2 Receptor 1

HLA-DOA Major Histocompatibility Complex, Class II, DO Alpha

SLCO5A1 Solute Carrier Organic Anion Transporter Family, Member 5A1

ODZ1 Teneurin Transmembrane Protein 1

HLA-DOB Major Histocompatibility Complex, Class II, DO Beta

FLRT3 Fibronectin Leucine Rich Transmembrane Protein 3

PCDHB3 Protocadherin Beta 3

MFI2 Antigen P97 (Melanoma Associated) MXRA7 Matrix-Remodelling Associated 7

AXL AXL Receptor Tyrosine Kinase

EDA2R Ectodysplasin A2 Receptor

HLA-DQA2 Major Histocompatibility Complex, Class II, DQ Alpha 2

HLA-DQA1 Major Histocompatibility Complex, Class II, DQ Alpha 1

P2RX5 Purinergic Receptor P2X, Ligand Gated Ion Channel, 5

NCAM2 Neural Cell Adhesion Molecule 2

CHRM3 Cholinergic Receptor, Muscarinic 3

PLEKHH2 Pleckstrin Homology Domain Containing, Family H (With MyTH4 Domain) Member 2

CXORF61 Cancer/Testis Antigen 83

HLA-DPA1 Major Histocompatibility Complex, Class II, DP Alpha 1

SGCE Sarcoglycan, Epsilon

TM4SF1 Transmembrane 4 L Six Family Member 1

SLC14A1

Solute Carrier Family 14 (Urea Transporter), Member 1 (Kidd Blood Group)

SLITRK6 SLIT And NTRK-Like Family, Member 6

EMP1 Epithelial Membrane Protein 1

HLA-DRA Major Histocompatibility Complex, Class II, DR Alpha

GO:0031224 **Intrinsic To Membrane**

HLA-DQB1 Major Histocompatibility Complex, Class II, DQ Beta 1

SLC5A3 Solute Carrier Family 5 (Sodium/Myo-Inositol Cotransporter), Member 3

DCC DCC Netrin 1 Receptor

GYPE Glycophorin E (MNS Blood Group)

HLA-DRB3 Major Histocompatibility Complex, Class II, DR Beta 3

PTPLAD2 protein tyrosine phosphatase-like protein PTPLAD2

APH1B APH1B Gamma Secretase Subunit

LPAR4 Lysophosphatidic Acid Receptor 4

HLA-DMB Major Histocompatibility Complex, Class II, DM Beta GPRC5A G Protein-Coupled Receptor, Class C, Group 5, Member A HLA-DMA Major Histocompatibility Complex, Class II, DM Alpha

CD74 CD74 Molecule, Major Histocompatibility Complex, Class II Invariant Chain

IL31RA Interleukin 31 Receptor A

NRCAM Neuronal Cell Adhesion Molecule

TMEM108 Transmembrane Protein 108

FAT1 FAT Atypical Cadherin 1

SLC4A8 Solute Carrier Family 4, Sodium Bicarbonate Cotransporter, Member 8

HLA-DRB5 Major Histocompatibility Complex, Class II, DR Beta 5

NALCN Sodium Leak Channel, Non Selective

UNC5C Unc-5 Homolog C (C. Elegans)

HLA-DPB1 Major Histocompatibility Complex, Class II, DP Beta 1

PLA2R1 Phospholipase A2 Receptor 1

HLA-DOA Major Histocompatibility Complex, Class II, DO Alpha

SLCO5A1 Solute Carrier Organic Anion Transporter Family, Member 5A1

ODZ1 Teneurin Transmembrane Protein 1

HLA-DOB Major Histocompatibility Complex, Class II, DO Beta

FLRT3 Fibronectin Leucine Rich Transmembrane Protein 3

PCDHB3 Protocadherin Beta 3

MFI2 Antigen P97 (Melanoma Associated) MXRA7 Matrix-Remodelling Associated 7

AXL AXL Receptor Tyrosine Kinase

EDA2R Ectodysplasin A2 Receptor

HLA-DQA2 Major Histocompatibility Complex, Class II, DQ Alpha 2

HLA-DQA1 Major Histocompatibility Complex, Class II, DQ Alpha 1

P2RX5 Purinergic Receptor P2X, Ligand Gated Ion Channel, 5

NCAM2 Neural Cell Adhesion Molecule 2

CDH13 Cadherin 13

CHRM3 Cholinergic Receptor, Muscarinic 3

PLEKHH2

Pleckstrin Homology Domain Containing, Family H (With MyTH4 Domain) Member 2

CXORF61 Cancer/Testis Antigen 83

HLA-DPA1 Major Histocompatibility Complex, Class II, DP Alpha 1

SGCE Sarcoglycan, Epsilon

TM4SF1 Transmembrane 4 L Six Family Member 1

SLC14A1

Solute Carrier Family 14 (Urea Transporter), Member 1 (Kidd Blood Group)

SLITRK6 SLIT And NTRK-Like Family, Member 6

EMP1 Epithelial Membrane Protein 1

HLA-DRA Major Histocompatibility Complex, Class II, DR Alpha

GO:0006355 **Regulation Of Transcription, DNA-Dependent**

ZNF808 Zinc Finger Protein 808

ZNF85 Zinc Finger Protein 85

ZNF582 Zinc Finger Protein 582

ZNF83 Zinc Finger Protein 83

ZNF583 Zinc Finger Protein 583

ZNF558 Zinc Finger Protein 558

HDX Highly Divergent Homeobox

HOXD10 Homeobox D10

IL31RA Interleukin 31 Receptor A ZNF709 Zinc Finger Protein 709

ZFP90 ZFP90 Zinc Finger Protein

ZNF737 Zinc Finger Protein 737

ZNF404 Zinc Finger Protein 404

ZNF286A Zinc Finger Protein 286A MEIS3P1 Meis Homeobox 3 Pseudogene 1

RUNX1 Runt-Related Transcription Factor 1

ZNF420 Zinc Finger Protein 420

ZNF607 Zinc Finger Protein 607

ZNF724P Zinc Finger Protein 724, Pseudogene

ALX1 ALX Homeobox 1

ZNF528 Zinc Finger Protein 528

MYO6 Myosin VI

ZNF542 Zinc Finger Protein 542

ZNF284 Zinc Finger Protein 284

ZFP30 ZFP30 Zinc Finger Protein

ZNF790 Zinc Finger Protein 90

ZNF221 Zinc Finger Protein 221

ZFP28 ZFP28 Zinc Finger Protein

INHBA Inhibin, Beta A

ZNF197 Zinc Finger Protein 197

TGIF1 TGFB-Induced Factor Homeobox 1

TFAP2A

Transcription Factor AP-2 Alpha (Activating Enhancer Binding Protein 2 Alpha)

ZSCAN16 Zinc Finger And SCAN Domain Containing 16

ZNF571 Zinc Finger Protein 571

ZNF573 Zinc Finger Protein 573

GO:0045449 **Regulation Of Transcription**

ZNF808 Zinc Finger Protein 808

ZNF85 Zinc Finger Protein 85

ZNF582 Zinc Finger Protein 582

ZNF83 Zinc Finger Protein 83

ZNF583 Zinc Finger Protein 583

ZNF558 Zinc Finger Protein 558

ZNF781 Zinc Finger Protein 781

HDX Highly Divergent Homeobox

HOXD10 Homeobox D10

IL31RA Interleukin 31 Receptor A ZNF709 Zinc Finger Protein 709

ZFP90 ZFP90 Zinc Finger Protein

ZNF737 Zinc Finger Protein 737

ZNF404 Zinc Finger Protein 404

ZNF329 Zinc Finger Protein 329

ZNF286A Zinc Finger Protein 286A MEIS3P1 Meis Homeobox 3 Pseudogene 1

ZNF607 Zinc Finger Protein 607

RUNX1 Runt-Related Transcription Factor 1

ZNF420 Zinc Finger Protein 420

ZNF724P Zinc Finger Protein 724, Pseudogene

ALX1 ALX Homeobox 1

ZNF528 Zinc Finger Protein 528

KHDRBS3 KH Domain Containing, RNA Binding, Signal Transduction Associated 3

MYO6 Myosin VI

ZNF542 Zinc Finger Protein 542

ZNF284 Zinc Finger Protein 284

SNAPC1 Small Nuclear RNA Activating Complex, Polypeptide 1, 43kDa

ZFP30 ZFP30 Zinc Finger Protein

ZNF790 Zinc Finger Protein 790

EDA2R Ectodysplasin A2 Receptor

ZNF521 Zinc Finger Protein 521

ZNF221 Zinc Finger Protein 221

ZFP28 ZFP28 Zinc Finger Protein

FLNA Filamin A, Alpha

INHBA Inhibin, Beta A

ZNF197 Zinc Finger Protein 197

BTG1 B-Cell Translocation Gene 1, Anti-Proliferative

TGIF1 TGFB-Induced Factor Homeobox 1

RIPK2 Receptor-Interacting Serine-Threonine Kinase 2

TFAP2A

Transcription Factor AP-2 Alpha (Activating Enhancer Binding Protein 2 Alpha)

ZSCAN16 Zinc Finger And SCAN Domain Containing 16

ZNF571 Zinc Finger Protein 571

ZNF573 Zinc Finger Protein 573

GO:0051252 **Regulation Of RNA Metabolic Process**

ZNF808 Zinc Finger Protein 808

ZNF85 Zinc Finger Protein 85

ZNF582 Zinc Finger Protein 582

ZNF83 Zinc Finger Protein 83

ZNF583 Zinc Finger Protein 538

ZNF558 Zinc Finger Protein 558

HDX Highly Divergent Homeobox

HOXD10 Homeobox D10

IL31RA Interleukin 31 Receptor A ZNF709 Zinc Finger Protein 709

ZFP90 ZFP90 Zinc Finger Protein

ZNF737 Zinc Finger Protein 737

ZNF404 Zinc Finger Protein 404

ZNF286A Zinc Finger Protein 286A MEIS3P1 Meis Homeobox 3 Pseudogene 1

RUNX1 Runt-Related Transcription Factor 1

ZNF420 Zinc Finger Protein 420

ZNF607 Zinc Finger Protein 607

ZNF724P Zinc Finger Protein 724, Pseudogene

ALX1 ALX Homeobox 1

ZNF528 Zinc Finger Protein 528

MYO6 Myosin VI

ZNF542 Zinc Finger Protein 542

ZNF284 Zinc Finger Protein 284

ZFP30 ZFP30 Zinc Finger Protein

ZNF790 Zinc Finger Protein 790

ZNF221 Zinc Finger Protein 221

ZFP28 ZFP28 Zinc Finger Protein

INHBA Inhibin, Beta A

ZNF197 Zinc Finger Protein 197

TGIF1 TGFB-Induced Factor Homeobox 1

TFAP2A

Transcription Factor AP-2 Alpha (Activating Enhancer Binding Protein 2 Alpha)

ZSCAN16 Zinc Finger And SCAN Domain Containing 16

ZNF571 Zinc Finger Protein 571

ZNF573 Zinc Finger Protein 573

GO:0003677 **DNA Binding**

ZNF808 Zinc Finger Protein 808

ZNF85 Zinc Finger Protein 85

ZNF582 Zinc Finger Protein 582

ZNF83 Zinc Finger Protein 83

ZNF583 Zinc Finger Protein 538

ZNF558 Zinc Finger Protein 558

ZNF781 Zinc Finger Protein 781

HDX Highly Divergent Homeobox

HOXD10 Homeobox D10

ZNF709 Zinc Finger Protein 709

ZFP90 ZFP90 Zinc Finger Protein

ZNF737 Zinc Finger Protein 737

ZNF404 Zinc Finger Protein 404

ZNF329 Zinc Finger Protein 329

ZNF286A Zinc Finger Protein 286A MEIS3P1 Meis Homeobox 3 Pseudogene 1

RUNX1 Runt-Related Transcription Factor 1

ZNF420 Zinc Finger Protein 420

ZNF607 Zinc Finger Protein 607

ZNF724P Zinc Finger Protein 724, Pseudogene

ALX1 ALX Homeobox 1

ZNF528 Zinc Finger Protein 528

ZNF542 Zinc Finger Protein 542

ZNF284 Zinc Finger Protein 284

SNAPC1 Small Nuclear RNA Activating Complex, Polypeptide 1, 43kDa

ZFP30 ZFP30 Zinc Finger Protein

ZNF790 Zinc Finger Protein 790

ZNF521 Zinc Finger Protein 521

ZNF221 Zinc Finger Protein 221

ZFP28 ZFP28 Zinc Finger Protein

ZNF197 Zinc Finger Protein 197

TGIF1 TGFB-Induced Factor Homeobox 1

TFAP2A

Transcription Factor AP-2 Alpha (Activating Enhancer Binding Protein 2 Alpha)

ZSCAN16 Zinc Finger And SCAN Domain Containing 16

ZNF571 Zinc Finger Protein 571

ZNF573 Zinc Finger Protein 573

GO:0006350 **Transcription**

ZNF808 Zinc Finger Protein 808

ZNF85 Zinc Finger Protein 85

ZNF582 Zinc Finger Protein 582

ZNF83 Zinc Finger Protein 83

ZNF583 Zinc Finger Protein 538

ZNF558 Zinc Finger Protein 558

ZNF781 Zinc Finger Protein 781

HOXD10 Highly Divergent Homeobox

ZNF709 Zinc Finger Protein 709

ZFP90 ZFP90 Zinc Finger Protein

ZNF404 Zinc Finger Protein 404

ZNF329 Zinc Finger Protein 329

ZNF286A Zinc Finger Protein 286A

RUNX1 Runt-Related Transcription Factor 1

ZNF420 Zinc Finger Protein 420

ZNF607 Zinc Finger Protein 607

ALX1 ALX Homeobox 1

ZNF528 Zinc Finger Protein 528

KHDRBS3 KH Domain Containing, RNA Binding, Signal Transduction Associated 3

ZNF542 Zinc Finger Protein 542

ZNF284 Zinc Finger Protein 284

SNAPC1 Small Nuclear RNA Activating Complex, Polypeptide 1, 43kDa

ZFP30 ZFP30 Zinc Finger Protein

ZNF790 Zinc Finger Protein 790

ZNF521 Zinc Finger Protein 521

ZNF221 Zinc Finger Protein 221

ZFP28 ZFP28 Zinc Finger Protein

ZNF197 Zinc Finger Protein 197

TGIF1 TGFB-Induced Factor Homeobox 1

TFAP2A

Transcription Factor AP-2 Alpha (Activating Enhancer Binding Protein 2 Alpha)

ZSCAN16 Zinc Finger And SCAN Domain Containing 16

ZNF571 Zinc Finger Protein 571

ZNF573 Zinc Finger Protein 573

GO:0008270 **Zinc Ion Binding**

ZNF808 Zinc Finger Protein 808

ZNF85 Zinc Finger Protein 85

ZNF582 Zinc Finger Protein 582

ZNF83 Zinc Finger Protein 83

RNF17 Ring Finger Protein 17

ZNF583 Zinc Finger Protein 583

ZNF558 Zinc Finger Protein 558

ZNF781 Zinc Finger Protein 781

SOBP Sine Oculis Binding Protein Homolog (Drosophila) ZNF709 Zinc Finger Protein 709

ZFP90 ZFP90 Zinc Finger Protein

ZNF737 Zinc Finger Protein 737

ZNF404 Zinc Finger Protein 404

ZNF329 Zinc Finger Protein 329

ZNF286A Zinc Finger Protein 286A ZNF420 Zinc Finger Protein 420

ZNF607 Zinc Finger Protein 607

ZNF724P Zinc Finger Protein 724, Pseudogene

DCTD DCMP Deaminase

ZNF528 Zinc Finger Protein 528

ADARB1 Adenosine Deaminase, RNA-Specific, B1

ZNF542 Zinc Finger Protein 542

ZNF284 Zinc Finger Protein 284

ZFP30 ZFP30 Zinc Finger Protein

MFI2 Antigen P97 (Melanoma Associated) ZNF790 Zinc Finger Protein 790

ZNF521 Zinc Finger Protein 521

ZNF221 Zinc Finger Protein 221

ZFP28 ZFP28 Zinc Finger Protein

ZNF197 Zinc Finger Protein 197

CA8 Carbonic Anhydrase VIII

ZSCAN16 Zinc Finger And SCAN Domain Containing 16

ZNF571 Zinc Finger Protein 571

ZNF573 Zinc Finger Protein 573

GO:0042825 **TAP Complex**

HLA-DMB Major Histocompatibility Complex, Class II, DM Beta HLA-DOB Major Histocompatibility Complex, Class II, DO Beta HLA-DMA Major Histocompatibility Complex, Class II, DM Alpha HLA-DRA Major Histocompatibility Complex, Class II, DR Alpha

GO:0042824 **MHC Class I Peptide Loading Complex**

HLA-DMB Major Histocompatibility Complex, Class II, DM Beta HLA-DOB Major Histocompatibility Complex, Class II, DO Beta HLA-DMA Major Histocompatibility Complex, Class II, DM Alpha HLA-DRA Major Histocompatibility Complex, Class II, DR Alpha

GO:0002495 **Antigen Processing And Presentation Of Peptide Antigen Via MHC Class II**

HLA-DOA Major Histocompatibility Complex, Class II, DO Alpha

HLA-DMA Major Histocompatibility Complex, Class II, DM Alpha

CD74 CD74 Molecule, Major Histocompatibility Complex, Class II Invariant Chain

HLA-DRA Major Histocompatibility Complex, Class II, DR Alpha

GO:0019886 **Antigen Processing And Presentation Of Exogenous Peptide Antigen Via MHC Class II**

HLA-DOA Major Histocompatibility Complex, Class II, DO Alpha

HLA-DMA Major Histocompatibility Complex, Class II, DM Alpha

CD74 CD74 Molecule, Major Histocompatibility Complex, Class II Invariant Chain

HLA-DRA Major Histocompatibility Complex, Class II, DR Alpha

GO:0042287 **MHC Protein Binding**

HLA-DMB Major Histocompatibility Complex, Class II, DM Beta HLA-DOB Major Histocompatibility Complex, Class II, DO Beta HLA-DMA Major Histocompatibility Complex, Class II, DM Alpha

CD74 CD74 Molecule, Major Histocompatibility Complex, Class II Invariant Chain

HLA-DRA Major Histocompatibility Complex, Class II, DR Alpha

GO:0002478 **Antigen Processing And Presentation Of Exogenous Peptide Antigen**

HLA-DOA Major Histocompatibility Complex, Class II, DO Alpha

HLA-DMA Major Histocompatibility Complex, Class II, DM Alpha

CD74 CD74 Molecule, Major Histocompatibility Complex, Class II Invariant Chain

HLA-DRA Major Histocompatibility Complex, Class II, DR Alpha

GO:0019884 **Antigen Processing And Presentation Of Exogenous Antigen** HLA-DOA Major Histocompatibility Complex, Class II, DO Alpha HLA-DMA Major Histocompatibility Complex, Class II, DM Alpha

CD74 CD74 Molecule, Major Histocompatibility Complex, Class II Invariant Chain

HLA-DRA Major Histocompatibility Complex, Class II, DR Alpha

GO:0042288 **MHC class I protein binding**

HLA-DMB Major Histocompatibility Complex, Class II, DM Beta HLA-DOB Major Histocompatibility Complex, Class II, DO Beta HLA-DMA Major Histocompatibility Complex, Class II, DM Alpha HLA-DRA Major Histocompatibility Complex, Class II, DR Alpha

GO:0048002 **Antigen Processing And Presentation Of Peptide Antigen**

HLA-DOA Major Histocompatibility Complex, Class II, DO Alpha

HLA-DMA Major Histocompatibility Complex, Class II, DM Alpha

CD74 CD74 Molecule, Major Histocompatibility Complex, Class II Invariant Chain

HLA-DRA Major Histocompatibility Complex, Class II, DR Alpha

GO:0000323 **Lytic Vacuole**

CTSZ Cathepsin Z

HLA-DMB Major Histocompatibility Complex, Class II, DM Beta HLA-DOB Major Histocompatibility Complex, Class II, DO Beta HLA-DMA Major Histocompatibility Complex, Class II, DM Alpha

CD74 CD74 Molecule, Major Histocompatibility Complex, Class II Invariant Chain

HLA-DRA Major Histocompatibility Complex, Class II, DR Alpha

GO:0005764 **Lysosome**

CTSZ Cathepsin Z

HLA-DMB Major Histocompatibility Complex, Class II, DM Beta HLA-DOB Major Histocompatibility Complex, Class II, DO Beta HLA-DMA Major Histocompatibility Complex, Class II, DM Alpha

CD74 CD74 Molecule, Major Histocompatibility Complex, Class II Invariant Chain

HLA-DRA Major Histocompatibility Complex, Class II, DR Alpha

GO:0030030 **Cell Projection Organization**

DCC DCC Netrin 1 Receptor

DNM3 Dynamin 3

MYO6 Myosin VI

KIF5C Kinesin Family Member 5C

PTEN Phosphatase And Tensin Homolog NRCAM Neuronal Cell Adhesion Molecule CDH13 Cadherin 13

NCAM2 Neural Cell Adhesion Molecule 2

CAPG Capping Protein (Actin Filament), Gelsolin-Like

UNC5C Unc-5 Homolog C (C. Elegans) SLITRK6 SLIT And NTRK-Like Family, Member 6

DST Dystonin

PTENP1 Phosphatase And Tensin Homolog Pseudogene 1 (Functional) GO:0043005 **Neuron Projection**

NRCAM Neuronal Cell Adhesion Molecule

DCC DCC Netrin 1 Receptor

DNM3 Dynamin 3

NCAM2 Neural Cell Adhesion Molecule 2

CDH13 Cadherin 13

MYO6 Myosin VI

CHRM3 Cholinergic Receptor, Muscarinic 3

MPDZ Multiple PDZ Domain Protein

KIF5C Kinesin Family Member 5C GO:0031175 **Neuron Projection Development**

NRCAM Neuronal Cell Adhesion Molecule

DCC DCC Netrin 1 Receptor

NCAM2 Neural Cell Adhesion Molecule 2

MYO6 Myosin VI

KIF5C Kinesin Family Member 5C UNC5C Unc-5 Homolog C (C. Elegans) SLITRK6 SLIT And NTRK-Like Family, Member 6

DST Dystonin

PTEN Phosphatase And Tensin Homolog

PTENP1 Phosphatase And Tensin Homolog Pseudogene 1 (Functional) GO:0048666 **Neuron Development**

NRCAM Neuronal Cell Adhesion Molecule

DCC DCC Netrin 1 Receptor

NCAM2 Neural Cell Adhesion Molecule 2

MYO6 Myosin VI

KIF5C Kinesin Family Member 5C UNC5C Unc-5 Homolog C (C. Elegans) SLITRK6 SLIT And NTRK-Like Family, Member 6

DST Dystonin

PTEN Phosphatase And Tensin Homolog

HOXD10 Homeobox D10

PTENP1 Phosphatase And Tensin Homolog Pseudogene 1 (Functional) GO:0007409 **Axonogenesis**

NRCAM Neuronal Cell Adhesion Molecule

DCC DCC Netrin 1 Receptor

NCAM2 Neural Cell Adhesion Molecule 2

KIF5C Kinesin Family Member 5C UNC5C Unc-5 Homolog C (C. Elegans) SLITRK6 SLIT And NTRK-Like Family, Member 6

DST Dystonin

GO:0048667 **Cell Morphogenesis Involved In Neuron Differentiation**

NRCAM Neuronal Cell Adhesion Molecule

DCC DCC Netrin 1 Receptor

NCAM2 Neural Cell Adhesion Molecule 2

KIF5C Kinesin Family Member 5C UNC5C Unc-5 Homolog C (C. Elegans) SLITRK6 SLIT And NTRK-Like Family, Member 6

DST Dystonin

GO:0048812 **Neuron Projection Morphogenesis**

NRCAM Neuronal Cell Adhesion Molecule

DCC DCC Netrin 1 Receptor

NCAM2 Neural Cell Adhesion Molecule 2

KIF5C Kinesin Family Member 5C UNC5C Unc-5 Homolog C (C. Elegans) SLITRK6 SLIT And NTRK-Like Family, Member 6

DST Dystonin

GO:0007416 **Synaptogenesis**

NRCAM Neuronal Cell Adhesion Molecule

DNM3 Dynamin 3

MYO6 Myosin VI

PCDHB3 Protocadherin Beta 3

GO:0002495 **Antigen Processing And Presentation Of Peptide Antigen Via MHC Class II**

HLA-DOA Major Histocompatibility Complex, Class II, DO Alpha

HLA-DMA Major Histocompatibility Complex, Class II, DM Alpha

CD74 CD74 Molecule, Major Histocompatibility Complex, Class II Invariant Chain

HLA-DRA Major Histocompatibility Complex, Class II, DR Alpha

GO:0019886 **Antigen Processing And Presentation Of Exogenous Peptide Antigen Via MHC Class II**

HLA-DOA Major Histocompatibility Complex, Class II, DO Alpha

HLA-DMA Major Histocompatibility Complex, Class II, DM Alpha

CD74 CD74 Molecule, Major Histocompatibility Complex, Class II Invariant Chain

HLA-DRA Major Histocompatibility Complex, Class II, DR Alpha

GO:0002478 **Antigen Processing And Presentation Of Exogenous Peptide Antigen**

HLA-DOA Major Histocompatibility Complex, Class II, DO Alpha

HLA-DMA Major Histocompatibility Complex, Class II, DM Alpha

CD74 CD74 Molecule, Major Histocompatibility Complex, Class II Invariant Chain

HLA-DRA Major Histocompatibility Complex, Class II, DR Alpha

GO:0019884 **Antigen Processing And Presentation Of Exogenous Antigen** HLA-DOA Major Histocompatibility Complex, Class II, DO Alpha HLA-DMA Major Histocompatibility Complex, Class II, DM Alpha

CD74 CD74 Molecule, Major Histocompatibility Complex, Class II Invariant Chain

HLA-DRA Major Histocompatibility Complex, Class II, DR Alpha

GO:0048002 **Antigen Processing And Presentation Of Peptide Antigen**

HLA-DOA Major Histocompatibility Complex, Class II, DO Alpha

HLA-DMA Major Histocompatibility Complex, Class II, DM Alpha

CD74 CD74 Molecule, Major Histocompatibility Complex, Class II Invariant Chain

HLA-DRA Major Histocompatibility Complex, Class II, DR Alpha

**Downregulated G10 vs Control**

GO:0042995 **Cell Projection**

MTSS1 Metastasis Suppressor 1

PDGFA Platelet-Derived Growth Factor Alpha Polypeptide

FSCN1 Fascin Actin-Bundling Protein 1

CSPG4 Chondroitin Sulfate Proteoglycan 4

TAC1 Tachykinin, Precursor 1

RDX Radixin

SDC3 Syndecan 3

NCAM1 Neural Cell Adhesion Molecule 1

PROM1 Prominin 1

CTTNBP2 Cortactin Binding Protein 2

SEMA6A Semaphorin 6A

SLC1A3

Solute Carrier Family 1 (Glial High Affinity Glutamate Transporter), Member 3

TIAM2 T-Cell Lymphoma Invasion And Metastasis 2

BACE1 Beta-Site APP-Cleaving Enzyme 1

NEFH Neurofilament, Heavy Polypeptide

ROBO2 Roundabout, Axon Guidance Receptor, Homolog 2 (Drosophila) LRP2 Low Density Lipoprotein Receptor-Related Protein 2

CACNA1C Calcium Channel, Voltage-Dependent, L Type, Alpha 1C Subunit

GO:0030424 **Axon**

NCAM1 Neural Cell Adhesion Molecule 1

SEMA6A Semaphorin 6A

BACE1 Beta-Site APP-Cleaving Enzyme 1

NEFH Neurofilament, Heavy Polypeptide

TAC1 Tachykinin, Precursor 1

ROBO2 Roundabout, Axon Guidance Receptor, Homolog 2 (Drosophila) SDC3 Syndecan 3

GO:0043005 **Neuron Projection**

NCAM1 Neural Cell Adhesion Molecule 1

SEMA6A Semaphorin 6A

SLC1A3

Solute Carrier Family 1 (Glial High Affinity Glutamate Transporter), Member 3

TIAM2 T-Cell Lymphoma Invasion And Metastasis 2

BACE1 Beta-Site APP-Cleaving Enzyme 1

NEFH Neurofilament, Heavy Polypeptide

TAC1 Tachykinin, Precursor 1

ROBO2 Roundabout, Axon Guidance Receptor, Homolog 2 (Drosophila) CACNA1C Calcium Channel, Voltage-Dependent, L Type, Alpha 1C Subunit SDC3 Syndecan 3

GO:0000904 **Cell Morphogenesis Involved In Differentiation**

SLITRK2 SLIT And NTRK-Like Family, Member 2

SEMA5A Semaphorin 5A

SLITRK1 SLIT And NTRK-Like Family, Member 1

SEMA6A Semaphorin 6A

SLC1A3

Solute Carrier Family 1 (Glial High Affinity Glutamate Transporter), Member 3

EFNA5 Ephrin-A5

ROBO2 Roundabout, Axon Guidance Receptor, Homolog 2 (Drosophila) EPHB2 EPH Receptor B2

FN1 Fibronectin 1

GO:0048667 **Cell Morphogenesis Involved In Neuron Differentiation**

SLITRK2 SLIT And NTRK-Like Family, Member 2

SEMA5A Semaphorin 5A

SLITRK1 SLIT And NTRK-Like Family, Member 1

SEMA6A Semaphorin 6A

SLC1A3

Solute Carrier Family 1 (Glial High Affinity Glutamate Transporter), Member 3

EFNA5 Ephrin-A5

ROBO2 Roundabout, Axon Guidance Receptor, Homolog 2 (Drosophila) EPHB2 EPH Receptor B2

hsa04360 **Axon Guidance**

SEMA5A Semaphorin 5A SEMA6A Semaphorin 6A PLXNA2 Plexin A2

EFNB2 Ephrin-B2

EFNA5 Ephrin-A5

ROBO2 Roundabout, Axon Guidance Receptor, Homolog 2 (Drosophila) EPHB2 EPH Receptor B2

GO:0048666 **Neuron Development**

SLITRK2 SLIT And NTRK-Like Family, Member 2

LIF Leukemia Inhibitory Factor

SEMA5A Semaphorin 5A

SLITRK1 SLIT And NTRK-Like Family, Member 1

SEMA6A Semaphorin 6A

SLC1A3

EFNA5

Solute Carrier Family 1 (Glial High Affinity Glutamate Transporter), Member 3

Ephrin-A5

ROBO2 Roundabout, Axon Guidance Receptor, Homolog 2 (Drosophila)

LHX8 LIM Homeobox 8

EPHB2 EPH Receptor B2 hsa04810 **Regulation Of Actin Cytoskeleton**

TIAM2 T-Cell Lymphoma Invasion And Metastasis 2

TIAM1 T-Cell Lymphoma Invasion And Metastasis 1

PDGFA Platelet-Derived Growth Factor Alpha Polypeptide

DIAPH3 Diaphanous-Related Formin 3

ITGA11 Integrin, Alpha 11

IQGAP2 IQ Motif Containing GTPase Activating Protein 2

ITGA10 Integrin, Alpha 10

TMSB4X Thymosin-beta-4 X Chromosome

RDX Radixin

FN1 Fibronectin 1

GO:0006928 **Cell Motion**

MTSS1 Metastasis Suppressor 1

PLXNA2 Plexin A2

ITGA11 Integrin, Alpha 11

KITLG KIT Ligand

EPHB2 EPH Receptor B2

SEMA5A Semaphorin 5A SEMA6A Semaphorin 6A

CTTNBP2 Cortactin Binding Protein 2

ROBO2 Roundabout, Axon Guidance Receptor, Homolog 2 (Drosophila) EFNA5 Ephrin-A5

PPAP2B Phosphatidic Acid Phosphatase Type 2B TWIST1 Twist Family BHLH Transcription Factor 1

FN1 Fibronectin 1

GO:0003779 **Actin Binding**

CORO2B Coronin, Actin Binding Protein, 2B DIXDC1 DIX Domain Containing 1

MTSS1 Metastasis Suppressor 1

MYO10 Myosin X

DIAPH3 Diaphanous-Related Formin 3

ENC1 Ectodermal-Neural Cortex 1 (With BTB Domain) FSCN1 Fascin Actin-Bundling Protein 1

IQGAP2 IQ Motif Containing GTPase Activating Protein 2

TMSB4X Thymosin-beta-4 X Chromosome

RDX Radixin

**Downregulated G10 vs A7**

GO:0042995 **Cell Projection**

MTSS1 Metastasis Suppressor 1

TRPM6 Transient Receptor Potential Cation Channel, Subfamily M, Member 6

SWAP70 SWAP Switching B-Cell Complex 70kDa Subunit

FSCN1 Fascin Actin-Bundling Protein 1

UCHL1 Ubiquitin Carboxyl-Terminal Esterase L1 (Ubiquitin Thiolesterase) CSPG4 Chondroitin Sulfate Proteoglycan 4

RDX Radixin

APBB1IP Amyloid Beta (A4) Precursor Protein-Binding, Family B, Member 1 (Fe65) SDC3 Syndecan 3

NCAM1 Neural Cell Adhesion Molecule 1

PROM1 Prominin 1

PCSK1 Proprotein Convertase Subtilisin/Kexin Type 1

SEMA6A Semaphorin 6A CTTN Cortactin

SLC1A3

Solute Carrier Family 1 (Glial High Affinity Glutamate Transporter), Member 3

ROBO2 Roundabout, Axon Guidance Receptor, Homolog 2 (Drosophila) SLC38A1 Solute Carrier Family 38, Member 1

LRP2 Low Density Lipoprotein Receptor-Related Protein 2

MERTK MER Proto-Oncogene, Tyrosine Kinase

THEM4 Thioesterase Superfamily Member 4

APBB1 Amyloid Beta (A4) Precursor Protein-Binding, Family B, Member 1 (Fe65) NEFL Neurofilament, Light Polypeptide

LCP1 Lymphocyte Cytosolic Protein 1 (L-Plastin) NMU Neuromedin U

GO:0030424 **Axon**

NCAM1 Neural Cell Adhesion Molecule 1

SEMA6A Semaphorin 6A

PCSK1 Proprotein Convertase Subtilisin/Kexin Type 1

UCHL1 Ubiquitin Carboxyl-Terminal Esterase L1 (Ubiquitin Thiolesterase) ROBO2 Roundabout, Axon Guidance Receptor, Homolog 2 (Drosophila) SLC38A1 Solute Carrier Family 38, Member 1

NEFL Neurofilament, Light Polypeptide

NMU Neuromedin U SDC3 Syndecan 3

GO:0000904 **Cell Morphogenesis Involved In Differentiation**

SLITRK2 SLIT And NTRK-Like Family, Member 2

SEMA5A Semaphorin 5A

SLITRK1 SLIT And NTRK-Like Family, Member 1

SEMA6A Semaphorin 6A

SLC1A3

Solute Carrier Family 1 (Glial High Affinity Glutamate Transporter), Member 3

UCHL1 Ubiquitin Carboxyl-Terminal Esterase L1 (Ubiquitin Thiolesterase) EFNA5 Ephrin-A5

ROBO2 Roundabout, Axon Guidance Receptor, Homolog 2 (Drosophila)

NEFL Neurofilament, Light Polypeptide

APBB1

Amyloid Beta (A4) Precursor Protein-Binding, Family B, Member 1 (Fe65)

FN1 Fibronectin 1

GO:0048667 **Cell Morphogenesis Involved In Neuron Differentiation**

SLITRK2 SLIT And NTRK-Like Family, Member 2

SEMA5A Semaphorin 5A

SLITRK1 SLIT And NTRK-Like Family, Member 1

SEMA6A Semaphorin 6A

SLC1A3 Solute Carrier Family 1 (Glial High Affinity Glutamate Transporter), Member 3

UCHL1 Ubiquitin Carboxyl-Terminal Esterase L1 (Ubiquitin Thiolesterase) EFNA5 Ephrin-A5

ROBO2 Roundabout, Axon Guidance Receptor, Homolog 2 (Drosophila) NEFL Neurofilament, Light Polypeptide

APBB1 Amyloid Beta (A4) Precursor Protein-Binding, Family B, Member 1 (Fe65) GO:0007409 **Axonogenesis**

SLITRK2 SLIT And NTRK-Like Family, Member 2

SEMA5A Semaphorin 5A

SLITRK1

SEMA6A UCHL1

EFNA5

ROB02

NEFL APBB1
