# Supplementary material for: Reprogramming human A375 amelanotic melanoma cells by catalase overexpression: Upregulation of antioxidant genes correlates with regression of melanoma malignancy and with malignant progression when downregulated
Source: Oncotarget. 2016 May 10;7(27):41154–71. doi: 10.18632/oncotarget.9273 (PMC5173049; doi:10.18632/oncotarget.9273)
Supplement: Supplementary file 4 [file oncotarget-07-41154-s004.docx]

**Table S3. Gene Set Enrichment Analysis Report.** Up and Downregulation of coexpressed clustered genes defined *a priori* from KEGG and GO. Table shows clustered genes significantly enriched with a pValue <0.05 and FDR (%) <25.

**Category Term pValue Genesa**

**Upregulated A7 vs Control**

**Genes up or down reg/total**

**FDR (%)**

| KEGG:04514 | Cell Adhesion Molecules (CAMS) | 0.003683241 | **CNTN1**, NEGR1, SELL, F11R /// TSTD1, SDC3  ABCD2, MPV17, PHYH, CAT, PEX5, DHRS4 | 4/46 | 5.7464566 |
| --- | --- | --- | --- | --- | --- |
| KEGG:04146 | Peroxisome | 0.012567325 | /// DHRS4L2, PEX11A, SCP2, ECH1, PEX19, PEX12, ACOX1, ACSL3, FAR2, PEX16, | 21/46 | 11.659003 |
|  |  |  | GNPAT, HMGCL, MPV17L, PEX13, MVK, |  |  |
|  |  |  | PMVK |  |  |
|  |  |  | ACADL, NR1H3, PDPK1 /// FLJ42627, |  |  |
| KEGG:03320 | PPAR Signaling Pathway | 0.029143898 | PDPK1 /// FLJ42627, ILK /// ILK-2, SORBS1, SLC27A1, SCP2, ACADM, PPARA, | 16/46 | 14.386743 |
|  |  |  | DBI, ACOX1, SLC27A5, SCD, CYP27A1, |  |  |
|  |  |  | ACSL3 |  |  |
| KEGG:04210 | Apoptosis | 0.029462738 | IL1R1, PIK3R3, BIRC3, PRKACB, CASP10,  TP53, CASP9, CASP7, CHP, PRKAR1A | 10/46 | 10.892898 |

KEGG:04916 Melanogenesis 0.032490976

GNAO1, ADCY9 6/46 9.646684

GO:0022610 Biological Adhesion 0.0 TESK2, TESC, PTPRK, DLC1, SORBS1 5/11 1.441015

GO:0034112 Positive Regulation Of Homotypic Cell-Cell Adhesion 0.00201207 TESK2, PTPRK, DLC1, SORBS1 4/9 3.5466198

GO:0051894 Positive Regulation Of Focal Adhesion Assembly 0.001980198 SYK, TESK2, PTPRK, DLC1, SORBS1 5/27 5.1416494

**Upregulated G10 vs Control**

KEGG:04012 ERBB Signaling Pathway 0.019417476

JUN, ERBB3, SOS2, PRKCA, ERBB2, STAT5A, CAMK2G, NRG3, PIK3CD, STAT5B, PIK3R3, CDKN1B, MAP2K4, CRK, RPS6KB1, ABL2, AKT3

17/46 14.089137

GO:0030890 Positive Regulation Of B Cell Proliferation 0.0 IL7, CD74, IRS2 3/10 1.4020888

GO:0043542 Endothelial Cell Migration 0.005964215 TGFB2, VCAN 2/3 23.078133

GO:0007157 Heterophilic Cell-Cell Adhesion 0.033797216 NCAM2 1/4 20.335007

GO:0016338 Calcium-Independent Cell-Cell Adhesion 0.012295082 CDH13, NLGN1 2/3 18.508154

GO:0090136 Epithelial Cell-Cell Adhesion 0.008080808 CDH13, NLGN1 2/3 18.547845

GO:0033627 Cell Adhesion Mediated By Integrin 0.011904762 ADAM9, TGFB2 2/3 19.129702

GO:0060355 Positive Regulation Of Cell Adhesion Molecule Production 0.0498155 CYFIP2, PSEN1, ANXA9 /// FAM63A, DLG1,

SRPX2

5/25 17.903647

| GO:0017144 | Drug Metabolic Process | 0.04517454 | MED24, DAXX, MED4, DAXX, DAXX, MED1,  MED13, MED12, KDM3A, NCOA2, ARID1A | 11/17 | 19.391984 |
| --- | --- | --- | --- | --- | --- |
| GO:0045767 | Regulation Of Anti-Apoptosis | 0.038986355 | IRS2, DDX42 | 2/4 | 19.760668 |
| GO:0051893 | Regulation Of Focal Adhesion Assembly | 0.04661017 | NCAM2, PTEN | 2/8 | 19.312409 |
| GO:0032415 | Regulation Of Sodium:Hydrogen Antiporter Activity | 0.04109589 | ETFDH, ACADVL, ACADL | 3/4 | 19.818626 |
| **Upregulated G10 vs A7** | |  |  |  |  |
| GO:0017144 | Drug Metabolic Process | 0.0 | NCOA2, MED24, MED12, DAXX, DAXX,DAXX, MED30, ARID1A, MED13, MED1, MED4 | 11/17 | 1.0963345 |
| GO:0030890 | Positive Regulation Of B Cell Proliferation | 0.0022075055 | CD74, IRS2 | 2/10 | 2.4901716 |
| GO:0001755 | Neural Crest Cell Migration | 0.002 | SLIT2, FGF2 /// NUDT6, ROBO1, SRF | 4/4 | 20.995234 |
| GO:0043535 | Regulation Of Blood Vessel Endothelial Cell Migration | 0.008213553 | SLIT2, FGF2 /// NUDT6, ROBO1, SRF | 4/4 | 20.362832 |
| GO:0002041 | Intussusceptive Angiogenesis | 0.008316008 | SLIT2, FGF2 /// NUDT6, ROBO1, SRF | 4/4 | 16.920808 |
| GO:0016338 | Calcium-Independent Cell-Cell Adhesion | 0.0019723866 | CDH13, NLGN1 | 2/3 | 17.559321 |
| GO:0090136 | Epithelial Cell-Cell Adhesion | 0.0056926 | CDH13, NLGN1 | 2/3 | 17.232442 |
| GO:0071603 | Endothelial Cell-Cell Adhesion | 0.03736264 | CLDN10 /// DZIP1, CLDN1, CLDN4, CLDN20 | 4/20 | 18.409115 |
| GO:0016337 | Cell-Cell Adhesion | 0.02838428 | CLDN10 /// DZIP1, CLDN1, CLDN4, CLDN20 | 4/20 | 17.703538 |
| GO:0001667 | Ameboidal Cell Migration | 0.037894737 | GDNF, HTR2B | 2/4 | 20.88966 |
| GO:0043534 | Blood Vessel Endothelial Cell Migration | 0.040511727 | GDNF, HTR2B | 2/4 | 19.342494 |
| GO:0006930 | Substrate-Bound Cell Migration Cellextension | 0.023554605 | VCAN, TGFB2 | 2/3 | 18.120942 |
| GO:0043542 | Endothelial Cell Migration | 0.021359224 | VCAN, TGFB2 | 2/3 | 17.19144 |
| GO:0033627 | Cell Adhesion Mediated By Integrin | 0.04329897 | TGFB2, ADAM9 | 2/3 | 19.26417 |

**Downregulated A7 vs Control**

Positive Regulation Of Cell Migration Involved In Sprouting

GO:0090050

Angiogenesis 0.0

THBS1, EDN1, IL18, NOX1, HOXB3, SCARB1, ROBO4, IL8, ACVRL1, KDR, VEGFA, ANXA3, AGT, ITGA5, HMOX1, ANGPT1, ID1, WNT5A

18/43 7.913083

GO:0016337 Cell-Cell Adhesion 0.008 CLDN17, CLDN14, CLDN1, CLDN4, CLDN22

/// WWC2, CLDN20 6/20 5.22783

Regulation Of Angiogenesis 0.008752735 SPHK1, VEGFA, F3, ANXA3, IL1B, AQP1 /// INMT, BTG1, RUNX1, IL1A, WNT5A

GO:0045765

Endothelial Cell-Cell Adhesion 0.0040650405 CLDN17, CLDN14, CLDN1, CLDN4, CLDN22

GO:0071603

/// WWC2, CLDN20

Positive Regulation Of Endothelial Cell Proliferation 0.012605042 CDH13, KDR, VEGFA, F3, CCL26, HMGB2, BMP2, WNT5A

GO:0001938

10/24 4.889663

6/20 3.7313946

8/23 9.33401

Negative Regulation Of Cell Migration Involved In Sprouting

GO:0090051

Angiogenesis 0.046413504

FOXC2, THBS1, APOH, BMP10, PDPN,

ACVRL1, NR2F2 7/13 20.533976

GO:0001937 Negative Regulation Of Endothelial Cell Proliferation 0.042283297 SCG2, THBS1, APOH, NR2F2, ENG, CAV2,

CAV1

7/17 17.781743

GO:0010634 Positive Regulation Of Epithelial Cell Migration 0.028282829 SLIT2, SERPINE1, TRIB1, IGFBP3 4/5 18.0417

Positive Regulation Of Epithelial Cell Proliferation Involved In

GO:0060054

Wound Healing 0.032327585

SCG2, THBS1, APOH, NR2F2, ENG, CAV2,

CAV1 7/17 17.162377

GO:0034111 Negative Regulation Of Homotypic Cell-Cell Adhesion 0.036893204 TPM1 /// LOC100128, SAA1, ERBB2, TPM1,

TGM2

5/12 19.00197

**Downregulated G10 vs Control**

KEGG:04110 Cell Cycle 0.0

KEGG:04810 Regulation Of Actin Cytoskeleton 0.02886598

**Downregulated G10 vs A7**

KEGG:04110 Cell Cycle 0.0

KEGG:03320 PPAR Signaling Pathway 0.017699115

MAD2L2, WEE1, CDC14A, HDAC1, TGFB3, SMAD3, WEE1, GADD45A, WEE1, CDC6, CCND1, ORC6L,ESPL1, CHEK1,CCNB2,PKMYT1,ORC1L,CDC2, BUB1B /// PAK6, ATM /// NPAT,PLK1

FGF19, CHRM4, RRAS2, PIP5K1A, ACTN2, WASF2, NRAS /// CSDE1, CDC42, HRAS, PPP1CA, PAK1, ITGA8, ITGA5, PDGFD, RDX, ITGA10

PKMYT1, HDAC1, GADD45A, CDK2, CDC14A, CDC20, ORC6L, ORC1L, CCND1, CDC6, WEE1, CHEK1, WEE1, WEE1, BUB1B

/// PAK6, PLK1, ATM /// NPAT

PDPK1 /// FLJ42627, PDPK1, PDPK1, SLC27A1, PDPK1 /// FLJ42627, FABP5 /// FABP5L3, PPARA, APOC3, SCD, FABP3, CYP4A22, ACADM, ACSL3, DBI, MMP1, CPT1A, ACADL, NR1H3, ILK /// ILK-2, FADS2, RXRG

21/46 0.35333333

16/46 23.597974

18/46 0.469697

21/46 12.186814

KEGG:04210 Apoptosis 0.018450184 CASP8, BIRC2, APAF1, BIRC3, CASP10, PRKACB, ATM /// NPAT, ENDOD1, IL1R1

9/46 15.750161

a Complete gene titles within each process are detailed in Table 4S
