# Supplementary material for: Reprogramming human A375 amelanotic melanoma cells by catalase overexpression: Upregulation of antioxidant genes correlates with regression of melanoma malignancy and with malignant progression when downregulated
Source: Oncotarget. 2016 May 10;7(27):41154–71. doi: 10.18632/oncotarget.9273 (PMC5173049; doi:10.18632/oncotarget.9273)
Supplement: Supplementary file 5 [file oncotarget-07-41154-s005.docx]

**Table S4.** Significant processes obtained after GSEA analysis with their corresponding gene symbols and gene titles.

**Gene Symbol Gene Title**

**Upregulated A7 vs Control**

KEGG:04514 **Cell Adhesion Molecules (CAMS)**

CNTN1 contactin 1

NEGR1 neuronal growth regulator 1

SELL selectin L

F11 receptor /// thiosulfate sulfurtransferase (rhodanese)-like

F11R /// TSTD1

KEGG:04146 **Peroxisome**

domain containing 1

ABCD2 ATP-binding cassette, sub-family D (ALD), member 2

MPV17 MpV17 mitochondrial inner membrane protein

PHYH phytanoyl-CoA 2-hydroxylase

CAT catalase

PEX5 peroxisomal biogenesis factor 5

DHRS4 /// DHRS4L2 mber 4 like 1

PEX11A peroxisomal biogenesis factor 11 alpha

SCP2 sterol carrier protein 2

enoyl CoA hydratase 1, peroxisomal /// enoyl Coenzyme A

ECH1

hydratase 1, peroxisomal

PEX19 peroxisomal biogenesis factor 19

PEX12 peroxisomal biogenesis factor 12

acyl-CoA oxidase 1, palmitoyl /// acyl-Coenzyme A oxidase 1,

ACOX1

palmitoyl

ACSL3 acyl-CoA synthetase long-chain family member 3

FAR2 fatty acyl CoA reductase 2

PEX16 peroxisomal biogenesis factor 16

GNPAT glyceronephosphate O-acyltransferase

3-hydroxymethyl-3-methylglutaryl-CoA lyase /// 3-hydroxymethyl-3-

HMGCL

methylglutaryl-Coenzyme A lyase

MPV17L MPV17 mitochondrial membrane protein-like

PEX13 peroxisomal biogenesis factor 13

MVK mevalonate kinase

PMVK phosphomevalonate kinase

KEGG:03320 **PPAR Signaling Pathway**

ACADL

acyl-CoA dehydrogenase, long chain /// acyl-Coenzyme A

dehydrogenase, long chain

NR1H3 nuclear receptor subfamily 1, group H, member 3

3-phosphoinositide dependent protein kinase-1 /// hypothetical

PDPK1 /// FLJ42627

PDPK1 /// FLJ42627

LOC645644

3-phosphoinositide dependent protein kinase-1 /// hypothetical

LOC645644

ILK /// ILK-2 integrin-linked kinase /// integrin-linked kinase-2

SORBS1 sorbin and SH3 domain containing 1

SLC27A1 solute carrier family 27 (fatty acid transporter), member 1

SCP2 sterol carrier protein 2

acyl-CoA dehydrogenase, C-4 to C-12 straight chain /// acyl-

ACADM

Coenzyme A dehydrogenase, C-4 to C-12 straight chain

PPARA peroxisome proliferator-activated receptor alpha

DBI e A binding protein)

ACOX1

acyl-CoA oxidase 1, palmitoyl /// acyl-Coenzyme A oxidase 1, palmitoyl

SLC27A5 solute carrier family 27 (fatty acid transporter), member 5

SCD stearoyl-CoA desaturase (delta-9-desaturase)

CYP27A1 cytochrome P450, family 27, subfamily A, polypeptide 1

ACSL3 acyl-CoA synthetase long-chain family member 3

KEGG:04210 **Apoptosis**

IL1R1 interleukin 1 receptor, type I

PIK3R3 phosphoinositide-3-kinase, regulatory subunit 3 (gamma) BIRC3 baculoviral IAP repeat-containing 3

PRKACB protein kinase, cAMP-dependent, catalytic, beta CASP10 caspase 10, apoptosis-related cysteine peptidase TP53 tumor protein p53

CASP9 caspase 9, apoptosis-related cysteine peptidase CASP7 caspase 7, apoptosis-related cysteine peptidase CHP calcium binding protein P22

protein kinase, cAMP-dependent, regulatory, type I, alpha (tissue

PRKAR1A

KEGG:04916 **Melanogenesis**

specific extinguisher 1)

TYR tyrosinase (oculocutaneous albinism IA)

tyrosinase (oculocutaneous albinism IA) /// tyrosinase-like

TYR /// TYRL

(pseudogene)

PRKACB protein kinase, cAMP-dependent, catalytic, beta

PRKCA protein kinase C, alpha

guanine nucleotide binding protein (G protein), alpha activating

GNAO1

activity polypeptide O

ADCY9 adenylate cyclase 9

GO:0022610 **Biological Adhesion**

TESK2 testis-specific kinase 2

TESC tescalcin

PTPRK protein tyrosine phosphatase, receptor type, K DLC1 deleted in liver cancer 1

SORBS1 sorbin and SH3 domain containing 1

GO:0034112 **Positive Regulation Of Homotypic Cell-Cell Adhesion**

TESK2 testis-specific kinase 2

PTPRK protein tyrosine phosphatase, receptor type, K DLC1 deleted in liver cancer 1

SORBS1 sorbin and SH3 domain containing 1

GO:0051894 **Positive Regulation Of Focal Adhesion Assembly** SYK spleen tyrosine kinase TESK2 testis-specific kinase 2

PTPRK protein tyrosine phosphatase, receptor type, K DLC1 deleted in liver cancer 1

SORBS1 sorbin and SH3 domain containing 1

**Upregulated G10 vs Control**

KEGG:04012 **ERBB Signaling Pathway**

JUN jun oncogene

ERBB3 v-erb-b2 erythroblastic leukemia viral oncogene homolog 3 (avian) SOS2 son of sevenless homolog 2 (Drosophila)

PRKCA protein kinase C, alpha

ERBB2

v-erb-b2 erythroblastic leukemia viral oncogene homolog 2, neuro/glioblastoma derived oncogene homolog (avian)

STAT5A signal transducer and activator of transcription 5A CAMK2G calcium/calmodulin-dependent protein kinase II gamma NRG3 neuregulin 3

PIK3CD phosphoinositide-3-kinase, catalytic, delta polypeptide

STAT5B signal transducer and activator of transcription 5B

PIK3R3 phosphoinositide-3-kinase, regulatory subunit 3 (gamma) CDKN1B cyclin-dependent kinase inhibitor 1B (p27, Kip1)

MAP2K4 mitogen-activated protein kinase kinase 4

CRK v-crk sarcoma virus CT10 oncogene homolog (avian) RPS6KB1 ribosomal protein S6 kinase, 70kDa, polypeptide 1

v-abl Abelson murine leukemia viral oncogene homolog 2 (arg,

ABL2

AKT3

Abelson-related gene)

v-akt murine thymoma viral oncogene homolog 3 (protein kinase B, gamma)

GO:0030890 **Positive Regulation Of B Cell Proliferation**

IL7 interleukin 7

CD74 molecule, major histocompatibility complex, class II invariant

CD74

chain

IRS2 insulin receptor substrate 2

GO:0043542 **Endothelial Cell Migration**

TGFB2 transforming growth factor, beta 2

VCAN versican

GO:0007157 **Heterophilic Cell-Cell Adhesion**

NCAM2 neural cell adhesion molecule 2

GO:0016338 **Calcium-Independent Cell-Cell Adhesion**

CDH13 cadherin 13, H-cadherin (heart) NLGN1 neuroligin 1

GO:0090136 **Epithelial Cell-Cell Adhesion**

CDH13 cadherin 13, H-cadherin (heart) NLGN1 neuroligin 1

GO:0033627 **Cell Adhesion Mediated By Integrin**

ADAM9 ADAM metallopeptidase domain 9 (meltrin gamma) TGFB2 transforming growth factor, beta 2

GO:0060355 **Positive Regulation Of Cell Adhesion Molecule Production**

CYFIP2 cytoplasmic FMR1 interacting protein 2

PSEN1 presenilin 1

ANXA9 /// FAM63A annexin A9 /// family with sequence similarity 63, member A DLG1 discs, large homolog 1 (Drosophila)

SRPX2 sushi-repeat-containing protein, X-linked 2

GO:0017144 **Drug Metabolic Process**

MED24 mediator complex subunit 24

DAXX death-domain associated protein

MED4 mediator complex subunit 4

DAXX death-domain associated protein DAXX death-domain associated protein MED1 mediator complex subunit 1

MED13 mediator complex subunit 13

MED12 mediator complex subunit 12

KDM3A lysine (K)-specific demethylase 3A

NCOA2 nuclear receptor coactivator 2

ARID1A AT rich interactive domain 1A (SWI-like)

GO:0045767 **Regulation Of Anti-Apoptosis**

IRS2 insulin receptor substrate 2

DDX42 DEAD (Asp-Glu-Ala-Asp) box polypeptide 42

GO:0051893 **Regulation Of Focal Adhesion Assembly**

NCAM2 neural cell adhesion molecule 2

PTEN phosphatase and tensin homolog

GO:0032415 **Regulation Of Sodium:Hydrogen Antiporter Activity**

ETFDH electron-transferring-flavoprotein dehydrogenase

acyl-CoA dehydrogenase, very long chain /// acyl-Coenzyme A

ACADVL

ACADL

**Upregulated G10 vs A7**

GO:0017144 **Drug Metabolic Process**

dehydrogenase, very long chain

acyl-CoA dehydrogenase, long chain /// acyl-Coenzyme A

dehydrogenase, long chain

NCOA2 nuclear receptor coactivator 2

MED24 mediator complex subunit 24

MED12 mediator complex subunit 12

DAXX death-domain associated protein DAXX death-domain associated protein DAXX death-domain associated protein MED30 mediator complex subunit 30

ARID1A AT rich interactive domain 1A (SWI-like) MED13 mediator complex subunit 13

MED1 mediator complex subunit 1

MED4 mediator complex subunit 4

GO:0030890 **Positive Regulation Of B Cell Proliferation**

CD74 CD74 molecule, major histocompatibility complex, class II invariant

chain

IRS2 insulin receptor substrate 2

GO:0001755 **Neural Crest Cell Migration**

SLIT2 slit homolog 2 (Drosophila)

fibroblast growth factor 2 (basic) /// nudix (nucleoside diphosphate

FGF2 /// NUDT6

linked moiety X)-type motif 6

ROBO1 roundabout, axon guidance receptor, homolog 1 (Drosophila)

serum response factor (c-fos serum response element-binding

SRF

transcription factor)

GO:0043535 **Regulation Of Blood Vessel Endothelial Cell Migration**

SLIT2 slit homolog 2 (Drosophila)

fibroblast growth factor 2 (basic) /// nudix (nucleoside diphosphate

FGF2 /// NUDT6

linked moiety X)-type motif 6

ROBO1 roundabout, axon guidance receptor, homolog 1 (Drosophila)

serum response factor (c-fos serum response element-binding

SRF

transcription factor)

GO:0002041 **Intussusceptive Angiogenesis**

SLIT2 slit homolog 2 (Drosophila)

fibroblast growth factor 2 (basic) /// nudix (nucleoside diphosphate

FGF2 /// NUDT6

linked moiety X)-type motif 6

ROBO1 roundabout, axon guidance receptor, homolog 1 (Drosophila)

serum response factor (c-fos serum response element-binding

SRF

transcription factor)

GO:0016338 **Calcium-Independent Cell-Cell Adhesion**

CDH13 cadherin 13, H-cadherin (heart) NLGN1 neuroligin 1

GO:0090136 **Epithelial Cell-Cell Adhesion**

CDH13 cadherin 13, H-cadherin (heart) NLGN1 neuroligin 1

GO:0071603 **Endothelial Cell-Cell Adhesion**

CLDN10 /// DZIP1 claudin 10 /// DAZ interacting protein 1

CLDN1 claudin 1

CLDN4 claudin 4

CLDN20 claudin 20

GO:0016337 **Cell-Cell Adhesion**

CLDN10 /// DZIP1 claudin 10 /// DAZ interacting protein 1

CLDN1 claudin 1

CLDN4 claudin 4

CLDN20 claudin 20

GO:0001667 **Ameboidal Cell Migration**

GDNF glial cell derived neurotrophic factor

HTR2B 5-hydroxytryptamine (serotonin) receptor 2B

GO:0043534 **Blood Vessel Endothelial Cell Migration**

GDNF glial cell derived neurotrophic factor

HTR2B 5-hydroxytryptamine (serotonin) receptor 2B

GO:0006930 **Substrate-Bound Cell Migration Cellextension**

VCAN versican

TGFB2 transforming growth factor, beta 2

GO:0043542 **Endothelial Cell Migration**

VCAN versican

TGFB2 transforming growth factor, beta 2

GO:0033627 **Cell Adhesion Mediated By Integrin**

TGFB2 transforming growth factor, beta 2

ADAM9 ADAM metallopeptidase domain 9 (meltrin gamma)

**Downregulated A7 vs Control**

GO:0090050 **Positive Regulation Of Cell Migration Involved In Sprouting Angiogenesis**

THBS1 thrombospondin 1

EDN1 endothelin 1

IL18 interleukin 18 (interferon-gamma-inducing factor) NOX1 NADPH oxidase 1

HOXB3 homeobox B3

SCARB1 scavenger receptor class B, member 1

ROBO4 roundabout homolog 4, magic roundabout (Drosophila) IL8 interleukin 8

ACVRL1 activin A receptor type II-like 1

KDR kinase insert domain receptor (a type III receptor tyrosine kinase) VEGFA vascular endothelial growth factor A

ANXA3 annexin A3

AGT angiotensinogen (serpin peptidase inhibitor, clade A, member 8) ITGA5 integrin, alpha 5 (fibronectin receptor, alpha polypeptide) HMOX1 heme oxygenase (decycling) 1

ANGPT1 angiopoietin 1

ID1 inhibitor of DNA binding 1, dominant negative helix-loop-helix protein

WNT5A wingless-type MMTV integration site family, member 5A

GO:0016337 **Cell-Cell Adhesion**

CLDN17 claudin 17

CLDN14 claudin 14

CLDN1 claudin 1

CLDN4 claudin 4

CLDN22 /// WWC2 claudin 22 /// WW and C2 domain containing 2

CLDN20 claudin 20

GO:0045765 **Regulation Of Angiogenesis**

SPHK1 sphingosine kinase 1

VEGFA vascular endothelial growth factor A

F3 coagulation factor III (thromboplastin, tissue factor) ANXA3 annexin A3

IL1B interleukin 1, beta

aquaporin 1 (Colton blood group) /// indolethylamine N-

AQP1 /// INMT

methyltransferase

BTG1 B-cell translocation gene 1, anti-proliferative

RUNX1 runt-related transcription factor 1

IL1A interleukin 1, alpha

WNT5A wingless-type MMTV integration site family, member 5A

GO:0071603 **Endothelial Cell-Cell Adhesion**

CLDN17 claudin 17

CLDN14 claudin 14

CLDN1 claudin 1

CLDN4 claudin 4

CLDN22 /// WWC2 claudin 22 /// WW and C2 domain containing 2

CLDN20 claudin 20

GO:0001938 **Positive Regulation Of Endothelial Cell Proliferation**

CDH13 cadherin 13, H-cadherin (heart)

KDR kinase insert domain receptor (a type III receptor tyrosine kinase) VEGFA vascular endothelial growth factor A

F3 coagulation factor III (thromboplastin, tissue factor) CCL26 chemokine (C-C motif) ligand 26

HMGB2 high-mobility group box 2

BMP2 bone morphogenetic protein 2

WNT5A wingless-type MMTV integration site family, member 5A

GO:0090051 **Negative Regulation Of Cell Migration Involved In Sprouting Angiogenesis** FOXC2 forkhead box C2 (MFH-1, mesenchyme forkhead 1) THBS1 thrombospondin 1

APOH apolipoprotein H (beta-2-glycoprotein I) BMP10 bone morphogenetic protein 10

PDPN podoplanin

ACVRL1 activin A receptor type II-like 1

NR2F2 nuclear receptor subfamily 2, group F, member 2

GO:0001937 **Negative Regulation Of Endothelial Cell Proliferation**

SCG2 secretogranin II /// secretogranin II (chromogranin C) THBS1 thrombospondin 1

APOH apolipoprotein H (beta-2-glycoprotein I)

NR2F2 nuclear receptor subfamily 2, group F, member 2

ENG endoglin

CAV2 caveolin 2

CAV1 caveolin 1, caveolae protein, 22kDa

GO:0010634 **Positive Regulation Of Epithelial Cell Migration**

SLIT2 slit homolog 2 (Drosophila)

serpin peptidase inhibitor, clade E (nexin, plasminogen activator

SERPINE1

inhibitor type 1), member 1

TRIB1 tribbles homolog 1 (Drosophila)

IGFBP3 insulin-like growth factor binding protein 3

GO:0060054 **Positive Regulation Of Epithelial Cell Proliferation Involved In Wound Healing** SCG2 secretogranin II /// secretogranin II (chromogranin C) THBS1 thrombospondin 1

APOH apolipoprotein H (beta-2-glycoprotein I)

NR2F2 nuclear receptor subfamily 2, group F, member 2

ENG endoglin

CAV2 caveolin 2

CAV1 caveolin 1, caveolae protein, 22kDa

GO:0034111 **Negative Regulation Of Homotypic Cell-Cell Adhesion**

TPM1/// LOC100128 tropomyosin 1 (alpha) /// hypothetical protein LOC100128979

SAA1 serum amyloid A1

v-erb-b2 erythroblastic leukemia viral oncogene homolog 2,

ERBB2

neuro/glioblastoma derived oncogene homolog (avian)

TPM1 tropomyosin 1 (alpha)

transglutaminase 2 (C polypeptide, protein-glutamine-gamma-

TGM2

**Downregulated G10 vs Control**

KEGG:04110 **Cell Cycle**

glutamyltransferase)

MAD2L2 MAD2 mitotic arrest deficient-like 2 (yeast) WEE1 WEE1 homolog (S. pombe)

CDC14A CDC14 cell division cycle 14 homolog A (S. cerevisiae) HDAC1 histone deacetylase 1

TGFB3 transforming growth factor, beta 3

SMAD3 SMAD family member 3

WEE1 WEE1 homolog (S. pombe)

GADD45A growth arrest and DNA-damage-inducible, alpha

WEE1 WEE1 homolog (S. pombe)

CDC6 cell division cycle 6 homolog (S. cerevisiae) CCND1 cyclin D1

ORC6L origin recognition complex, subunit 6 like (yeast) ESPL1 extra spindle pole bodies homolog 1 (S. cerevisiae) CHEK1 CHK1 checkpoint homolog (S. pombe)

CCNB2 cyclin B2

PKMYT1 protein kinase, membrane associated tyrosine/threonine 1

ORC1L origin recognition complex, subunit 1-like (yeast) CDC20 cell division cycle 20 homolog (S. cerevisiae)

budding uninhibited by benzimidazoles 1 homolog beta (yeast) ///

BUB1B /// PAK6

ATM /// NPAT

p21 protein (Cdc42/Rac)-activated kinase 6

ataxia telangiectasia mutated /// nuclear protein, ataxia- telangiectasia locus

PLK1 polo-like kinase 1 (Drosophila) KEGG:04810 **Regulation Of Actin Cytoskeleton**

FGF19 fibroblast growth factor 19

CHRM4 cholinergic receptor, muscarinic 4

RRAS2 related RAS viral (r-ras) oncogene homolog 2

PIP5K1A phosphatidylinositol-4-phosphate 5-kinase, type I, alpha

ACTN2 actinin, alpha 2

WASF2 WAS protein family, member 2

neuroblastoma RAS viral (v-ras) oncogene homolog /// cold shock

NRAS /// CSDE1

domain containing E1, RNA-binding

CDC42 cell division cycle 42 (GTP binding protein, 25kDa) HRAS v-Ha-ras Harvey rat sarcoma viral oncogene homolog

protein phosphatase 1, catalytic subunit, alpha isozyme /// protein

PPP1CA

phosphatase 1, catalytic subunit, alpha isoform

PAK1 p21 protein (Cdc42/Rac)-activated kinase 1

ITGA8 integrin, alpha 8

ITGA5 integrin, alpha 5 (fibronectin receptor, alpha polypeptide) PDGFD platelet derived growth factor D

RDX radixin

ITGA10 integrin, alpha 10

**Downregulated G10 vs A7**

KEGG:04110 **Cell Cycle**

PKMYT1 protein kinase, membrane associated tyrosine/threonine 1

HDAC1 histone deacetylase 1

GADD45A growth arrest and DNA-damage-inducible, alpha

CDK2 cyclin-dependent kinase 2

CDC14A CDC14 cell division cycle 14 homolog A (S. cerevisiae) CDC20 cell division cycle 20 homolog (S. cerevisiae)

ORC6L origin recognition complex, subunit 6 like (yeast) ORC1L origin recognition complex, subunit 1-like (yeast) CCND1 cyclin D1

CDC6 cell division cycle 6 homolog (S. cerevisiae) WEE1 WEE1 homolog (S. pombe)

CHEK1 CHK1 checkpoint homolog (S. pombe) WEE1 WEE1 homolog (S. pombe)

WEE1 WEE1 homolog (S. pombe)

budding uninhibited by benzimidazoles 1 homolog beta (yeast) ///

BUB1B /// PAK6

p21 protein (Cdc42/Rac)-activated kinase 6

PLK1 polo-like kinase 1 (Drosophila)

ataxia telangiectasia mutated /// nuclear protein, ataxia-

ATM /// NPAT

telangiectasia locus

KEGG:03320 **PPAR Signaling Pathway**

PDPK1 /// FLJ42627

3-phosphoinositide dependent protein kinase-1 /// hypothetical

LOC645644

PDPK1 3-phosphoinositide dependent protein kinase-1

PDPK1 3-phosphoinositide dependent protein kinase-1

SLC27A1 solute carrier family 27 (fatty acid transporter), member 1

3-phosphoinositide dependent protein kinase-1 /// hypothetical

PDPK1 /// FLJ42627

FABP5 /// FABP5L3

LOC645644

fatty acid binding protein 5 (psoriasis-associated) /// fatty acid binding protein 5-like 3 (pseudogene)

PPARA peroxisome proliferator-activated receptor alpha

APOC3 apolipoprotein C-III

SCD stearoyl-CoA desaturase (delta-9-desaturase)

fatty acid binding protein 3, muscle and heart (mammary-derived

FABP3

growth inhibitor)

CYP4A22 cytochrome P450, family 4, subfamily A, polypeptide 22

acyl-CoA dehydrogenase, C-4 to C-12 straight chain /// acyl-

ACADM

Coenzyme A dehydrogenase, C-4 to C-12 straight chain

ACSL3 acyl-CoA synthetase long-chain family member 3

DBI e A binding protein)

MMP1 matrix metallopeptidase 1 (interstitial collagenase) CPT1A carnitine palmitoyltransferase 1A (liver)

acyl-CoA dehydrogenase, long chain /// acyl-Coenzyme A

ACADL

dehydrogenase, long chain

NR1H3 nuclear receptor subfamily 1, group H, member 3

ILK /// ILK-2 integrin-linked kinase /// integrin-linked kinase-2

FADS2 fatty acid desaturase 2

RXRG retinoid X receptor, gamma

KEGG:04210 **Apoptosis**

CASP8 caspase 8, apoptosis-related cysteine peptidase

BIRC2 baculoviral IAP repeat-containing 2

APAF1 apoptotic peptidase activating factor 1

BIRC3 baculoviral IAP repeat-containing 3

CASP10 caspase 10, apoptosis-related cysteine peptidase

PRKACB protein kinase, cAMP-dependent, catalytic, beta

ataxia telangiectasia mutated /// nuclear protein, ataxia-

ATM /// NPAT

telangiectasia locus

ENDOD1 endonuclease domain containing 1

IL1R1 interleukin 1 receptor, type I
