# Supplementary material for: Reprogramming human A375 amelanotic melanoma cells by catalase overexpression: Upregulation of antioxidant genes correlates with regression of melanoma malignancy and with malignant progression when downregulated
Source: Oncotarget. 2016 May 10;7(27):41154–71. doi: 10.18632/oncotarget.9273 (PMC5173049; doi:10.18632/oncotarget.9273)
Supplement: Supplementary file 6 [file oncotarget-07-41154-s006.docx]

**Table S5. List Of Selected Biological Processes To Analyze By GSEA.** 131 gene groups collected from GO

and 19 gene groups from KEGG based on processes and pathways of interest were used.

**Biologic Processess or Signaling Pathways Reference**

Cell Cycle KEGG:04110

Wnt Signaling Pathway KEGG:04310

Regulation Of Actin Cytoskeleton KEGG:04810

TGF‐Beta Signaling Pathway KEGG:04350

P53 Signaling Pathway KEGG:04115

MAPK Signaling Pathway KEGG:04010

VEGF Signaling Pathway KEGG:04370

Chemokine Signaling Pathway KEGG:04062

MTOR Signaling Pathway KEGG:04150

JAK‐STAT Signaling Pathway KEGG:04630

Focal Adhesion KEGG:04510

Melanoma KEGG:05218

Regulation Of Autophagy KEGG:04140

ERBB Signaling Pathway KEGG:04012

PPAR Signaling Pathway KEGG:03320

Melanogenesis KEGG:04916

Apoptosis KEGG:04210

Peroxisome KEGG:04146

Cell Adhesion Molecules (CAMS) KEGG:04514

Positive Regulation Of Cell Migration Involved In Sprouting Angiogenesis GO:0090050

Cell‐Cell Adhesion GO:0016337

Regulation Of Angiogenesis GO:0045765

Endothelial Cell‐Cell Adhesion GO:0071603

Positive Regulation Of Endothelial Cell Proliferation GO:0001938

Negative Regulation Of Cell Migration Involved In Sprouting Angiogenesis GO:0090051

Negative Regulation Of Endothelial Cell Proliferation GO:0001937

Positive Regulation Of Epithelial Cell Migration GO:0010634

Positive Regulation Of Epithelial Cell Proliferation Involved In Wound Healing GO:0060054

Negative Regulation Of Homotypic Cell‐Cell Adhesion GO:0034111

Positive Regulation Of Heterotypic Cell‐Cell Adhesion GO:0034116

Drug Metabolic Process GO:0017144

Calcium‐Independent Cell‐Cell Adhesion GO:0016338

Epithelial Cell‐Cell Adhesion GO:0090136

Angiogenesis GO:0001525

Regulation Of Satellite Cell Proliferation GO:0014842

Wound Healing Spreading Of Epidermal Cells GO:0035313

Negative Regulation Of B Cell Proliferation GO:0030889

Sprouting Angiogenesis GO:0002040

Cellular Response To Oxidative Stress GO:0034599

DNA Damage Response Signal Transduction Resulting In Induction Of Apoptosis GO:0008630

Cell‐Cell Adhesion Involved In Neuronal‐Glial Interactions Involved In Cerebral Cortex

Radial Glia Guided Migration

GO:0021813

Regulation Of Hydrogen Peroxide Metabolic Process GO:0010310

Endothelial Cell Proliferation GO:0001935

Cellular Component Disassembly Involved In Apoptosis GO:0006921

Negative Regulation Of Endothelial Cell Migration GO:0010596

Negative Regulation Of Cell‐Cell Adhesion GO:0022408

Induction Of Apoptosis By Intracellular Signals GO:0008629

Neutrophil Apoptosis GO:0001781

Induction Of Apoptosis In Response To Chemical Stimulus GO:0031558

Negative Regulation Of Translation In Response To Oxidative Stress GO:0032938

Lymphangiogenesis GO:0001946

Positive Regulation Of Sodium:Hydrogen Antiporter Activity GO:0032417

Homotypic Cell‐Cell Adhesion GO:0034109

Substrate‐Bound Cell Migration Cellextension GO:0006930

Endothelial Cell Migration GO:0043542

Positive Regulation Of Angiogenesis GO:0045766

Negative Regulation Of Anti‐Apoptosis GO:0019987

Positive Regulation Of Apoptosis GO:0043065

Regulation Of B Cell Apoptosis GO:0002902

Age‐Dependent Response To Oxidative Stress GO:0001306

Regulation Of Transcription From Rna Polymerase Ii Promoter In Response To

Oxidative Stress GO:0043619

Regulation Of Blood Vessel Endothelial Cell Migration GO:0043535

Regulation Of Mast Cell Apoptosis GO:0033025

Cellular Response To Hydrogen Peroxide GO:0070301

Neural Crest Cell Migration GO:0001755

Intussusceptive Angiogenesis GO:0002041

Negative Regulation Of Sodium:Hydrogen Antiporter Activity GO:0032416

Positive Regulation Of Endothelial Cell Migration GO:0010595

Regulation Of Focal Adhesion Assembly GO:0051893

Negative Regulation Of Heterotypic Cell‐Cell Adhesion GO:0034115

Induction Of Apoptosis By Extracellular Signals GO:0008624

Negative Regulation Of Myeloid Cell Apoptosis GO:0033033

Cell Migration Involved In Sprouting Angiogenesis GO:0002042

Hydrogen Peroxide Biosynthetic Process GO:0050665

Positive Regulation Of Cell Projection Organization GO:0031346

Regulation Of Cell Adhesion GO:0030155

Dna Damage Response Signal Transduction By P53 Class Mediator Resulting In

Induction Of Apoptosis GO:0042771

Induction Of Apoptosis By Ionic Changes GO:0008627

Apoptosis GO:0006915

Apoptosis In Response To Endoplasmic Reticulum Stress GO:0070059

Negative Regulation Of Epithelial Cell Migration GO:0010633

Hydrogen Peroxide Metabolic Process GO:0042743

Regulation Of Anti‐Apoptosis GO:0045767

Blood Vessel Endothelial Cell Migration GO:0043534

Ameboidal Cell Migration GO:0001667

Negative Regulation Of Cell Proliferation GO:0008285

Positive Regulation Of Mast Cell Proliferation GO:0070668

Mitochondrial Fragmentation Involved In Apoptosis GO:0043653

B Cell Apoptosis GO:0001783

Negative Regulation Of Calcium‐Dependent Cell‐Cell Adhesion GO:0046588

Negative Regulation Of Cell Adhesion Involved In Substrate‐Bound Cell Migration GO:0006933

Negative Regulation Of Mast Cell Apoptosis GO:0033026

Negative Regulation Of Mature B Cell Apoptosis GO:0002906

Induction Of Apoptosis Via Death Domain Receptors GO:0008625

Cell Proliferation GO:0008283

Apoptosis Involved In Morphogenesis GO:0060561

Cell Motility GO:0048870

Cellular Response To Reactive Oxygen Species GO:0034614

Anti‐Apoptosis GO:0006916

Convergent Extension Involved In Organogenesis GO:0060029

Negative Regulation Of Cell Migration GO:0030336

Drug Catabolic Process GO:0042737

Drug Export GO:0046618

Positive Regulation Of Neutrophil Apoptosis GO:0033031

Cell Migration GO:0016477

Negative Regulation Of T Cell Apoptosis GO:0070233

Positive Regulation Of Anti‐Apoptosis GO:0045768

Positive Regulation Of Myeloid Cell Apoptosis GO:0033034

Negative Regulation Of T Cell Proliferation GO:0042130

Positive Regulation Of T Cell Proliferation GO:0042102

Hydrogen Peroxide Catabolic Process GO:0042744

Cell Communication GO:0007154

Positive Regulation Of Cell Proliferation GO:0008284

Negative Regulation Of Cell Projection Organization GO:0031345

Homophilic Cell Adhesion GO:0007156

Positive Regulation Of Calcium‐Independent Cell‐Cell Adhesion GO:0051041

Focal Adhesion Assembly GO:0048041

Cell Adhesion Mediated By Integrin GO:0033627

Response To Drug GO:0042493

Response To Reactive Oxygen Species GO:0000302

Blood Vessel Endothelial Cell Proliferation Involved In Sprouting Angiogenesis GO:0002043

Regulation Of Cell Adhesion Mediated By Integrin GO:0033628

Regulation Of Myeloid Cell Apoptosis GO:0033032

Positive Regulation Of Cell Adhesion GO:0045785

Negative Regulation Of Hydrogen Peroxide Metabolic Process GO:0010727

Heterophilic Cell‐Cell Adhesion GO:0007157

Positive Regulation Of B Cell Proliferation GO:0030890

Regulation Of Endothelial Cell Migration GO:0010594

Cell Adhesion GO:0007155

Inflammatory Cell Apoptosis GO:0006925

Positive Regulation Of Calcium‐Dependent Cell‐Cell Adhesion GO:0046587

Fatty Acid Beta‐Oxidation Using Acyl‐Coa Dehydrogenase GO:0033539

Negative Regulation Of Angiogenesis GO:0016525

Negative Regulation Of Focal Adhesion Assembly GO:0051895

Regulation Of Cell‐Cell Adhesion GO:0022407

Substrate‐Bound Cell Migration GO:0006929

Leukocyte Cell‐Cell Adhesion GO:0007159

Substrate‐Bound Cell Migration Cell Attachment To Substrate GO:0006931

Positive Regulation Of Cell‐Cell Adhesion GO:0022409

Neuron Cell‐Cell Adhesion GO:0007158

Regulation Of Apoptosis GO:0042981

Regulation Of Cell Migration GO:0030334

Negative Regulation Of Cell Adhesion

Positive Regulation Of Cell Adhesion Molecule Production

GO:0007162

GO:0060355

Negative Regulation Of Cell Adhesion Mediated By lntegrin GO:0033629

Regulai!on Of Sodium:Hydrogen Antiporter Activity GO:0032415

Molecular Hydrogen Transport

Positive Regulation Of Focal Adhesion Assembly Positive Regulation Of Homotypic Ceii-Cell

Adhesion Biological Adhesion

GO:0015993

GO:0051894

GO:0034112

GO:0022610
