# Supplementary material for: Reprogramming human A375 amelanotic melanoma cells by catalase overexpression: Upregulation of antioxidant genes correlates with regression of melanoma malignancy and with malignant progression when downregulated
Source: Oncotarget. 2016 May 10;7(27):41154–71. doi: 10.18632/oncotarget.9273 (PMC5173049; doi:10.18632/oncotarget.9273)
Supplement: Supplementary file 7 [file oncotarget-07-41154-s007.docx]

**Table S6.** List of 111 selected genes of the antioxidant system with their corresponding gene titles to analyze by GSEA.

**Gene Symbol Gene Title**

NOX4 NADPH Oxidase 4

EPHX2 Epoxide Hydrolase 2, Cytoplasmic

MGST1 Microsomal Glutathione S-Transferase 1

MSRA Methionine Sulfoxide Reductase A MGST2 Microsomal Glutathione S-Transferase 2

GSTM3 Glutathione S-Transferase Mu 3 (Brain) MPV17 Mpv17 Mitochondrial Inner Membrane Protein GSTZ1 Glutathione Transferase Zeta 1

GSTA4 Glutathione S-Transferase Alpha 4

CAT Catalase

GSTO1 Glutathione S-Transferase Omega 1

TXNRD3 /// TXNRD3I Thioredoxin Reductase 3 /// Thioredoxin Reductase 3 Intronic Transcript 1

GSR Glutathione Reductase

GSTM4 /// GSTM2 Glutathione S-Transferase Mu 4 /// Glutathione S-Transferase Mu 2 (Muscle) SEPP1 Selenoprotein P, Plasma, 1

GLRX5 Glutaredoxin 5

GCLC Glutamate-Cysteine Ligase, Catalytic Subunit

MGST3 Microsomal Glutathione S-Transferase 3

SOD2 Superoxide Dismutase 2, Mitochondrial

GSTT1 Glutathione S-Transferase Theta 1

NFE2L1 Nuclear Factor (Erythroid-Derived 2)-Like 1

TXNRD2 Thioredoxin Reductase 2

CCS Copper Chaperone For Superoxide Dismutase

PRDX6 Peroxiredoxin 6

RNF7 Ring Finger Protein 7

SQSTM1 Sequestosome 1

NULL NULL NULL NULL

GSTO2 Glutathione S-Transferase Omega 2

SFTPD Surfactant Protein D SELS Selenoprotein S

GLRX Glutaredoxin (Thioltransferase) PRDX3 Peroxiredoxin 3

TXN Thioredoxin

MPO Myeloperoxidase

GPX1 Glutathione Peroxidase 1

PRDX2 Peroxiredoxin 2

GLRX5 Glutaredoxin 5

SIRT2 Sirtuin (Silent Mating Type Information Regulation 2 Homolog) 2 (S. Cerevisiae) GSTP1 Glutathione S-Transferase Pi 1

OXSR1 Oxidative-Stress Responsive 1

PRDX5 Peroxiredoxin 5

FTH1 Ferritin, Heavy Polypeptide 1

FTH1 Ferritin, Heavy Polypeptide 1

NOS2 Nitric Oxide Synthase 2, Inducible

GSTA2 Glutathione S-Transferase Alpha 2

ATOX1 ATX1 Antioxidant Protein 1 Homolog (Yeast) EPX Eosinophil Peroxidase

NCF2 Neutrophil Cytosolic Factor 2

GSTA1 Glutathione S-Transferase Alpha 1

NCF1 /// NCF1C // Neutrophil Cytosolic Factor 1 /// Neutrophil Cytosolic Factor 1C Pseudogene /// Neutrophil

Cytosolic Factor 1B Pseudogene

GSTM5 Glutathione S-Transferase Mu 5

NULL NULL

CYBB Cytochrome B-245, Beta Polypeptide

AOX1 Aldehyde Oxidase 1

NCF1 /// NCF1C // Neutrophil Cytosolic Factor 1 /// Neutrophil Cytosolic Factor 1C Pseudogene /// Neutrophil

Cytosolic Factor 1B Pseudogene

GSTK1 Glutathione S-Transferase Kappa 1

GLRX2 Glutaredoxin 2

GPX5 Glutathione Peroxidase 5 (Epididymal Androgen-Related Protein) ALOX12 Arachidonate 12-Lipoxygenase

TTN Titin

NCF1 /// NCF1C // Neutrophil Cytosolic Factor 1 /// Neutrophil Cytosolic Factor 1C Pseudogene /// Neutrophil

Cytosolic Factor 1B Pseudogene

APOE /// HMGA1 Apolipoprotein E /// High Mobility Group AT-Hook 1

PTGS1 Prostaglandin-Endoperoxide Synthase 1 (Prostaglandin G/H Synthase And

Cyclooxygenase)

SCARA3 Scavenger Receptor Class A, Member 3

LPO Lactoperoxidase

NFE2 Nuclear Factor (Erythroid-Derived 2), 45kda

GSTM2 /// GSTM4 Glutathione S-Transferase Mu 2 (Muscle) /// Glutathione S-Transferase Mu 4

CCL5 Chemokine (C-C Motif) Ligand 5

GSTA3 Glutathione S-Transferase Alpha 3

GSTT2 /// GSTT2B Glutathione S-Transferase Theta 2 /// Glutathione S-Transferase Theta 2B (Gene/Pseudogene)

GSTT2 /// GSTT2B Glutathione S-Transferase Theta 2 /// Glutathione S-Transferase Theta 2B (Gene/Pseudogene)

KRT1 Keratin 1

DUSP1 Dual Specificity Phosphatase 1

PRDX4 Peroxiredoxin 4

PXDN Peroxidasin Homolog (Drosophila) NQO1 NAD(P)H Dehydrogenase, Quinone 1

GPX6 Glutathione Peroxidase 6 (Olfactory)

BNIP3 BCL2/Adenovirus E1B 19kda Interacting Protein 3

DUOX1 Dual Oxidase 1

GSTA5 Glutathione S-Transferase Alpha 5

GPX4 Glutathione Peroxidase 4 (Phospholipid Hydroperoxidase) GSTM1 Glutathione S-Transferase Mu 1

PNKP Polynucleotide Kinase 3'-Phosphatase

DHCR24 24-Dehydrocholesterol Reductase

MT3 Metallothionein 3

DUOX2 Dual Oxidase 2

ALB Albumin

SOD1 Superoxide Dismutase 1, Soluble GPX3 Glutathione Peroxidase 3 (Plasma) PRNP Prion Protein

TPO Thyroid Peroxidase

GPX7 Glutathione Peroxidase 7

PRDX1 Peroxiredoxin 1

GPX2 Glutathione Peroxidase 2 (Gastrointestinal) TXNRD1 Thioredoxin Reductase 1

SRXN1 Sulfiredoxin 1 Homolog (S. Cerevisiae) SOD3 Superoxide Dismutase 3, Extracellular GLRX3 Glutaredoxin 3

STK25 Serine/Threonine Kinase 25 (STE20 Homolog, Yeast)

PREX1 Phosphatidylinositol-3,4,5-Trisphosphate-Dependent Rac Exchange Factor 1

GTF2I General Transcription Factor Iii GPX8 Glutathione Peroxidase 8 (Putative) CYGB Cytoglobin

OXR1 Oxidation Resistance 1

SRXN1 Sulfiredoxin 1 Homolog (S. Cerevisiae)

UCP2 Uncoupling Protein 2 (Mitochondrial, Proton Carrier) FOXM1 Forkhead Box M1

PTGS2 Prostaglandin-Endoperoxide Synthase 2 (Prostaglandin G/H Synthase And

Cyclooxygenase)

GCLM Glutamate-Cysteine Ligase, Modifier Subunit

HMOX1 Heme Oxygenase (Decycling) 1
